# Perception and acceptance of micronutrient-Fortified Bouillon among Non-Index Household Members: A longitudinal sub-study nested within a randomized trial in Northern Ghana

**DOI:** 10.1371/journal.pone.0345106

**Published:** 2026-04-03

**Authors:** Felix Kwaku Kyereh, Agartha N. Ohemeng, Reina Engle-Stone, K. Ryan Wessells, Sika M. Kumordzie, Charles D. Arnold, Jennie N. Davis, Emily R. Becher, Ahmed D. Fuseini, Kania W. Nyaaba, Xiuping Tan, Stephen A. Vosti, Seth Adu-Afarwuah

**Affiliations:** 1 Department of Nutrition and Food Science, University of Ghana, Legon, Accra, Ghana; 2 Department of Nutrition and Institute for Global Nutrition, University of California, Davis, California, United States of America; 3 Department of Agricultural and Resource Economics, University of California, Davis, California, United States of America; Lusofona University of Humanities and Technologies: Universidade Lusofona de Humanidades e Tecnologias, PORTUGAL

## Abstract

**Background:**

Fortified bouillon cubes offer a scalable strategy to address micronutrient deficiencies in West Africa. However, success depends on household members’ perceptions and acceptance. This study assessed changes in perception and acceptance of fortified bouillon cubes over time and explored factors influencing these outcomes among non-index household members (NIHMs) from trial households in Northern Ghana.

**Methods:**

This longitudinal mixed-methods study was nested within a double-masked randomised controlled trial. Households received micronutrient-fortified bouillon cubes (vitamin A, vitamin B12, folic acid, iron, zinc, iodine) or control cubes (iodine only) for nine months. One NIHM (≥15 years) per household (n = 1,047) was enrolled. Structured interviews were conducted during early (months 1–2; n = 731) and late (months 7–9; n = 796) phases. Composite perception (8 items) and acceptance (10 items) scores (range: 1–5; higher scores = more positive perceptions/greater acceptance) were derived from an 18-item validated instrument. Bayesian linear mixed-effects models assessed temporal changes and associated individual and household factors. Twenty-four focus group discussions (months 4–5) explored drivers and barriers.

**Results:**

Mean age of NIHMs was 40 ± 14.7 years; 59% were female. Perception scores increased over seven months, from 3.4 to 4.1 (adjusted posterior mean difference [aPMD] = 0.69; 95% credible interval [CrI]: 0.57, 0.81). Acceptance remained high (4.59 to 4.58; aPMD = –0.02; 95% CrI: –0.07, 0.03). No differences were observed between trial arms. At early timepoint, perception scores were lower among household heads compared with other household members, and lower among formal employees compared with farmers. Acceptance was higher among individuals from severely food-insecure households and lower among those from mildly food-insecure households. Focus groups revealed that perceived health benefits, sensory appeal, and cultural fit drove acceptance, while infertility-related myths and food colour changes were barriers.

**Conclusion:**

Fortified bouillon cubes were accepted, with perceptions improving over time. Public education will be essential to address misconceptions.

**Trial registration:**

ClinicalTrials.gov (NCT05178407); Pan-African Clinical Trial Registry (PACTR202206868437931). The authors confirm that all ongoing and related trials for this intervention are registered.

## Introduction

Micronutrient deficiencies (MNDs) remain a major public health concern, particularly in low- and middle-income countries (LMICs), where they contribute to anaemia, impaired cognitive development, adverse birth outcomes, and increased susceptibility to infections [1–3]. Women of reproductive age (WRA) and preschool children are especially vulnerable. A pooled analysis of nationally representative data from 24 countries found that 56% of preschool children and 69% of WRA were deficient in at least one core micronutrient (defined as iron, vitamin A, and/or zinc for preschool children, and iron, folate, and/or zinc for WRA) [4].

In Ghana, MNDs are most pronounced in the northern belt regions, where more than 40% of WRA and preschool children are anaemic [5]. These deficiencies are primarily driven by low dietary diversity, a high burden of infections, and persistent socioeconomic inequalities [6–8]. The economic impact is also substantial, with annual losses exceeding 100 million US dollars due to increased healthcare costs and reduced productivity [9]. Addressing MNDs is essential to achieving Sustainable Development Goal 2, which aims to end all forms of malnutrition by 2030 [10].

Recent global modelling of nutrient intake from food, excluding contributions from fortified products and supplements, indicates that 68% of people consume insufficient iodine, 65% insufficient iron, and 54% insufficient folate [11]. The World Health Organization (WHO) recognises large-scale food fortification as a cost-effective and sustainable strategy for reducing MNDs. Meta-analyses have shown that fortification can lower the prevalence of anaemia by 34% and goitre by 74% [12,13].

In Ghana, several national food fortification initiatives have been implemented, including mandatory iodisation of salt, vitamin A fortification of edible vegetable oil, and iron and folic acid fortification of wheat flour. However, the coverage and compliance of these programmes remain limited due to challenges such as weak regulatory enforcement, limited processing capacity, high production costs, and insufficient distribution networks, particularly in rural areas [5,14,15]. National assessments report that only 56% of edible oil and 6% of wheat flour samples meet the required fortification standards, reflecting persistent implementation gaps [5].

Bouillon cubes are used by more than 90% of households in northern Ghana and are typically added during daily meal preparation [16,17]. Their widespread use, habitual consumption, and centralised manufacturing by a few large producers offer a unique opportunity for scalable and standardised fortification [18,19]. Modelling studies from sub-Saharan Africa and Asia suggest that fortifying bouillon cubes with iron, iodine, vitamin A, and zinc could significantly improve the micronutrient status of women and children [20–24].

Despite bouillon cubes’ potential for fortification, the success of such a strategy depends not only on effective nutrient delivery but also on the availability of fortified products, consistent implementation of national policies, and consumer acceptance and use [18,25]. Household perceptions shaped by beliefs, taste preferences, and cultural norms play an essential role in whether fortified products are adopted and consumed regularly [12,26,27], informing both public health strategies and private-sector decisions on product formulation and scale-up. Barriers such as taste, appearance, cost, and cultural beliefs affect both fortified foods and nutrition supplements [28–30]. Although food fortification and nutrition supplements differ in form, both interventions may encounter similar resistance. In northern Ghana, for example, iron and folic acid supplements have been rejected in some communities due to the belief that they cause infertility, a view held by some caregivers and opinion leaders [31].

Though studies have examined acceptability of fortified products among caregivers and direct beneficiaries [32–35], little is known about non-index household members (NIHMs), that is, those who are not enrolled in interventions, but who cook, influence food choices, or hold views that affect household practices [36,37]. Understanding their perceptions is critical, as their attitudes can determine the adoption and sustained use of fortified foods at the household level. Perception refers to how household members understand and evaluate the benefits, safety, and appropriateness of the fortified cubes, whereas acceptance reflects their willingness to use the cubes and incorporate them into routine household cooking.

Building on this gap, the present study, conducted within the Condiment Micronutrient Innovation Trial (CoMIT), assessed changes over time in the perception and acceptance of micronutrient-fortified bouillon cubes and examined the barriers and drivers influencing these outcomes among NIHMs in Northern Ghana.

## Methods

### Condiment Micronutrient Innovation Trial (CoMIT) overview

The CoMIT was a double-blind, community-based randomised controlled trial conducted across 16 sites in the Kumbungu and Tolon districts of the Northern Region of Ghana. The trial evaluated the impact of bouillon cubes fortified with multiple micronutrients (vitamin A, folic acid, vitamin B12, iron, zinc, and iodine) compared with cubes fortified with iodine only, on the nutritional status of women and children.

In brief, eligible households enrolled (n = 1,341) and were randomised (n = 1,312) to receive multiple micronutrients or control cubes for 9 months. Randomisation was conducted at the household level using a computer-generated block randomisation scheme (block sizes of four). Participants drew group assignments from opaque, sequentially numbered, sealed envelopes linked to study codes. To maintain blinding, the fortified and control bouillon cubes were indistinguishable in packaging, labelling (codes H24, L63, K95, M78), appearance, and with no indication of nutrient content. Households, field staff, and data analysts remained blinded to allocation. Allocation codes were generated and managed independently by the University of Ghana and the University of California, Davis. Bouillon cubes were distributed every two weeks for daily household use, with adherence monitored through biweekly household inventory of remaining study cubes and structured questionnaires. Households continued their usual diets and access to routine health services during the intervention; no restrictions on concomitant care were applied. Enrolled index participants included non-pregnant, nonlactating women of reproductive age (WRA, n = 801), preschool children aged 2–5 years (n = 685), and lactating women (n = 666).

The trial received ethical approval from the Ghana Health Service Ethical Review Committee (GHS ERC ID: 024/11/21), the University of California, Davis IRB (ID: 1837253), and the Ghana Food and Drugs Authority (Certificate No. FDA/CT/2213(1)). The trial is registered on ClinicalTrials.gov (NCT05178407) and the Pan-African Clinical Trials Registry (PACTR202206868437931). Written informed consent was obtained from all adult participants prior to enrolment. For participants under 18 years of age, assent was obtained in addition to parental or caregiver consent. All study procedures were conducted in accordance with relevant ethical guidelines and regulatory requirements.

This paper presents findings from a longitudinal sub-study conducted among NIHMs in CoMIT RCT households, aimed at assessing changes in perception and acceptance of the intervention bouillon cubes over the course of the intervention period.

### Study design and data collection time points

This longitudinal mixed-methods study was nested within the CoMIT RCT. Quantitative data collection occurred at three time points: pre-intervention (T0), early intervention (T1; months 1–2), and late intervention (T2; months 7–9). At T0, baseline data were collected from NIHMs living in households with an enrolled WRA, preschool children, and/or lactating woman. At T1 and T2, NIHMs from WRA and preschool-child households completed structured interviews. The T1 survey was conducted after approximately one month of exposure to study cubes. This interval allowed household members sufficient time to use the cubes in routine cooking, ensuring that their reported perceptions and acceptance reflected actual experience rather than expectations formed prior to use. The late-intervention survey (T2) was conducted after seven months of exposure to capture responses following sustained and habitual use. Additionally, 24 focus group discussions (FGDs) were conducted mid-intervention (months 4–5). Data collection spanned January 2023 to May 2024.

### Participants and recruitment

Eligible participants in this sub-study were NIHMs aged 15 years or older who had resided in the household for at least one-month, shared meals with the index participant, and intended to remain throughout the intervention. One NIHM per household was randomly selected using the Kish table method [38], following the household roster compiled during the recruitment of the index participant(s). If the selected person declined and others were eligible, reselection was permitted.

Recruitment occurred within three weeks of the index participant’s recruitment and prior to intervention allocation. Recruitment began on January 15, 2023, and ended on May 31, 2023. Trained interviewers fluent in English and Dagbani obtained written informed consent using a standardised verbal script and five comprehension checks. Consent was documented via participant signature or thumbprint. For participants under 18 years, assent was obtained along with parental or guardian consent. Each NIHM received a unique identifier linked to the household ID.

### Pretesting and quantitative data collection

The questionnaire was pre-tested with 18 purposively selected adults (20–70 years) from two rural communities located 15–20 km from the nearest study sites. These communities, which shared similar cultural and dietary practices with the study population, were excluded from the main data collection to avoid contamination. Participants included household heads, primary cooks, and other adult household members. Feedback was used to revise item clarity, sequencing, and layout [39]. Participants took part in the pre-testing and adaptation of data collection instruments but were not involved in trial design or analysis.

Ten trained fieldworkers administered the final questionnaire electronically using SurveyCTO. Field supervisors conducted daily quality control reviews. Baseline data included demographic characteristics for NIHMs; a household asset-based socio-economic status index; and food insecurity, measured using the Household Food Insecurity Access Scale (HFIAS) [40]. Baseline household commercial bouillon use in the prior 30 days was also recorded, estimated in grams per capita per day using a modified version of the Fortification Assessment Coverage Toolkit (FACT) approach [41]. These variables were extracted from the household-level questionnaire administered during the trial. At T1 and T2, perception was operationalised as participants’ opinions and attitudes regarding the health benefits, safety, and appropriateness of the study cubes. Acceptance was defined as participants’ willingness to consume the cubes and incorporate them into household meals. These constructs were measured using a researcher-developed questionnaire comprising 26 Likert-scale items (1 = completely disagree to 5 = completely agree) and three categorical preference items. Composite scores (range: 1–5, with higher scores indicating more positive perceptions or greater acceptance) were derived for perception (8 items) and acceptance (10 items) from a validated 18-item instrument.

### Qualitative component: focus group discussions (FGDs)

Twenty-four FGDs were conducted with a total of 157 participants. The FGDs consisted of 12 male-only groups and 12 female-only groups, each comprising 6–8 participants. Within each sex category, participants were purposively selected to ensure variation in age (<50 and ≥50 years) and representation from both intervention allocation codes. The FGDs were conducted in Dagbani by trained facilitators using a translated and pre-tested guide grounded in the Theory of Planned Behaviour (TPB) [42] and Health Belief Model (HBM) [43]. Topics included taste and smell, cultural compatibility, perceived health benefits, economic utility, product preferences, and barriers. Sessions lasted approximately two hours and were audio-recorded, with note-takers documenting nonverbal cues. Facilitators held debriefs after each FGD to ensure consistency.

### Composite score derivation for perception and acceptance (outcome variable)

Perception and acceptance were the primary outcome variables and were measured using composite scores derived from a structured, psychometrically validated questionnaire developed for the CoMIT Trial. Confirmatory factor analysis (CFA) supported a two-factor structure comprising perception (8 items; Cronbach’s α = 0.71) and acceptance (10 items; Cronbach’s α = 0.72). Model fit indices indicated good overall fit, including the Chi-square divided by degrees of freedom (χ²/df = 1.91), Root Mean Square Error of Approximation (RMSEA = 0.043), Comparative Fit Index (CFI = 0.981), Tucker–Lewis Index (TLI = 0.977), and Standardised Root Mean Square Residual (SRMR = 0.061) [44]. Item responses were weighted by their standardised CFA loadings (see S1 Table in S1 File for the standardised factor loadings), aggregated, and normalised to generate continuous composite scores ranging from 1 (lowest) to 5 (highest), with higher values reflecting more favorable perceptions or acceptance.

Composite scores were computed separately for the early (T1) and late (T2) intervention time points. All retained items met the minimum loading threshold of 0.40, and both outcomes were treated as continuous variables in subsequent analyses. Although no formal harms or adverse events were pre-specified for this behavioural study, no adverse events related to bouillon consumption were reported.

### Sample size determination

A total of 1,047 NIHMs were enrolled at the pre-intervention time point. These participants came from households with women of reproductive age, preschool children, and/or lactating women. In the parent CoMIT trial, households with a woman of reproductive age and/or a preschool-age child were followed for the full nine-month period, whereas households in which only a lactating woman was enrolled were followed for three months and were not eligible for the repeated measures at T1 and T2. Their household members therefore did not contribute to the longitudinal analysis. At T1 and T2, 731 and 796 participants were interviewed, respectively, with 676 providing complete data at both time points and forming the analytic sample.

Sample size considerations were based on the parent CoMIT RCT, which was powered to detect a minimum standardised mean difference of 0.26 (Cohen’s d) in primary outcomes at α = 0.05 and 80% power. Applying this effect size to the present study indicated that 396 participants (198 per group) would be sufficient to detect a between-group difference of similar magnitude. The analytic sample of 676 complete cases exceeded this threshold, ensuring adequate statistical power for both within-subject and between-group comparisons. Although no established minimal important difference exists for perception or acceptance scores, a standardised mean difference of 0.26 represents a small-to-moderate, and likely meaningful, change in behavioural constructs in nutrition interventions [45].

### Data analysis

#### Protocol adherence and analytical refinements.

This analysis adhered to the design and procedures of the registered CoMIT Trial protocol (NCT05178407; PACTR202206868437931). Analytical methods were guided by the specific Statistical Analysis Plan (SAP), available at https://osf.io/t3zrn/. While the parent protocol provided the general framework, the following refinements were pre-specified in the SAP to address specific analytical constraints: (i) a Bayesian mixed-effects framework was used to resolve convergence failures encountered with frequentist models, while research questions and fixed-effect specifications remained unchanged; (ii) the final 18-item scale was derived via the pre-specified confirmatory factor analysis and excluded items with poor construct validity (<0.40 loading); and (iii) Latent Dirichlet Allocation (LDA) was used as a supplementary validation tool to triangulate the primary deductive thematic analysis.

#### Quantitative analysis.

Descriptive statistics were computed: means and standard deviations for continuous variables, and frequencies and percentages for categorical variables. All continuous predictors were standardised (mean = 0, SD = 1) to facilitate interpretability of regression coefficients [46]. Multicollinearity was assessed using variance inflation factors (VIFs), with all VIFs < 5 indicating no concerns. Raw item scores were compared between intervention and control groups at each time point (early, late) using Welch’s two-sample t-tests for descriptive comparison only. For each item, mean and standard deviation were summarised by group, and unadjusted p-values were reported. Repeated-measures analyses were restricted to participants with complete outcome and covariate data at both T1 and T2 (analytic sample: n = 676). Consistent with brms default behaviour [47], observations with missing values were excluded via listwise deletion. This approach was appropriate for two reasons. First, missingness across all covariates included in the adjusted models was low (<3%), and retention exceeded the thresholds pre-specified in the parent CoMIT Trial protocol for undertaking multiple-imputation sensitivity analyses (>10% missingness in covariates or >20% attrition). Second, attrition in this sub-study was driven primarily by relocation or temporary unavailability rather than characteristics plausibly associated with perception or acceptance outcomes.

To evaluate the potential for attrition-related bias, baseline characteristics of completers were compared with those of non-completers (including households that were later excluded from the trial). No meaningful differences were observed for age, sex, occupation, district, residential strata, or randomisation group (all p > 0.05; **S2 Table** in S1 File), supporting the assumption that data were missing at random (MAR) with respect to key predictors. Given the low overall missingness and comparable baseline profiles between groups, complete-case analysis was unlikely to introduce material bias into the longitudinal estimates.

All inferential analyses were performed using Bayesian linear mixed-effects models. Although the parent trial protocol anticipated the use of frequentist mixed-effects models, the exploratory analyses followed the protocol’s intention to adjust for clustering and to model change over time. However, the frequentist models exhibited singular fits and convergence failures under the planned random-intercept structure, making them unsuitable for reliable estimation. A Bayesian mixed-effects framework was therefore adopted, providing stable estimation, full posterior distributions, and 95% credible intervals that allow direct probabilistic interpretation of effects while addressing the same research questions defined in the protocol. Weakly informative priors were specified for all models: Normal(0, 5) for fixed effects and Student-t(3, 0, 5) for hierarchical intercepts. These priors provide mild regularisation while allowing the data to dominate the posterior. The Normal(0, 5) prior constrains standardised fixed effect estimates to a wide but plausible range, preventing extreme values without imposing strong assumptions. The Student-t prior, with its heavier tails, offers robust shrinkage for random intercepts and is recommended for multilevel models to accommodate potential outliers [47,48]. These priors were not derived empirically from previous datasets but were selected to stabilise estimation while remaining minimally informative. Although estimation was conducted within a Bayesian framework, the analytic structure remained aligned with the protocol’s prespecified principles. Perception and acceptance were modelled as continuous latent-derived scores, time point and intervention arm were included as fixed effects, and clustering was appropriately adjusted through random intercepts.

To evaluate changes in perception and acceptance from T1 to T2, separate models were estimated for each outcome. Initial models included fixed effects for time point (T1 and T2), intervention group, and the time point × group interaction, with a random intercept for participant identity (ID). Because the study was nested within a randomised household-level trial, these terms were retained in the final unadjusted models even when the estimates for the group and interaction effects were not statistically meaningful. Retaining these terms ensures that the modelling approach appropriately reflects the structure of the parent trial and preserves the randomised comparison across groups.

Prior to model specification, community-level clustering was evaluated because multiple households were nested within each community. Intraclass correlation coefficients (ICCs) from unconditional mixed-effects models indicated substantial clustering (perception ICC = 0.49; acceptance ICC = 0.33). Accordingly, all models were re-specified to include a random intercept for Community, in addition to the participant-level random intercept, to account for between-community heterogeneity.

These unadjusted models were then extended by adding covariates selected through a bivariate screening process. Adjusted models included covariates identified through Pearson correlations for continuous variables, independent samples t-tests for binary predictors, and ANOVA for categorical predictors with more than two levels. Covariates with p < 0.10 were retained [49,50]. The adjusted model for perception included baseline household bouillon consumption (g/capita/day), district, HFIAS category [40], and relationship to household head. The adjusted acceptance model included household size, HFIAS category, sex, and residence.

To identify predictors of perception and acceptance, separate Bayesian models were specified for each outcome. All models included fixed effects for time, intervention group, and the set of selected covariates, along with random intercepts for Community and Participant ID. The base model was specified as:


𝐒𝐜𝐨𝐫𝐞~𝐩𝐫𝐞𝐝𝐢𝐜𝐭𝐨𝐫𝐬+𝐢𝐧𝐭𝐞𝐫𝐯𝐞𝐧𝐭𝐢𝐨𝐧 𝐠𝐫𝐨𝐮𝐩+𝐭𝐢𝐦𝐞 + (1 | 𝐂𝐨𝐦𝐦𝐮𝐧𝐢𝐭𝐲) + (1 | 𝐏𝐚𝐫𝐭𝐢𝐜𝐢𝐩𝐚𝐧𝐭 𝐈𝐃).

To assess effect modification by time, interaction terms between time and each predictor were included:


𝐒𝐜𝐨𝐫𝐞 ~ 𝐩𝐫𝐞𝐝𝐢𝐜𝐭𝐨𝐫𝐬+𝐢𝐧𝐭𝐞𝐫𝐯𝐞𝐧𝐭𝐢𝐨𝐧 𝐠𝐫𝐨𝐮𝐩+𝐭𝐢𝐦𝐞 + (𝐭𝐢𝐦𝐞×𝐩𝐫𝐞𝐝𝐢𝐜𝐭𝐨𝐫𝐬) + (1 | 𝐂𝐨𝐦𝐦𝐮𝐧𝐢𝐭𝐲) + (1 | 𝐏𝐚𝐫𝐭𝐢𝐜𝐢𝐩𝐚𝐧𝐭 𝐈𝐃).

Predictors were selected a priori based on theoretical relevance [50]. Individual-level predictors included sex, age, occupation (farmer, homemaker, small business, formal employment), relationship to household head, cooking responsibility, ethnicity, religion, and education. Household-level predictors included household size, baseline household bouillon consumption (g/capita/day), HFIAS category, socioeconomic index (continuous), residential stratum (rural vs. urban), district (Kumbungu vs. Tolon), and sex of the household head. All categorical variables were dummy coded with the first level as the reference category.

Model selection was guided by predictor significance, posterior predictive checks, and leave-one-out cross-validation (LOO-CV) [51]. For perception, LOO-CV indicated that including interaction terms between time point and selected predictors improved model fit over a main-effects model (Δelpd = 18.5, SE = 7.3), so the interaction model was retained. In this model, main effects represent associations at T1 (reference time point), while interaction terms indicate whether the change from T1 to T2 differs across subgroups. For acceptance, the main effects model was preferred (LOOIC = 709.8, SE = 3.1), as interaction terms did not improve model fit.

Sensitivity analyses using alternative prior distributions (Normal(0, 1) for fixed effects and Student-t(3, 0, 1) for intercepts) for both perception and acceptance yielded consistent results, supporting the robustness of the findings (see **S3 Table** in S1 File, panels a–b).

All models were estimated using Hamiltonian Monte Carlo sampling with four chains of 4,000 iterations each, including 1,000 warm-up iterations. Bayesian estimation was conducted in R (version 4.3.3) using the *brms* (v2.22.0) and *bayesplot* (v1.11.1) packages [47,52,53]. Convergence diagnostics included R-hat statistics (< 1.01), effective sample sizes, and visual inspection of trace plots. Posterior predictive checks (*via pp_check*) supported model adequacy. Results are reported as posterior mean differences or estimates with 95% credible intervals (CrI). CrIs excluding zero were interpreted as statistically meaningful [48,54].

### Thematic analysis approach for FGDs

All 24 FGDs, conducted in Dagbani, were transcribed verbatim and translated into English. To ensure fidelity of meaning, 12 transcripts were randomly selected for review by two bilingual researchers, and 6 underwent back-translation to evaluate conceptual equivalence [55,56]. Thematic saturation was determined using the criterion of no new codes or themes emerging in subsequent discussions. Saturation was reached by the 22nd FGD, after which no additional themes were identified. FKK, who was trained in qualitative research methods, developed the interview guide and led the qualitative analysis. KNW’s fluency in Dagbani supported accurate interpretation of participants’ expressions, while FKK’s familiarity with the study communities provided contextual insight during reflexive discussions. SAA, ANO, RES, and SMK reviewed the questionnaire, with SAA and ANO contributing qualitative expertise during theme refinement and interpretation.

Although the protocol specified a deductive thematic analysis guided by the study objectives, several unanticipated views emerged during coding, requiring minor inductive refinement to ensure full representation of participant perspectives. Topic modelling (Latent Dirichlet Allocation) and sentiment analysis were introduced as supplementary triangulation tools to assess the coherence and completeness of manually coded themes. These computational methods did not replace or modify the deductive analytic framework; rather, they served to validate and strengthen it. The primary thematic structure therefore remained consistent with the protocol.

To operationalise the deductive framework, an initial set of codes was developed and then inductively expanded by FKK using six purposively selected transcripts capturing variation in participant sex, age, and intervention allocation. These transcripts were manually coded and thematically analysed following Braun and Clarke’s six-phase framework [57], allowing refinement of code definitions while maintaining alignment with the study objectives. Coding discrepancies were resolved through reflexive discussions between FKK and KNW, with SAA providing input on ambiguous cases to refine code definitions [57,58]. Although reflexive thematic analysis does not require statistical measures of agreement, intercoder comparison was used here as a pragmatic strategy to assess consistency in code application during the early stages of analysis. Intercoder reliability was assessed through independent double-coding of the six transcripts by FKK and KNW, yielding high agreement (Cohen’s Kappa = 0.96; percentage agreement = 97.6%) [59]. Theme-level Kappa values are reported (see S4 Table in S1 File). No further modifications to the codebook were needed.

The full dataset was structured in Microsoft Excel, with each speaker represented in a single row along with metadata (pseudonym, sex, age, education, intervention group). Transcripts were imported into R (v4.3.3) for preprocessing, which included tokenisation, stop-word removal, and lemmatisation [60,61], using the *tidytext, tm,* and *topicmodels* packages [62–64]. Although transcripts were stored and pre-processed at the speaker level, LDA topic modelling was conducted at the FGD level to preserve conversational coherence and shared meaning within group interactions. Each transcript was treated as a separate document for LDA topic modelling [65]. Although LDA is more commonly applied to larger corpora, prior studies demonstrate its suitability for smaller, information-rich qualitative datasets [66–68]. In this study, FGD transcripts ranged from approximately 1,000–3,400 words, with a median length of about 2,400 words, providing sufficient lexical variability for exploratory topic modelling. The optimal number of topics (k = 6) was selected using the highest model coherence score combined with manual interpretability checks, following recommended procedures for topic-model evaluation [69].

LDA was used as a supplementary analytic tool to detect underlying semantic patterns that might not be fully captured through manual coding alone. The integration of deductive and inductive coding with unsupervised topic modelling enhanced the transparency and confirmability of the analysis, consistent with emerging frameworks that combine computational text analysis with human interpretation [70,71]. Although the corpus was relatively small, LDA provided a reproducible means of assessing the coherence of emergent themes and triangulating manually generated codes, thereby strengthening methodological rigour.

Topics derived from LDA were systematically compared with manually generated themes. Several topics, including affordability, health concerns, and cultural familiarity, aligned closely with inductive themes, while an additional theme, “practical experience,” was revealed through topic modelling and subsequently confirmed through transcript re-examination and reflexive team discussion. This theme was incorporated into the final thematic framework following transcript re-examination and team consensus. Final interpretation and consolidation of themes were undertaken jointly by FKK, SAA, ANO, and RES to ensure contextual accuracy and coherence.

To further examine emotional and evaluative content, sentiment analysis was conducted using the National Research Council (NRC) lexicon [72,73], which classifies terms by emotional valence and polarity. Sentiment scores were used to contextualise whether participants’ narratives reflected positive, negative, or mixed attitudes, thereby enriching the interpretation of themes. Findings from thematic analysis, topic modelling, and sentiment analysis were integrated with quantitative results at the interpretation stage, consistent with a convergent mixed-methods design [74].

Further details of the trial design and procedures are available in the published protocol [75], which is also provided as Supporting information (S5 Protocol in S1 File).

## Results

**Fig 1:**
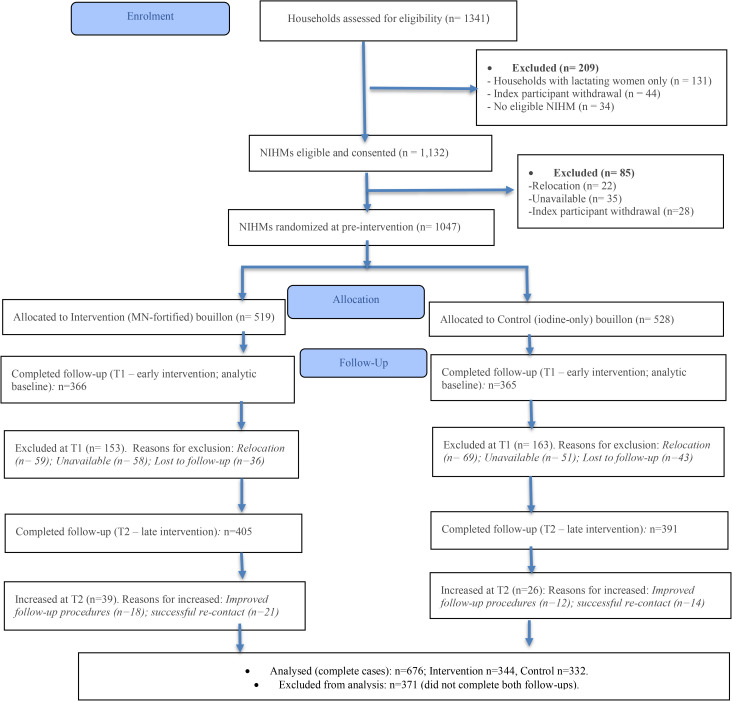
CONSORT flow diagram of households and non-index household members (NIHMs) through the trial. NIHMs = non-index household members; T1 = early intervention; T2 = late intervention. The diagram summarizes the flow of households assessed for eligibility, excluded, randomly allocated, and follow-up at early (T1) and late (T2) time points, and final analytic sample (n = 676 complete cases). Reasons for attrition at each stage (relocation, unavailability, and loss to follow-up) are detailed for both intervention and control arms.

**Fig 1** shows the CONSORT flow diagram of households and NIHMs through the trial. Of the 1,341 households assessed for eligibility, 1,132 were eligible and consented to participate. A total of 1,047 NIHMs were randomised to receive multiple-micronutrient–fortified bouillon cubes (n = 519) or iodine-only control cubes (n = 528).

At the early intervention time point (T1; analytic baseline), 366 intervention and 365 control NIHMs completed follow-up, with losses primarily due to relocation, temporary unavailability, or loss to follow-up. At the late intervention time point (T2), 405 intervention and 391 control NIHMs completed follow-up, reflecting improved re-contact and follow-up procedures.

Overall, 676 participants (344 intervention; 332 control) provided complete data at both T1 and T2 and were included in the longitudinal analysis, while 371 NIHMs were excluded due to incomplete follow-up. Baseline characteristics of completers (n = 676) and non-completers (n = 371) did not differ significantly by age, sex, occupation, randomisation group, district, or rural/urban strata (all p > 0.05), indicating minimal attrition bias (see **S2 Table** in S1 File).

Data collection occurred between January 2023 and May 2024. The T1 survey was conducted after approximately one month of household exposure to the study cubes, and the T2 survey occurred seven months later, near the end of the nine-month intervention period.

### Background characteristics

Baseline characteristics of NIHMs were generally balanced across intervention and control groups (**Table 1**). The mean age of participants was 39.5 years in the intervention group and 40.5 years in the control group. Women comprised the majority in both groups (59.0% vs. 59.1%). Most participants were wives of household heads (37.0% vs. 38.6%) or reported other relationships such as children, in-laws, or siblings (26.2% vs. 20.5%). Approximately three-quarters of participants in both arms had no formal education, and nearly all were of Dagomba ethnicity (>98%) and Muslim (>98%). Agriculture and small business were the most common occupations in both groups. Household characteristics were also comparable, with a mean household size of 11.4 in the intervention group and 11.3 in the control group. Food insecurity was prevalent, with more than half of households classified as moderately or severely food insecure in both groups. Average baseline household bouillon consumption was 3.2 g/capita/day in the intervention arm and 2.9 g/capita/day in the control arm.

**Table 1:** Baseline household and individual-level characteristics of non-index household members by randomisation group.

Variable	Intervention (n = 519) n (%)	Control (n = 528) n (%)
**Age, years *(mean ± SD)***	39.5 ± 14.7	40.5 ± 14.6
**Sex**		
Male	213 (41.0)	216 (40.9)
Female	306 (59.0)	312 (59.1)
**Relationship with household head (HH)**		
Self	130 (25.0)	165 (31.2)
Wife of HH	192 (37.0)	204 (38.6)
Parent of HH	61 (11.8)	51 (9.7)
Others^2^	136 (26.2)	108 (20.5)
**Household cooking role**		
Never cook	207 (39.9)	220 (41.7)
Primary cook	196 (37.8)	186 (35.2)
Secondary/occasional	116 (22.4)	122 (23.1)
**Educational level**		
No formal education	381 (73.4)	399 (75.6)
Basic education	67 (12.9)	66 (12.5)
Senior high school or higher	71 (13.7)	63 (11.9)
**Ethnicity**		
Dagomba	517 (100.0)	525 (100.0)
Religion		
Islam	513 (98.8)	519 (98.3)
Others^4^	6 (1.2)	9 (1.7)
**Occupation**		
Agricultural/farming	236 (45.5)	239 (45.3)
Homemaker	84 (16.2)	94 (17.8)
Self-employed (small business owners)	158 (30.4)	155 (29.4)
Government/private employees (formal employees)	41 (7.9)	40 (7.6)
**District**		
Kumbungu	248 (47.8)	240 (45.5)
Tolon	271 (52.2)	288 (54.5)
**Strata**		
Rural	264 (50.9)	279 (52.8)
Urban	255 (49.1)	249 (47.2)
**Household head education level**		
No formal education	398 (77.7)	405 (77.7)
Primary	55 (10.7)	52 (10.0)
Secondary or higher	59 (11.5)	64 (12.3)
**Household head sex**		
Male	482 (93.6)	516 (97.7)
Female	33 (6.4)	12 (2.3)
Household size (mean ± SD)	11.4 ± 5.9	11.3 ± 5.8
**Household food insecurity access scale (HFIAS)**		
Food secure	210 (40.5)	214 (40.5)
Mild food insecure	94 (18.1)	80 (15.2)
Moderate food insecure	164 (31.6)	190 (36.0)
Severe food insecure	51 (9.8)	44 (8.3)
**Baseline household bouillon consumption** (g/capita/day) (mean ± SD)	3.2 ± 7.4	2.9 ± 4.0

**Note:** Participants include non-enrolled household members aged 15 years and older from households participating in the Condiment Micronutrient Innovation Trial (CoMIT). Household food insecurity was measured using the Household food insecurity access scale (HFIAS) [40]. Baseline household bouillon consumption was calculated as total grams used per day divided by household size, resulting in grams per capita per day. Values are presented as mean ± SD for continuous variables and n (%) for categorical variables. No statistical tests of baseline differences were performed, consistent with CONSORT recommendations. The category “Others” under relationship to household head includes sons or daughters (n = 102), daughters-in-law (n = 88), and siblings (n = 54). “Others” under religion includes Christianity (n = 10) and Traditionalist (n = 5).

**Abbreviations:** HH, household head; HFIAS, household food insecurity access scale.

### Item-level responses on perception and acceptance questionnaire

Table 2 compares raw item scores between the intervention and control groups and shows no meaningful differences at either time point. At the early intervention time point, mean scores for all items were similar between the two groups, with *p*-values ranging from 0.04 to 0.93. The only item showing a marginal difference was Q20 (“Enjoy foods prepared with study bouillon cubes”), where the intervention group reported slightly lower scores than the control group (*p* = 0.040). All other differences were small and not statistically significant.

**Table 2:** Raw item scores for intervention and control groups at early and late intervention time points.

Question (item)	Control Mean (SD)	Intervention Mean (SD)	p value
**Early intervention**			
Q1- Views about bouillon cubes unchanged since receiving study cubes	2.87 (1.44)	2.80 (1.40)	0.535
Q2- Okay to use the study bouillon cubes for everyone	4.67 (0.58)	4.63 (0.59)	0.356
Q3- Study bouillon smells similar as regular bouillon cubes	2.95 (1.45)	2.91 (1.46)	0.706
Q4- Study bouillon tastes similar as regular bouillon cubes	2.80 (1.56)	2.83 (1.52)	0.788
Q5- Study bouillon can be used daily in household cooking	4.62 (0.58)	4.56 (0.54)	0.176
Q6- Study bouillon to be used only on some days	3.38 (1.38)	3.33 (1.42)	0.647
Q10- Agree with those who think it’s good to use study bouillon cubes	4.70 (0.71)	4.67 (0.60)	0.547
Q16- Likes the smell of study bouillon cubes	4.36 (0.76)	4.34 (0.72)	0.630
Q17- Likes the taste of study bouillon cubes	4.66 (0.52)	4.59 (0.60)	0.080
Q18- Happy household receives study bouillon cubes	4.67 (0.50)	4.63 (0.52)	0.286
Q20- Enjoy foods prepared with study bouillon cubes	4.70 (0.54)	4.62 (0.52)	0.040*
Q23- No personal problems observed with bouillon cubes	4.13 (1.27)	3.96 (1.37)	0.082
Q24- No problems observed by household members during using the study cubes	4.16 (1.32)	4.12 (1.32)	0.693
Q25- Positive views about study bouillon cubes	4.58 (0.61)	4.54 (0.57)	0.394
Q26- Does not want household to continue using bouillon cubes	3.74 (1.55)	3.53 (1.59)	0.071
Q27- Thinks neighbors/friends would not like the study bouillon cubes	3.52 (1.52)	3.39 (1.52)	0.269
Q28- Wants household to use the study bouillon in future	4.55 (0.60)	4.50 (0.58)	0.221
Q29- Would buy the study bouillon if sold in future	4.89 (4.97)	4.61 (0.49)	0.292
**Late intervention**			
Q1- Views about bouillon cubes unchanged since receiving study cubes	3.66 (1.01)	3.60 (1.08)	0.480
Q2- Okay to use the study bouillon cubes for everyone	4.52 (0.62)	4.51 (0.64)	0.923
Q3- Study bouillon smells similar as regular bouillon cubes	3.78 (1.30)	3.79 (1.30)	0.914
Q4- Study bouillon tastes similar as regular bouillon cubes	3.82 (1.34)	3.73 (1.38)	0.377
Q5- Study bouillon can be used daily in household cooking	4.35 (0.62)	4.38 (0.59)	0.453
Q6- Study bouillon to be used only on some days	3.80 (0.90)	3.72 (0.92)	0.203
Q10- Agree with those who think it’s good to use study bouillon cubes	4.75 (0.50)	4.75 (0.48)	0.858
Q16- Likes the smell of study bouillon cubes	4.54 (0.62)	4.51 (0.70)	0.617
Q17- Likes the taste of study bouillon cubes	4.68 (0.57)	4.64 (0.61)	0.296
Q18- Happy household receives study bouillon cubes	4.71 (0.46)	4.64 (0.58)	0.049*
Q20- Enjoy foods prepared with study bouillon cubes	4.67 (0.51)	4.65 (0.60)	0.528
Q23- No personal problems observed with bouillon cubes	4.63 (0.68)	4.61 (0.70)	0.694
Q24- No problems observed by household members during using the study cubes	4.61 (0.73)	4.61 (0.73)	0.980
Q25- Positive views about study bouillon cubes	4.56 (0.51)	4.53 (0.56)	0.332
Q26- Does not want household to continue using bouillon cube	4.33 (0.86)	4.27 (0.93)	0.358
Q27- Thinks neighbours/friends would not like the study bouillon cubes	4.39 (0.82)	4.23 (0.96)	0.010*
Q28- Wants household to use the study bouillon in future	4.51 (0.51)	4.48 (0.60)	0.410
Q29- Would buy the study bouillon if sold in future	4.58 (0.51)	4.52 (0.57)	0.081

**Footnote:** Values are means with standard deviations in parentheses. Statistical comparisons between intervention and control groups were performed using Welch’s two-sample t-test at each timepoint. The reported p-values correspond to unadjusted tests and are provided for descriptive interpretation only.

At the late intervention time point, item-level scores again remained comparable across groups, with *p*-values ranging from 0.01 to 0.98. One item (Q27: “Thinks neighbours/friends would not like the study bouillon cubes”) showed a modest difference favouring the control group (*p* = 0.010). Across both timepoints, the direction and magnitude of differences were small, and the overall pattern indicates that intervention and control groups expressed largely similar perceptions and acceptance of the study cubes.

### Change in perception and acceptance of the intervention bouillon cubes among non-index household members in the CoMIT project

#### Change in perception.

**Table 3** presents the change in perception scores between the early (T1) and late (T2) intervention time points. A total of 676 NIHMs contributed data at both time points and were analysed. Mean perception scores increased from 3.4 (SD = 1.1) at T1 to 4.1 (SD = 0.6) at T2. Because scores range from 1 to 5, with higher values indicating more favourable perceptions, this reflects a clear increase over time.

**Table 3:** Change in perception of study-supplied bouillon cubes between early (T1) and late (T2) intervention time points.

Variables	Posterior mean difference (PMD)	95% Credible interval (CrI)	Raw mean (mean ± SD)
**Unadjusted model**			
Time point (T2 vs. T1)	0.70	0.58, 0.82	T1: 3.4 ± 1.1; T2: 4.1 ± 0.6
SD (community intercept)	0.36	0.27, 0.49	NA
SD (participant intercept)	0.03	0.00, 0.08	NA
**Adjusted model**			
Time point (T2 vs. T1)	0.69	0.57, 0.81	
SD (community intercept)	0.37	0.27, 0.51	NA
SD (participant intercept)	0.03	0.00, 0.08	NA
Time × group interaction	−0.04	−0.21, 0.12	NA

**Note:** Study-supplied bouillon cubes included either multiple micronutrient-fortified cubes (containing vitamins A and B12, folic acid, iron, zinc, and iodine) or control cubes fortified with iodine only. Posterior mean difference (PMD) represents model-estimated mean differences in perception scores derived from Bayesian linear mixed-effects models with random intercepts for participants. A 95% credible interval (CrI) that does not include 0 indicates statistical significance. Study-supplied bouillon cubes included either multiple micronutrient-fortified cubes (vitamins A and B12, folic acid, iron, zinc, and iodine) or control cubes fortified with iodine only. T1 represents the early intervention time point (second month of household exposure), and T2 represents the late intervention time point (final two months of the nine-month intervention). Perception scores range from 1 to 5, with higher values indicating more favorable perceptions. The adjusted model includes covariates for district, relationship to the household head, baseline household bouillon consumption (g/capita/day), cooking responsibility, and sex of the household head.

**Abbreviations**: CrI, credible interval.

In the unadjusted Bayesian multilevel linear model, which included random intercepts for community and participant, perception scores were higher at T2 compared with T1 (posterior mean difference [PMD] = 0.70; 95% CrI: 0.58, 0.82). Results were similar in the adjusted model, which controlled for baseline household bouillon consumption, district, relationship to the household head, cooking responsibility, and participant sex. The estimated adjusted PMD between time points remained (adjusted PMD = 0.69; 95% CrI: 0.57, 0.81). Although no established minimal important difference exists for perception scores, the standardised posterior mean difference corresponding to this T1–T2 contrast (approximately 0.65 SD) represents a moderate behavioural change, suggesting that repeated exposure meaningfully improved favourability toward the cubes. There was no evidence of a difference in change over time by study arm (time × group interaction: interaction PMD = −0.04; 95% CrI: −0.21, 0.12).

### Change in acceptance

**Table 4** presents change in acceptance between T1 and T2. Mean acceptance remained high at both time points (early: 4.59 [SD = 0.38]; late: 4.58 [SD = 0.35]), indicating consistently strong acceptance of the study bouillon cubes. The very small posterior mean difference (PMD) of –0.02 is consistent with a ceiling effect, as acceptance scores were already close to the upper limit of the scale at T1.

**Table 4:** Change in acceptance of study-supplied bouillon cubes between early (T1) and late (T2) intervention time points.

Variables	Posterior mean difference (PMD)	95% Credible interval (CrI)	Raw mean (mean ± SD)
**Unadjusted model**			
Time point (T2 vs. T1)	−0.02	−0.07, 0.03	T1: 4.59 ± 0.38; T2: 4.58 ± 0.35
SD (community intercept)	0.16	0.12, 0.22	NA
SD (participant intercept)	0.02	0.00, 0.04	NA
**Adjusted model**			
Time point (T2 vs. T1)	−0.02	−0.07, 0.04	
SD (community intercept)	0.16	0.12, 0.23	NA
SD (participant intercept)	0.04	0.00, 0.09	NA
Time × group interaction	0.04	−0.03, 0.11	NA

**Note:** Study-supplied bouillon cubes included either multiple micronutrient-fortified cubes (containing vitamins A and B12, folic acid, iron, zinc, and iodine) or control cubes fortified with iodine only. Posterior mean difference (PMD) represents model-estimated mean differences in acceptance scores derived from Bayesian linear mixed-effects models with random intercepts for participants. A 95% credible interval (CrI) that does not include 0 indicates statistical significance. Study-supplied bouillon cubes included either multiple micronutrient-fortified cubes (vitamins A and B12, folic acid, iron, zinc, and iodine) or control cubes fortified with iodine only. T1 represents the early intervention time point (data collected during the second month of household exposure), and T2 represents the late intervention time point (data collected during the final two months of the nine-month intervention). Acceptance scores range from 1 to 5, with higher values indicating stronger acceptance. The adjusted model includes covariates for household food insecurity access score (HFIAS), household size, participant sex, residential strata (urban/rural), religion, and baseline household bouillon consumption (g/capita/day).

**Abbreviations:** CrI, credible interval; SD, standard deviation

In the unadjusted Bayesian multilevel linear model, which included random intercepts for community and participant, acceptance did not differ meaningfully between time points (PMD = −0.02; 95% CrI: −0.07, 0.03). Results were similar in the adjusted model, which controlled for household size, household food insecurity, participant sex, residential strata, and baseline household bouillon consumption. The estimated adjusted PMD between time points remained small (adjusted PMD = −0.02; 95% CrI: −0.07, 0.03), and the time × group interaction did not change the interpretation (interaction PMD = 0.04; 95% CrI: −0.03, 0.11). No adverse events related to bouillon consumption were reported during the study period.

### Individual and household factors associated with the perception towards the study-supplied bouillon cubes

Perception scores among NIHMs varied according to individual and household characteristics, and these associations differed between the early (T1) and late (T2) intervention time points (**Table 5**).

**Table 5:** Individual and household factors associated with perception of study-supplied bouillon cubes among non-index household members at early (T1) and late (T2) intervention time points in the randomised trial.

Variable	Categorical levels	Posterior mean estimate (PME)	95% Credible interval (CrI)
**Individual-level factors**			
Occupation	Farming	***Ref.***	
	Homemaker	−0.13	−0.32, 0.07
	Small business owners	−0.12	−0.29, 0.04
	Formal employees (government/private)	−0.37	−0.63, −0.12*
Participant relationship with household heads (HH)	HH	***Ref.***	
	Wives	0.40	0.18, 0.62*
	Sons/daughters	0.28	0.03, 0.52*
	In-laws/siblings	0.32	0.08, 0.56*
	Parents	0.37	0.14, 0.61*
Sex	Female	***Ref.***	
	Male	−0.01	−0.21, 0.20
Household cooking role	Never cooks	***Ref.***	
	Primary cooks	0.06	−0.14, 0.26
	Secondary/occasional cooks	0.08	−0.10, 0.26
**Household-level factors**			
Strata	Rural	***Ref.***	
	Urban	−0.04	−0.33, 0.24
District	Kumbungu	***Ref.***	
	Tolon	0.08	−0.21, 0.39
Sex of HH	Female	***Ref.***	
	Male	−0.13	−0.34, 0.08
Socioeconomic index	Continuous	−0.01	−0.05, 0.05
Baseline household bouillon consumption (g/capita/day)	Continuous	−0.04	−0.08, 0.01
**Time point and interaction effect**			
Interaction: Late × district	Kumbungu	***Ref.***	
	Tolon	−0.26	−0.42, −0.09*
Interaction: Late × occupation	Farming	***Ref.***	
	Homemaker	0.14	−0.12, 0.41
	Small business owners	0.28	0.07, 0.50*
	Formal employees	0.68	0.34, 1.03*
Interaction: Late × relationship with HH	Household head	***Ref.***	
	Wife	−0.65	−0.89, −0.42*
	Sons/daughters	−0.32	−0.65, 0.01
	Parents	−0.43	−0.72, −0.13*
	In-laws/siblings	−0.56	−0.88, −0.24*
Random effect (community level)	SD (intercept)	0.38	0.28, 0.52
Random effect (participant level)	SD (intercept)	0.03	0.00, 0.08

**Note:** Study-supplied bouillon cubes included either multiple micronutrient-fortified cubes (containing vitamins A and B12, folic acid, iron, zinc, and iodine) or control cubes fortified with iodine only. Perception scores range from 1 to 5, with higher scores indicating more favorable perceptions. Study-supplied bouillon cubes consisted of either multiple micronutrient-fortified cubes (vitamins A and B12, folic acid, iron, zinc, and iodine) or control cubes fortified with iodine only. T1 represents early intervention (second month of household exposure), and T2 represents late intervention (final two months of the nine-month intervention). Estimates were derived from Bayesian linear mixed-effects models with random intercepts for community and participant. Main effects represent associations at T1 (reference timepoint). Interaction terms indicate differences in change over time: negative values represent smaller increases from T1 to T2 relative to the reference group, and positive values represent larger increases. CrIs that do not include zero indicate statistical significance (*). Adjustment variables (intervention group and timepoint) were included to account for trial design and repeated measures but were not predictors of interest. Socioeconomic index was derived from a composite asset score. Baseline household bouillon consumption was calculated as grams used per day divided by household size. Farmers served as the reference category for occupation.

**Abbreviations:** HH, household head; CrI, credible interval; SD, standard deviation

At T1 (reference time point), some individual characteristics were associated with higher perception scores. Compared with household heads, higher scores were observed among wives, (posterior mean estimate [PME] = 0.40; 95% CrI: 0.18, 0.62), sons and daughters (PME = 0.28; 95% CrI: 0.03, 0.52), in-laws and siblings (PME = 0.32; 95% CrI: 0.08, 0.56), and parents (PME = 0.37; 95% CrI: 0.14, 0.61). Formal employees had lower perception scores than farmers (PME = –0.37; 95% CrI: –0.63, –0.12), while homemakers and small business owners did not differ meaningfully from farmers. Perception at T1 did not differ by participant sex or household cooking role.

At the household level, none of the baseline characteristics, including residential strata, district, socioeconomic index, sex of the household head, or baseline household bouillon consumption, showed meaningful associations at T1 because all CrIs included zero.

Perception increased between T1 and T2, but the size of the increase differed across subgroups. Participants residing in Tolon showed a smaller increase compared with those in Kumbungu (interaction PME = –0.26; 95% CrI: –0.42, –0.09). Changes over time also differed by occupation. Small business owners (PME = 0.28; 95% CrI: 0.07, 0.50) and formal employees (PME = 0.68; 95% CrI: 0.34, 1.03) experienced larger increases relative to farmers, whereas homemakers did not differ meaningfully. Differences were also observed by relationship to the household head. Compared with household heads, wives (interaction PME = –0.65; 95% CrI: –0.89, –0.42), parents (PME = –0.43; 95% CrI: –0.72, –0.13), and in-laws and siblings (PME = –0.56; 95% CrI: –0.88, –0.24) experienced smaller increases between T1 and T2. For sons and daughters, the CrI slightly overlapped zero but suggested a similar pattern (PME = –0.32; 95% CrI: –0.65, 0.01).

### Individual and household factors associated with acceptance of the study-supplied bouillon cubes

Individual and household factors associated with acceptance scores among NIHMs are presented in **Table 6**. Acceptance did not differ by sex, as males and females reported similar scores (posterior mean estimate [PME] = 0.02; 95% CrI: –0.01, 0.06). Occupational category was also not meaningfully associated with acceptance. Homemakers, small business owners, and formal employees had scores comparable to farmers, the reference group. Religious affiliation likewise showed no meaningful association (PME = 0.08; 95% CrI: –0.07, 0.24).

**Table 6:** Individual and household factors associated with acceptance of study-supplied bouillon cubes among non-index household members at early (T1) and late (T2) intervention time points in the randomised trial.

Variable	Categorical levels	Posterior mean estimate (PME)	95% Credible interval (CrI)
Sex	Female	***Ref.***	
	Male	0.02	−0.01, 0.06
Occupation	Farming	***Ref.***	
	Homemaker	0.03	−0.04, 0.09
	Small business owners	0.02	−0.04, 0.07
	Formal employees	0.01	−0.07, 0.09
Religion	Islam	***Ref.***	
	Christianity/Traditionist	0.08	−0.07, 0.24
Strata	Rural	***Ref.***	
	Urban	0.01	−0.11, 0.14
Household food insecurity	Food Secure	***Ref.***	
	Mild food insecure	−0.06	−0.12, −0.00*
	Moderate food insecure	−0.01	−0.06, 0.03
	Severe food insecure	0.08	0.01, 0.16*
Baseline household bouillon consumption (g/capita/day)	Continuous	−0.02	−0.04, −0.00*
Socioeconomic Index	Continuous	0.01	−0.01, 0.03
Random Effect (Community-level)	SD (Intercept)	0.16	0.12, 0.22
Random Effect (Participant-level)	SD (Intercept)	0.02	0.00, 0.04

**Note:** Study-supplied bouillon cubes included either multiple micronutrient-fortified cubes (containing vitamins A and B12, folic acid, iron, zinc, and iodine) or control cubes fortified with iodine only. T1 represents the early intervention data collection time point (second month of household exposure), and T2 represents the late intervention data collection time point (final two months of the nine-month intervention). Posterior means and 95% credible intervals (CrIs) were obtained from Bayesian linear mixed-effects models with random intercepts for community and participant. A CrI that does not include zero indicates statistical significance (*). Adjustment variables (time point and intervention group) were included to account for the trial design. The acceptance composite score ranges from 1 to 5, with higher values indicating stronger acceptance of the bouillon cubes. The socioeconomic index was derived from a composite household asset score. Baseline household bouillon consumption was calculated as grams used per day divided by household size. Farmers served as the reference category for occupation, as they represented most participants.

**Abbreviations:** CrI, credible interval; SD, standard deviation

At the household level, acceptance did not differ by residential strata or district of residence. However, household food insecurity showed variation in acceptance levels. Compared with food-secure households, mildly food-insecure households had slightly lower scores (PME = –0.06; 95% CrI: –0.12, –0.00), whereas severely food-insecure households had higher scores (PME = 0.08; 95% CrI: 0.01, 0.16). Moderately food-insecure households did not differ from the food-secure group. Baseline household bouillon consumption (g/capita/day) was associated with slightly lower acceptance (PME = –0.02; 95% CrI: –0.04, –0.00). Socio-economic status was not meaningfully associated with acceptance (PME = 0.01; 95% CrI: –0.01, 0.03).

Acceptance levels were stable across demographic and socio-economic groups, with only household food insecurity and baseline household bouillon consumption showing small but detectable associations.

### Qualitative results

A total of 24 FGDs were conducted with 157 NIHMs, comprising 74 men and 83 women. Thematic analysis yielded five major themes reflecting drivers and barriers to acceptance of the study-supplied bouillon cubes. Participant characteristics are presented in **S6 Table** in S1 File, and illustrative quotations for each theme are included in **S7 File** in S1 File.

#### Theme 1: Health and well-being.

Participants reported noticeable reductions in minor ailments such as stomach pains, headaches, fever, and diarrhoea, especially among children. Female participants often attributed these improvements to the study-supplied bouillon cubes, citing fewer hospital visits and improved blood health in their children.

One participant shared, *“Since we started using the study-supplied bouillon cubes, I’ve noticed my children fall sick less often. Their energy levels have improved significantly”* (Female participant, Household head’s wife, Homemaker).

Another highlighted benefits for older adults: *“The study cubes have been a blessing for my family. My elderly parents feel more energetic and less fatigued”* (Male participant, Household head, Small Business Owner).

#### Theme 2: Sensory and practical experiences.

Many participants described the study cubes as having a balanced flavour profile, being mildly salty, and dissolving easily during cooking. The cubes were perceived as compatible with local dishes and often preferred over commercial alternatives due to their taste and ease of use.

One participant remarked, *“The taste of the study cubes is perfect for our traditional soups and stews. It’s not too salty or overpowering”* (Female participant, Household head’s wife, Homemaker).

Another participant stated that *“We love the flavour of the study cubes. They make our meals delicious the way we want the food to taste”* (Male participant, Household head).

#### Theme 3: Cultural compatibility and cooking habits.

Participants reported that the study cubes integrated well into traditional meal preparation. They noted that the cubes did not alter the original taste of familiar dishes, which was seen as an important factor for sustained use.

As one participant explained, *“The study cubes fit perfectly with our traditional recipes. They don’t change the original taste of our dishes”* (Female participant, Household head’s wife, Small Business Owner).

#### Theme 4: Economic benefits influencing acceptance.

Economic considerations were prominent, particularly among male participants. The cubes were seen as reducing expenditure on condiments and healthcare. Many NIHMs appreciated receiving the cubes at no cost, linking this to improved affordability and household budgeting.

One male participant shared, *“Switching to the study cubes has been great for our budget. They’re free and help our blood levels because we no longer fall sick frequently”* (Male participant, Household head).

Similarly, a female participant remarked, *“We have cut down on healthcare costs since using the study-supplied cubes. They’re an economical choice for our family because I no longer have blood shortage”* (Female participant, Household head’s wife, Formal Employee).

#### Theme 5: Perceived challenges and barriers to acceptance.

While overall acceptance was high, some participants reported minor concerns that influenced their initial perceptions of the study-supplied bouillon cubes. Changes in food colour, particularly the darkening of soups prepared with green leafy vegetables, were noted in five of the 24 FGDs. A few participants expressed concerns about long-term health effects, including fears related to male infertility.

One participant shared, *“Initially, I believed the study cubes might act like family planning and reduce our sexual performance, but over time I realized I could perform even better”* (Male participant, Household head, Farmer).

Another remarked, *“Sometimes the intervention cubes change the colour of our soups to dark, especially the green leafy ones. It’s not a major issue, but it’s noticeable”* (Female participant, Household head’s wife, Small Business Owner).

### Overall drivers of acceptance and usage

Across demographic groups, participants reported using the study-supplied bouillon cubes and expressed a preference for them over commercial alternatives. Reported drivers of use included perceived health benefits, convenience, sensory appeal, and financial considerations. The cubes were reportedly used in a wide variety of meals.

### Sentiment analysis

Participants’ narratives reflected predominantly positive sentiments towards the study supplied bouillon cubes. The most frequent sentiments were *trust* and *joy*, often linked to perceived health improvements, satisfaction with taste, and economic benefits. For example, one participant noted, *“We know the study cubes have improved our health and well-being, which makes us* believe *they are beneficial”* (Female participant, Household head’s wife).

Negative sentiments were relatively rare and were usually related to uncertainty about potential long-term effects. For example: “*I am just cautious about how the fortified cubes might affect us in the long run”* (Male participant, Household head, Farmer).

The sentiment category of anticipation reflected statements that expressed optimism about future use, as one participant said: *“We will keep buying and restocking the study cubes in our homes if we can find them on the market”* (Female participant, Household head’s wife).

The category of surprise did not produce clear or coherent quotations in the transcripts; the automated lexicon assigned this label to isolated words that did not represent genuine expressions of surprise. For this reason, we interpreted anticipation but did not attribute analytic meaning to the surprise category.

**Fig 2:**
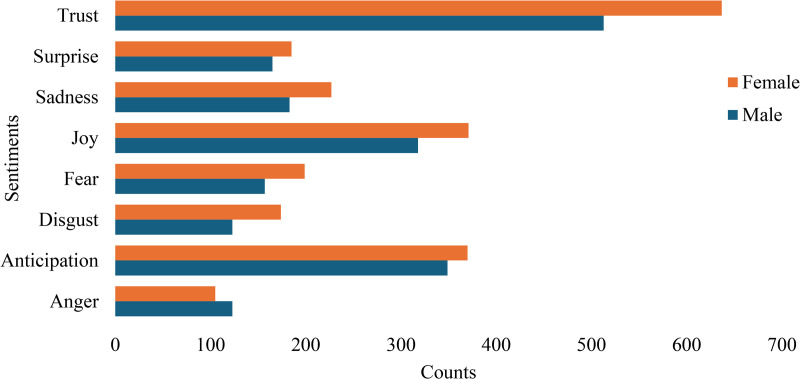
Distribution of sentiment responses related to health and well-being, disaggregated by sex. Bars represent the number of times each sentiment category was expressed in focus group discussion transcripts based on automated sentiment analysis using the NRC Word–Emotion Lexicon. Sentiments are shown separately for male and female participants, with higher bar lengths indicating greater frequency of that sentiment within the transcripts.

**Fig 2** presents trends in emotional valence and sentiment categories related to health and well-being, disaggregated by sex.

## Discussion

This study examined initial levels and longitudinal changes in perception and acceptance of fortified bouillon cubes among non-index household members (NIHMs) in a randomised household-level intervention in northern Ghana. It also assessed individual- and household-level factors associated with these outcomes and explored the contextual influences shaping these behavioural constructs. Perception reflects the beliefs and attitudes individuals hold about the study cubes, whereas acceptance represents the behavioural decision to use them in daily cooking. Although related, these constructs represent distinct dimensions of behaviour that are influenced by different determinants.

Perception increased significantly over the intervention period, reaching a mean score of 4.1 on a 1–5 scale, whereas acceptance remained consistently high at both time points. The improvement in perception (≈0.70 points) represents a moderate and behaviourally meaningful shift within a relatively short exposure window. In contrast, limited change in acceptance likely reflects a ceiling effect, as acceptance at the early intervention time point (T1) was already high rather than indicating an absence of behavioural responsiveness.

Subgroup analyses revealed notable variation in changes in perception over time. At T1, other household members such as wives, parents, and siblings reported more favourable perceptions than household heads, although household heads showed greater improvement over time. Formal employees and small business owners also showed larger increases in perception compared with farmers. Acceptance varied little across groups, except for slightly higher acceptance among individuals from severely food-insecure households and marginally lower acceptance among households with higher baseline bouillon consumption.

Qualitative findings provided contextual explanation for the quantitative results. Focus group discussions identified perceived health benefits, favourable taste, cultural compatibility, and cost savings as key facilitators of cube use, consistent with the uniformly high acceptance observed across time points and subgroups. Initial concerns, including myths related to infertility and changes in food colour during cooking, helped explain the lower early perception observed among household heads, a group that was predominantly male in the study population. In contrast, wives and mothers, who were typically the primary cooks, reported greater familiarity with and trust in bouillon use, supporting their higher perception at the early intervention time point. Overall, the qualitative findings clarify how cooking roles, culturally embedded beliefs, and economic considerations shaped changes in perception over time while sustaining high acceptance.

The increase in perception and the stability of acceptance were similar between fortified and control cubes, consistent with the trial’s design and prior sensory evidence. The fortified cubes were deliberately formulated to be sensorily indistinguishable from standard cubes, and the micronutrient premix was not expected to alter taste, aroma, or colour, as demonstrated in earlier sensory evaluations conducted in Ghana [76]. In this context, perceived health improvements reported by participants may reflect household experiences and interpretations formed through routine use, rather than responses to sensory differences between fortified and control cubes. These findings are consistent with the principle underlying the mandatory fortification of staple foods and condiments, which aims to improve micronutrient intake without altering habitual consumption patterns. By maintaining sensory equivalence, fortified bouillon cubes can be integrated into existing commercial distribution channels with minimal need for consumer retraining or additional behaviour change messaging, thereby enhancing scalability and sustainability.

The study’s findings also emphasise the influence of sociodemographic and household roles. Higher perception among wives and stronger acceptance among farmers may reflect their central role in meal preparation and food planning. As NIHMs frequently serve as primary food decision-makers, their attitudes influence household engagement with the cubes and may shape intra-household reach and adherence to fortified foods. These findings suggest that bouillon fortification programmes may benefit from communication strategies that engage household members responsible for food preparation and purchasing decisions. These dynamics emphasise the need for future research to measure household decision processes directly.

The public health implications of fortified bouillon cubes remain substantial. Given their ubiquity in Ghanaian cooking [17], bouillon cubes are a culturally embedded, practical means for micronutrient delivery. However, they also contribute to sodium intake, raising considerations for hypertension and cardiovascular risk [77–79]. Although sodium-related concerns were rarely mentioned in qualitative interviews, particularly in relation to hypertension, these did not appear to influence cube use during the intervention. Nonetheless, national sodium monitoring programmes with particular focus on sources of dietary sodium remain essential as fortification programmes expand.

A ceiling effect in acceptance suggests that more sensitive measurement tools may be required to detect marginal behavioural changes [80,81]. This limitation may reduce the ability to detect subtle differences associated with messaging, packaging, or promotional innovations. More granular behavioural assessments may therefore be warranted in future implementation research.

From a policy standpoint, fortified bouillon cubes represent a promising platform for national fortification strategies due to their widespread use, low unit cost. Lessons from resistance to other fortification media (e.g., maize, rice) emphasise the advantage of integrating fortification into foods that already have strong cultural acceptance [29,82,83]. Effective scale-up may require complementary strategies such as subsidies, incentives for producers, and public–private partnerships, alongside sodium monitoring and public messaging on non-communicable disease risks [12,84]. Future research should incorporate biomarkers (e.g., haemoglobin, iodine status), explore potential ceiling effects, and refine behavioural measurement tools. Replication across diverse sociocultural contexts will help establish generalizability and optimize public health impact.

### Strengths and Limitations

This study has several strengths. The mixed-methods design, combining repeated quantitative measures within a randomised controlled trial and complementary qualitative inquiry, strengthened the depth and interpretability of the findings. To our knowledge, this is the first study to track perception and acceptance trajectories among non-index household members within a household-level fortification intervention. The composite perception and acceptance scores were derived using factor-analytic methods and demonstrated good internal consistency and confirmatory model fit, reflecting sound measurement properties. The use of Bayesian linear mixed-effects modelling enabled precise estimation of temporal changes while accounting for individual- and community-level variability.

However, some limitations should be acknowledged. The longitudinal analysis relied on complete cases (n = 676), reflecting the design of the study, in which household members from lactating-only households were not eligible for repeated measures of perception and acceptance because perception and acceptance were only assessed in households followed for the full nine-month intervention. These participants nevertheless contributed repeated data for other trial outcomes collected during the three-month follow-up. Among households eligible for follow-up, baseline characteristics were similar between completers and non-completers, reducing concerns about attrition bias, although the possibility of residual bias cannot be completely excluded. Missingness across covariates was low (<3%), and retention remained within the parent trial’s thresholds for sensitivity analyses, supporting the appropriateness of the complete-case approach. Behavioural outcomes were self-reported and may be subject to social desirability or recall bias; however, triangulation with qualitative data helped to mitigate this concern. Generalisability is likely greatest in settings with similar culinary practices, cultural preferences, and high baseline bouillon use; behavioural responses may differ in contexts where bouillon consumption is less common or where products are purchased commercially. Because bouillon cubes were provided free of charge, this sub study could not assess affordability or willingness to pay, limiting inference about purchasing behaviour under real-world market conditions. Finally, biochemical assessments were not collected among non-index household members, constraining the ability to directly link behavioural outcomes with biological markers.

## Conclusion

Fortified bouillon cubes were highly accepted and well-integrated into household cooking practices at the outset of the study. Perception among non-index household members improved over time, especially among groups initially less favourable, while acceptance remained consistently high throughout the intervention period. The findings indicate that fortified bouillon cubes can serve as a culturally compatible and operationally feasible strategy for improving micronutrient intake without requiring behavioural retraining or altering sensory properties. With routine monitoring of programme coverage, micronutrient safety, and sodium exposure, bouillon cube fortification holds substantial potential to strengthen nutrition programming across Ghana and West Africa.

## Supporting information

S1 File **S1 Table.** Standardised factor loadings for the final two-factor confirmatory factor analysis model of perception and acceptance of study-supplied bouillon cubes among non-index household members. Note: This table shows the standardised factor loadings from the final two-factor confirmatory factor analysis model used to derive the perception and acceptance composite scores. The final two-factor model comprises 8 items for perception and 10 items for acceptance. Items with factor loadings ≥ 0.40 were retained in the final model. Negatively worded items (Q6, Q26, and Q27) were reverse coded before analysis. **S2 Table.** Baseline comparison of completers and non-completers at follow-up. This table summarizes demographic and household characteristics at baseline for participants who completed both time points and those who did not. **S3 Table.** Sensitivity analyses of individual- and household-level factors associated with perception (panel a) and acceptance (panel b) of study-supplied bouillon cubes among non-index household members. These analyses assess the robustness of the Bayesian mixed-effects model findings to alternative prior specifications. **S4 Table.** Intercoder reliability scores (ICR) calculated as Cohen’s Kappa and percentage agreement across six double-coded transcripts. This table summarizes coding consistency metrics for qualitative analysis, based on independent coding of six focus group discussion transcripts by two researchers. **S5 File.** Trial protocol (version 4, August 29, 2022). This protocol describes trial design, randomisation, intervention procedures, and data collection methods. **S6 Table.** Background characteristics of focus group discussion participants (n = 157). This table summarizes demographic and socioeconomic characteristics of qualitative participants. **S7 File.** Thematic analysis of 24 focus group discussions examining perceptions and acceptance of study-supplied bouillon cubes. This file presents the full qualitative analytic outputs, including the final thematic framework, sub-themes, and representative participant quotations. It also includes supplementary outputs from sentiment analysis and subgroup analysis used to enrich and triangulate the qualitative findings. Each supplementary file provides detailed support for the quantitative and qualitative findings presented in the manuscript.
(ZIP)

S1 Check list CONSORT 2025 checklist. (DOCX)
