# Supplementary material for: Perception and acceptance of micronutrient-Fortified Bouillon among Non-Index Household Members: A longitudinal sub-study nested within a randomized trial in Northern Ghana
Source: PLoS One. 2026 Apr 3;21(4):e0345106. doi: 10.1371/journal.pone.0345106 (PMC13048496; doi:10.1371/journal.pone.0345106)
Supplement: S1 File — S1 Table. Standardised factor loadings for the final two-factor confirmatory factor analysis model of perception and acceptance of study-supplied bouillon cubes among non-index household members. Note: This table shows the standardised factor loadings from the final two-factor confirmatory factor analysis model used to derive the perception and acceptance composite scores. The final two-factor model comprises 8 items for perception and 10 items for acceptance. Items with factor loadings ≥ 0.40 were retained in the final model. Negatively worded items (Q6, Q26, and Q27) were reverse coded before analysis. S2 Table. Baseline comparison of completers and non-completers at follow-up. This table summarizes demographic and household characteristics at baseline for participants who completed both time points and those who did not. S3 Table. Sensitivity analyses of individual- and household-level factors associated with perception (panel a) and acceptance (panel b) of study-supplied bouillon cubes among non-index household members. These analyses assess the robustness of the Bayesian mixed-effects model findings to alternative prior specifications. S4 Table. Intercoder reliability scores (ICR) calculated as Cohen’s Kappa and percentage agreement across six double-coded transcripts. This table summarizes coding consistency metrics for qualitative analysis, based on independent coding of six focus group discussion transcripts by two researchers. S5 File. Trial protocol (version 4, August 29, 2022). This protocol describes trial design, randomisation, intervention procedures, and data collection methods. S6 Table. Background characteristics of focus group discussion participants (n = 157). This table summarizes demographic and socioeconomic characteristics of qualitative participants. S7 File. Thematic analysis of 24 focus group discussions examining perceptions and acceptance of study-supplied bouillon cubes. This file presents the full qualitative analytic outputs, including [file pone.0345106.s001.zip › supplementary material_Plos one/S3_Table.docx.docx]

**S3 Table**. Sensitivity analyses of individual- and household-level factors associated with perception (panel a) and acceptance (panel b) of study-supplied bouillon cubes among non-index household members

| **(a) Perception**  **Sensitivity analysis of individual and household factors associated with perception of study-supplied bouillon cubes among non-index household members at early (T1) and late (T2) intervention timepoints in the randomized trial** | | | |
| --- | --- | --- | --- |
| **Variable** | **Categorical Levels** | **Posterior Mean (β)** | **95% Credible Interval (CrI)** |
| **Individual-level factors** | | | |
| Occupation | Farming | ***Ref.*** |  |
|  | Homemaker | -0.12 | -0.31, 0.08 |
|  | Small Business Owners | -0.11 | -0.28, 0.05 |
|  | Formal Employees (Gov't/Private) | -0.35 | -0.60, -0.10* |
| Participant Relationship with household heads (HH) | HH | ***Ref.*** |  |
|  | Wives | 0.38 | 0.17, 0.60* |
|  | Sons/Daughters | 0.25 | 0.01, 0.49* |
|  | In-laws/Siblings | 0.30 | 0.07, 0.52* |
|  | Parents | 0.35 | 0.11, 0.58* |
| Sex | Female | ***Ref.*** |  |
|  | Male | -0.01 | -0.20, 0.19 |
| Household Cooking Role | Never Cooks | ***Ref.*** |  |
|  | Primary Cooks | 0.06 | -0.14, 0.26 |
|  | Secondary/Occasional Cooks | 0.08 | -0.10, 0.26 |
| **Household-level factors** | | | |
| Strata | Rural | ***Ref.*** |  |
|  | Urban | -0.04 | -0.32, 0.24 |
| District | Kumbungu | ***Ref.*** |  |
|  | Tolon | 0.08 | -0.21, 0.37 |
| Sex of HH | Female | ***Ref.*** |  |
|  | Male | 0.18 | -0.03, 0.39 |
| Socioeconomic Index | Continuous | -0.01 | -0.05, 0.04 |
| Baseline Household Bouillon Consumption (g/capital/day) | continuous | -0.04 | -0.08, 0.01 |
| **Timepoint and interaction effect** | | | |
| Interaction: Late × District | Kumbungu | ***Ref.*** |  |
|  | Tolon | -0.25 | -0.42, -0.08* |
| Interaction: Late × Occupation | Farming | ***Ref.*** |  |
|  | Homemaker | 0.12 | -0.15, 0.38 |
|  | Small Business Owners | 0.26 | 0.05, 0.48* |
|  | Formal Employees | 0.64 | 0.31, 0.98* |
| Interaction: Late × Relationship with HH | Household Head | ***Ref.*** |  |
|  | Wife | -0.62 | -0.84, -0.39* |
|  | Sons/Daughters | -0.28 | -0.60, 0.05 |
|  | Parents | -0.38 | -0.67, -0.09* |
|  | In-laws/Siblings | -0.52 | -0.82, -0.20* |
| Random Effect (Community level) | SD (Intercept) | 0.38 | 0.28, 0.52 |
| Random Effect (Participant level) | SD (Intercept) | 0.03 | 0.00, 0.08 |
| **Note:** Study-supplied bouillon cubes included either multiple micronutrient-fortified cubes (containing vitamins A and B12, folic acid, iron, zinc, and iodine) or control cubes fortified with iodine only. T1 represents the early intervention timepoint, with data collected during the second month of household exposure to the study bouillon cubes following an initial one-month adaptation period. T2 represents the late intervention timepoint, with data collected during the final two months of the nine-month intervention. Posterior means and 95% credible intervals (CrIs) were obtained from Bayesian linear mixed-effects models estimated using Hamiltonian Monte Carlo sampling. A Normal(0, 1) prior was used for fixed effects, and a Student-t(3, 0, 1) prior was used for the intercept. A CrI that does not include zero denotes statistical significance (*). Adjustment variables included in the model but not listed as predictors were timepoint (T1 vs. T2) and intervention group (micronutrient-fortified cubes vs. control), which were retained to account for the trial design and repeated measures but were not predictors of interest. The perception composite score ranges from 1 to 5, with higher values indicating more favourable perceptions of the bouillon cubes. The socioeconomic index was derived from a composite household asset score. Baseline household bouillon consumption was calculated as total grams used per day divided by household size, resulting in grams per capita per day. Farmers served as the reference category for occupation because they represented most participants. The random intercepts accounted for participant-level and community-level variability.  **Abbreviations:** HH, household head; CrI, credible interval; SD, standard deviation. | | | |

| **(b) Acceptance**  **Sensitivity analysis of individual and household factors associated with acceptance of study-supplied bouillon cubes among non-index household members** | | | |
| --- | --- | --- | --- |
| **Variable** | **Categorical levels** | **Posterior Mean (β)** | **95% Credible Interval (CrI)** |
| Sex | Female | ***Ref.*** |  |
|  | Male | 0.02 | -0.03, 0.06 |
| Occupation | Farming | ***Ref.*** |  |
|  | Homemaker | 0.03 | -0.04, 0.09 |
|  | Small Business Owners | 0.02 | -0.04, 0.07 |
|  | Formal Employees | 0.01 | -0.07, 0.09 |
| Religion | Islam | ***Ref.*** |  |
|  | Christianity/Traditionist | 0.08 | -0.08, 0.23 |
| Strata | Rural | ***Ref.*** |  |
|  | Urban | 0.01 | -0.11, 0.14 |
| Household Food Insecurity | Food Secure | ***Ref.*** |  |
|  | Mildly Food Insecure | -0.06 | -0.12, -0.00* |
|  | Moderately Food Insecure | -0.01 | -0.06, 0.03 |
|  | Severely Food Insecure | 0.08 | 0.01, 0.16* |
| Baseline household bouillon consumption (g/capital/day) | Continuous | -0.02 | -0.04, -0.00* |
| Socioeconomic Index | Continuous | 0.01 | -0.01, 0.03 |
| Random Effect (Community-level) | SD (Intercept) | 0.16 | 0.12, 0.22 |
| Random Effect (Participant-level) | SD (Intercept) | 0.02 | 0.00, 0.04 |
| **Note**: Study-supplied bouillon cubes included either multiple micronutrient-fortified cubes (containing vitamins A and B12, folic acid, iron, zinc, and iodine) or control cubes fortified with iodine only. T1 represents the early intervention timepoint, with data collected during the second month of household exposure to the study bouillon cubes following an initial one-month period of use, and T2 represents the late intervention timepoint, with data collected during the final two months of the nine-month intervention. Posterior means and 95% credible intervals (CrIs) were obtained from Bayesian linear mixed-effects models estimated using Hamiltonian Monte Carlo sampling. A Normal(0, 1) prior was used for fixed effects, and a Student-t(3, 0, 1) prior was used for the intercept. A CrI that does not include zero denotes statistical significance (*). Adjustment variables included in the model but not listed as predictors were timepoint (T1 vs. T2) and intervention group (micronutrient-fortified cubes vs. control), which were retained to account for the trial design and repeated measures but were not predictors of interest. The acceptance composite score ranges from 1 to 5, with higher values indicating stronger acceptance of the bouillon cubes. The socioeconomic index was derived from a composite household asset score. Baseline household bouillon consumption was calculated as total grams used per day divided by household size, resulting in grams per capita per day. Farmers served as the reference category for occupation because they represented most participants. Random intercepts accounted for both participant-level and community-level variability.  **Abbreviations:** HH, household head; CrI, credible interval. | | | |
