# Supplementary material for: Perception and acceptance of micronutrient-Fortified Bouillon among Non-Index Household Members: A longitudinal sub-study nested within a randomized trial in Northern Ghana
Source: PLoS One. 2026 Apr 3;21(4):e0345106. doi: 10.1371/journal.pone.0345106 (PMC13048496; doi:10.1371/journal.pone.0345106)
Supplement: S1 File — S1 Table. Standardised factor loadings for the final two-factor confirmatory factor analysis model of perception and acceptance of study-supplied bouillon cubes among non-index household members. Note: This table shows the standardised factor loadings from the final two-factor confirmatory factor analysis model used to derive the perception and acceptance composite scores. The final two-factor model comprises 8 items for perception and 10 items for acceptance. Items with factor loadings ≥ 0.40 were retained in the final model. Negatively worded items (Q6, Q26, and Q27) were reverse coded before analysis. S2 Table. Baseline comparison of completers and non-completers at follow-up. This table summarizes demographic and household characteristics at baseline for participants who completed both time points and those who did not. S3 Table. Sensitivity analyses of individual- and household-level factors associated with perception (panel a) and acceptance (panel b) of study-supplied bouillon cubes among non-index household members. These analyses assess the robustness of the Bayesian mixed-effects model findings to alternative prior specifications. S4 Table. Intercoder reliability scores (ICR) calculated as Cohen’s Kappa and percentage agreement across six double-coded transcripts. This table summarizes coding consistency metrics for qualitative analysis, based on independent coding of six focus group discussion transcripts by two researchers. S5 File. Trial protocol (version 4, August 29, 2022). This protocol describes trial design, randomisation, intervention procedures, and data collection methods. S6 Table. Background characteristics of focus group discussion participants (n = 157). This table summarizes demographic and socioeconomic characteristics of qualitative participants. S7 File. Thematic analysis of 24 focus group discussions examining perceptions and acceptance of study-supplied bouillon cubes. This file presents the full qualitative analytic outputs, including [file pone.0345106.s001.zip › supplementary material_Plos one/S5_File.pdf.pdf]

Table of Contents

1. General Information 3
List of Abbreviations 4
2. Protocol Summary 6
3. Background and Rationale 7
4. Literature Review 8
5. Study goals and objectives 10
Primary and secondary outcomes 11
6. Methodology 16
Study design 16
Study site 20
Study participants 20
Inclusion and Exclusion Criteria 21
Fortified bouillon development 26
Study procedures 30
COVID-19 precautions 30
Overview 30
Recruitment 33
Biospecimen screening, for non-pregnant, non-lactating women of reproductive age 35
Run-in period 36
Baseline visit 37
Randomization and blinding 40
Intervention period 41
Endline visits 42
Data collection methods 43
Household information 43
Adherence 43
Morbidity 44
Anthropometry 44
Dietary intake 45
Blood sample collection and processing 46
Breast milk sample collection and processing 49
Urine collection and processing 50

Stool collection (children only) 50
Biological sample storage, transport, and analysis........ 51
7. Sample size........ 54
8. Data and safety monitoring 58
9. Data analysis 59
10. Training 59
11. Quality assurance........ 60
12. Expected outcomes of the study 60
13. Dissemination of results and publication policy 60
14. Duration of the project 60
15. Problems anticipated........ 61
16. Project Management 61
18. Data and/or Specimen Management and Confidentiality........ 62
19. Data and/or Specimen Banking 63
20. Provisions to Monitor the Data to Ensure the Safety of Subjects 64
21. Withdrawal of Participants 65
22. Risks to Participants........ 66
23. Potential benefits to participants 67
24. Sharing of results with participants 68
25. Prior approvals and funding information 68
26. Compensation for research-related injury 68
27. Economic burden to participants........ 68
28. Work Plan........ 70
29. Study Budget........ 71
30. References 72

1. General Information

Protocol Title: Effect of household use of multiple micronutrient-fortified bouillon on micronutrient status among women and children in two districts in the Northern Region of Ghana.

Date: 29 August 2022

Name and title of the investigator(s) who is (are) responsible for conducting the research, and the address

Seth Adu-Afarwuah, Ph. D.
Department of Nutrition and Food Science

Legon, Ghana

Reina Engle-Stone, Ph. D.
Institute for Global Nutrition

University of California, Davis

List of Abbreviations

AGP	-	alpha-1-acid glycoprotein
AE	-	Adverse Event
APP	-	Acute phase protein
BIS	-	Body iron stores
BMI	-	Body mass index
BP	-	Blood pressure
C19	-	COVID-19
CA/TSC	-	Citric acid/trisodium citrate
CoMIT	-	Condiment Micronutrient Innovation Trial
CMAM	-	Community Management of Acute Malnutrition
CRP	-	C-reactive protein
CSIRO	-	Commonwealth Scientific and Industrial Research Organization
DSMB	-	Data and safety monitoring board
DFE	-	Dietary folate equivalents
EDTA	-	Ethylenediaminetetraacetic acid
ERC	-	Ethical review committee
**YET**	**-**	**Early Years Toolbox**
FACT	-	Fortification Assessment Coverage Tool
FDA	-	Food and Drugs Authority
**FGD**	**-**	**Focus group discussion**
FFQ	-	Food frequency questionnaire
FePP	-	Ferric pyrophosphate
GHS	-	Ghana Health Service
GLSS	-	Ghana Living Standard Survey
Hb	-	Hemoglobin
HCG	-	Human chorionic gonadotropin
Hct	-	Haematocrit
HFIAS	-	Household Food Insecurity Access Scale
HH	-	Household
HOME	-	Home Observation Measurement of the Environment
HPLC	-	High performance liquid chromatography
HRP	-	Histidine-rich protein
HWISE	-	Household Water Insecurity Experiences
ICP-OES	-	Inductively coupled plasma - optical emission spectrometry
ID	-	Identification
IRB	-	Institutional Review Board
KAP	-	Knowledge Attitudes and Practices
LC/MS/MS	-	Liquid chromatography with tandem mass spectrometry
MDAT	-	Malawi Development Assessment Tool
MDD-W	-	Minimum Dietary Diversity- Women
MN	-	Micronutrients
MUAC	-	Mid-upper arm circumference
**OAA**	**-**	**Opinions, attitudes, and acceptance**

NI	-	Nutrition International
PI	-	Principal Investigator
PII	-	Personal identifying information
PPE	-	Personal protective equipment
pZn	-	Plasma zinc
RAE	-	Retinol Activity Equivalents
RBC	-	Red blood cell
RBP	-	Retinol binding protein
RDA	-	Recommended dietary allowance
RDT	-	Rapid diagnostic test
RID	-	Retinol isotope dilution
RISE	-	Research Institutes of Sweden
SAE	-	Serious Adverse Event
SAP	-	Statistical analysis plan
SES	-	Socioeconomic status
SOP	-	Standard operating procedure
STEPS	-	WHO STEPwise Approach to NCD Risk Factor Surveillance
sTfR	-	Soluble transferrin receptor
WHNRC	-	Western Human Nutrition Research Center
WRA	-	Women of Reproductive Age
UL	-	Upper level
US CDC	-	United States Centers for Disease Control and Prevention
ZPP	-	Zinc protoporphyrin

2. Protocol Summary

Background: Micronutrient (MN) deficiencies are severe and widespread in West Africa, particularly among young children and women of reproductive age. Bouillon is a promising food fortification vehicle because the product is centrally processed on large scale, consumed by most households in West African countries (even rural, poor households), and consumed by most members of the household in relatively constant amounts. However, several important research questions remain regarding whether the use of fortified bouillon would be feasible and effective for preventing or reducing micronutrient deficiencies in communities where such deficiencies are common. Specifically, no studies have assessed the impacts of multiple micronutrient-fortified bouillon on micronutrient status. The West Africa Condiment Micronutrient Innovation Trial (CoMIT) Project aims to address this gap, to inform future discussions around fortification of bouillon cubes and related products.

Objective: This study aims to assess the impacts of household use of multiple micronutrient-fortified bouillon cubes (containing iodine in addition to vitamin A, folic acid, vitamin B12, iron, and zinc), compared to control bouillon cubes fortified with iodine only, on:

a) Micronutrient status among women 15-49 years of age and children 2-5 years of age after 9 months of intervention
b) Hemoglobin concentrations among women 15-49 years of age and children 2-5 years of age after 9 months of intervention
c) Breast milk micronutrient among lactating women 4-18 months postpartum after 3 months of intervention

Methods: This randomized, controlled doubly-masked trial will be conducted in the Kumbungu and Tolon districts in the Northern Region of Ghana, where prior data indicate that deficiencies in the selected nutrients are common. Potential participants will be: 1) non-pregnant non-lactating women of reproductive age (15 - 49 years old), 2) children 2-5 years of age, and 3) non-pregnant lactating women 4-18 months postpartum. Eligible participants will be randomly assigned to receive household rations of one of two types of bouillon cubes:

(a) a multiple micronutrient-fortified bouillon cube containing vitamin A, folic acid, vitamin B12, iron, zinc, and iodine, or
(b) a control cube containing iodine only

Each participant's household will receive a specific amount of bouillon cube every 2 weeks, and households will be advised to prepare their meals as usual, using the study-provided cubes. The trial duration will be 9 months (38 weeks) for non-pregnant, non-lactating women and children 2-5 years of age, and 3 months (12 weeks) for lactating women. The primary outcomes will be changes from baseline to endline in concentrations of haemoglobin and biomarkers of micronutrient status. Secondary outcomes will include change in prevalence of anaemia and micronutrient deficiency; dietary intake of bouillon and micronutrients; inflammation, malaria, and morbidity symptoms; and children's anthropometric measures and child development.

Expected outcomes: This study will provide the first evidence regarding the impacts of household use of multiple micronutrient-fortified bouillon cubes on the micronutrient status of those commonly at risk for micronutrient deficiencies (e.g., women and young children). This information will help to inform discussions about bouillon fortification with micronutrients within Ghana and in West Africa.

3. Background and Rationale

Micronutrient (MN) deficiencies are severe and widespread in West Africa, particularly among young children, pregnant/lactating women, and women of reproductive age. These deficiencies have developmental and physical consequences, including increased risk of mortality, which, in turn, have economic consequences. While various programs to address MN deficiencies have been scaled up, including industrial fortification of cooking oil and wheat flour with micronutrients, it is unlikely that a single such program will eliminate all MN deficiencies, since delivery platforms may provide only a subset of nutrients or reach only a subset of the deficient population. Given current technologies and market structures for fortifiable products, a combination of strategies is generally needed to completely address MN deficiencies across different socioeconomic settings, and these strategies can vary over space and over time. Due to the (always) limited funds available for MN intervention programs, governments must seek cost-effective strategies for addressing MN deficiencies, which may be achieved by working in concert with private sector producers and providers of fortifiable products.

The use of bouillon could have great potential for addressing MN deficiencies in Ghana and West Africa. Bouillon is an ideal fortification vehicle because the product is centrally processed on large scale, consumed by most households in West African countries (even rural, poor households), and consumed by most members of the household in relatively constant amounts. However, important questions about large-scale use of bouillon to address micronutrient deficiencies need to be addressed. Technical questions include how many different MN, and how much of each MN, can be added simultaneously to bouillon cubes. Adding different MN in different amounts will also certainly affect bouillon production costs and perhaps retail prices, and might also affect consumer acceptability of/demand for these products. Site- and population-specific questions include the extent to which multi-fortified bouillon cubes can address inadequate intake of key MN, especially among children, women of reproductive age, and other groups at risk of micronutrient deficiencies, and whether the secondary data-based modelled contribution of fortified bouillon to MN intakes will translate into real improvements in nutritional and health status. Additionally, strategies must be developed to avoid causing unacceptably high levels of consumption of selected MN or sodium, especially in situations involving multiple food/product fortification programs. In many low-income country settings where industrial fortification of other foods is already in place, a key policy question is whether there are additional health benefits to be gained from fortifying a new food vehicle, and what are the safety considerations to be made regarding the new fortified food. Other gaps in policy include the lack of manufacturing standards for bouillon in most countries, and the unavailability of technical specification and/or standards to guide its fortification with micronutrients, or clear and evidence-based processes for identifying these guidelines. Economic questions remain regarding the increased production costs of multi-fortified cubes and the effects of any product-cost increases on retail prices and hence on cube consumption patterns, especially among the very poor, and the extent to which bouillon cube fortification represents a cost-effective addition to the current portfolio of micronutrient intervention programs. Finally, management questions include the design and implementation of effective monitoring and evaluation activities for fortified bouillon.

The University of California, Davis and University of Ghana, Legon have received research funding for the Condiment Micronutrient Innovation Trial (CoMIT) Project, which aims to address the gaps identified above via: 1) modelling of fortification levels for bouillon that will minimize both inadequate and excessive intakes, 2) field-based testing of multi-MN fortified bouillon for its effects on adequacy of intake and biomarkers of micronutrient status among

selected individuals within households; and 3) the development and use of a micronutrient intervention program modelling (MINIMOD-SD, where "SD" indicates the use of secondary data, primarily) tool to assess the effective coverage (i.e., effect of the intervention on prevalence of dietary inadequacy) and cost-effectiveness of this new MN delivery vehicle vis à-vis other (traditional) vehicles and MN programs at national and regional levels.

Pilot research to measure micronutrient status among women and children and to assess dietary intake patterns and perceptions of bouillon at the proposed site for the planned trial (objective 2 above) were approved separately by the Ghana Health Service **Ethical Review Committee** (GHS-ERC 012/07/20) and the UC Davis **Institutional Review Board (**IRB #1536100). In addition, a study to assess the acceptability of a multiple micronutrient-fortified bouillon cube **was approved** by the Ghana Health Service ERC (GHS-ERC 017/12/20) and the UC Davis IRB (#1689885). The current protocol describes the procedures for a proposed randomized, controlled trial to assess the impact of multiple micronutrient-fortified bouillon cube on micronutrient status of women and young children in northern Ghana.

4. Literature Review

i. Prevalence of micronutrient deficiencies in northern Ghana

Micronutrient deficiencies remain common in Ghana, particularly among women and children, despite various decades-long intervention programs to address them. In northern Ghana, the problem of micronutrient deficiency is even more severe when compared with the southern part of the country. A recent national survey (Univ Ghana/GroundWork/Univ Wisconsin-Madison/KEMRI/UNICEF, 2017) showed that among preschool children (6-59 mo of age), the prevalence of iron and vitamin A deficiency (as well as anaemia) is markedly higher in the northern part (belt) of the country (i.e. Upper West, Upper East, and Northern regions as at the time of the survey) compared with the middle and southern belts. The percentage of preschool children who had iron deficiency (serum ferritin <12 μg/L adjusted for inflammation) was 40% for the northern belt compared with 18% for the middle, and 13% for the southern belts. Likewise, the percentage of children with vitamin A deficiency (retinol binding protein <0.70 μmol/L, after adjustment for inflammation) was 31%, 18% and 17% for the northern, middle, and southern belts, respectively. Among non-pregnant women (15-49 y), the prevalence of iron deficiency (serum ferritin <15 μg/L after adjustment for inflammation) followed the same pattern as that for pre-school children, with the northern belt (22%) being worst off, compared with the middle (11%) and southern (14%) belts.

Much of the micronutrient deficiencies in Ghana, particularly northern Ghana, may be due to low intake of micronutrient-rich foods (e.g., iron, vitamin A, zinc) (Bailey, West, & Black, 2015) or to low micronutrient concentrations in the environment from which staple foods are obtained (e.g., iodine, selenium) (Klassen-Wigger et al., 2018). The Ghana 2017 survey (Univ Ghana/GroundWork/Univ Wisconsin-Madison/KEMRI/UNICEF, 2017) showed that within the northern belt of the country, the areas surrounding Tamale, which includes the districts of Tolon and Kumbungu, are some of the "pockets" within the belt where the prevalence of iron deficiency and vitamin A deficiency among preschool children are highest, compared with elsewhere in the northern belt. The same surrounding areas around Tamale were noted for anaemia and iron deficiency rates of >40% among non-pregnant women.

While micronutrient deficiencies remain common in northern Ghana, the consumption of fortified staple food products in the area appears low. In the 2017 survey (Univ

Ghana/GroundWork/Univ Wisconsin-Madison/KEMRI/UNICEF, 2017), only 36% of the oil samples collected from the area were adequately fortified with vitamin A (≥10 mg/kg vitamin A), and only 6% of wheat flour samples collected had adequate iron concentrations (≥ 58.5 ppm) as mandated by Ghana's food fortification standards. More aggressive efforts are thus required to address the high prevalence of micronutrient deficiencies in northern Ghana and other similar areas in West Africa.

ii. Past and ongoing interventions to address micronutrient deficiencies in Ghana and proposed recommendations

Many nutrition-sensitive and nutrition-specific intervention programs have been implemented in Ghana, either in the past or still on-going, as part of efforts to reduce micronutrient deficiencies (GoG, 2013). Notable among these are:

- Iron and folic acid supplementation for pregnant women during antenatal contacts and adolescent girls in school
- High dose vitamin A supplementation for women within 8 weeks after giving birth and to children 6-59 mo of age
- Flour and vegetable oil fortification for the general population
- Nutrition and malaria control for pregnant and lactating women and children 0–59 months during health contacts in selected districts
- Community-based management of acute malnutrition (CMAM) for children under 5 years with severe acute malnutrition
- Supplementary feeding and nutrition education programme for pregnant and lactating women, and children under 5 years with moderate malnutrition in food insecure households in northern Ghana

Following the Ghana Micronutrient Survey, some of the proposed recommendations for reducing micronutrient deficiencies (Univ Ghana/GroundWork/Univ Wisconsin-Madison/KEMRI/UNICEF, 2017) were:

- Promotion of foods (fortified or unfortified) rich in iron and vitamin A for children.
- Promotion of the consumption of iron-rich foods and iron supplements for women.
- Promotion of the consumption of folic acid-containing supplements among women of reproductive age.
- Monitoring of the implementation of Ghana's wheat flour and vegetable oil fortification programs

iii. Use of fortified bouillon cubes

One strategy to reduce micronutrient deficiencies in Ghana could be to fortify regularly consumed products such as bouillon (or Maggi) cubes with relevant micronutrients. Bouillon cubes are particularly promising given that they are regularly consumed across different socioeconomic brackets throughout the country. In several sub-Saharan African countries including Burkina Faso, Cameroon, Niger, and Senegal, it was discovered that 79% (Burkina Faso) to 99% (Senegal) of women reportedly consumed bouillon cubes in the past 7 days, compared with at least 48% who reported consuming wheat flour, or at least 44% who reportedly consumed vegetable oil (Hess et al., 2013). It is estimated that in 2013, the Nestle company sold 90 billion servings of Maggi bouillon cubes or tablets, of which 40 billion were fortified with a low-level of iron (0.6 mg/g) (Klassen-Wigger et al., 2018). Preliminary analyses of household

data in Ghana (Kumordzie et al., 2019) suggested that 72% of households purchased bouillon products in the prior 18 days.

In 2014, the World Health Organization, in collaboration with the Micronutrient Initiative (now Nutrition International, or NI) and the New York Academy of Sciences, convened a consultation on the fortification of condiments and seasonings with vitamins and minerals. The consultation report (Garcia-Casal et al., 2016) highlighted research needs identified during workshop breakout sessions, such as identifying the appropriate levels of micronutrient fortificants, data on stable and bioavailable fortificants, and on costs and coherence with other micronutrient programs.

As far back as nearly 2 decades ago, micronutrient-fortified condiments such as curry powder (Ballot, MacPhail, Bothwell, Gillooly, & Mayet, 1989) and soy sauce (Chen et al., 2005) were known to be efficacious in increasing micronutrient intakes and reducing micronutrient deficiencies in several countries (Mannar & Gallego, 2002; Theary et al., 2013). Although the daily intakes of iron delivered via fortification of products such as curry powder, soy sauce or fish sauce may be higher than what would normally be achieved through consumption of iron-fortified bouillon cubes, even a low iron concentration of 5 mg/d in soy sauce was found to improve iron status and reduce anemia in Chinese teenagers in only 3 months (Huo et al., 2002). In Central and West Africa, Maggi cubes have been fortified with iodized salt since the 1990s (Klassen-Wigger et al., 2018), and the fortification of Maggi was extended to include iron (0.6 mg/g), because of the high prevalence of micronutrient deficiencies in the region. A study in Senegal (Spohrer et al., 2015) found that iodine fortification of bouillon could potentially contribute substantially to iodine intake, by providing between 7%-115% of the recommended daily intake, depending on the iodine content of the cubes. Other modelling studies have found that bouillon cube fortified with vitamin A could reduce the prevalence of inadequate vitamin A intakes without leading to an increase in excessive intakes among women or children in Cameroon (Engle-Stone, Nankap, Ndjebayi, & Brown, 2014) and would complement other intervention programs in reducing inadequate intake of vitamin A (Vosti et al. 2019). Finally, a study of schoolchildren 6-13 years of age in northern Ghana (Abizari, Dold, Kupka, & Zimmermann, 2017) found that around two-thirds (2/3) of dietary iodine is obtained from iodine-fortified bouillon cubes.

The foregoing reviews suggest that the use of bouillon cubes in combination with other ongoing measures could contribute to the prevention of micronutrient deficiencies in Ghana. However, the levels of nutrients such as iron that are included in commercial bouillon products may not be sufficient to meet public health needs (Luo et al., 2016). Moreover, no commercial bouillon cubes have been released that address deficiencies in other key nutrients such as zinc, folate, and vitamin B-12. Therefore, additional research is needed to explore novel bouillon formulations that will increase the potential impact on micronutrient deficiencies.

5. Study goals and objectives

This study aims to assess the effects of household use of multiple micronutrient-fortified bouillon cubes (containing iodine in addition to vitamin A, folic acid, vitamin B12, iron, and zinc), compared to control bouillon cubes (fortified with iodine only), on:

a) Micronutrient status among women 15-49 years of age and children 2-5 years of age after 9 months
b) Hemoglobin concentrations among women 15-49 years of age and children 2-5 years of age after 9 months

c) Breast milk micronutrient concentrations among lactating women 4-18 months postpartum after 3 months

We hypothesize that micronutrient status and haemoglobin concentrations will increase after 9 months among women and children who receive the multiple micronutrient-fortified bouillon compared to those who receive the control bouillon, and that breast milk micronutrient concentrations will increase after 3 months among women who receive the multiple micronutrient-fortified bouillon compared to those who receive the control bouillon.

Secondary objectives include evaluation of the effect of the intervention, compared to control, on anemia, prevalence of micronutrient deficiencies, dietary nutrient intakes and morbidity among women and children, and growth and development among children. See Tables 1 and 2 below for a detailed list of primary and secondary outcomes.

Primary and secondary outcomes

Table 1. Primary outcomes[1]

**Target group**	**Primary outcomes**
Non-pregnant, non-lactating women of reproductive age (WRA)	• Change in micronutrient status after 38 weeks:<br>o Total body vitamin A stores and liver vitamin A concentration (measured by retinol isotope dilution)<br>o **Plasma** ferritin and soluble transferrin receptor (sTfR) concentrations, and calculated body iron stores (BIS)<br>o **Plasma** zinc concentration<br>o Erythrocyte folate concentrations<br>o **Plasma** vitamin B12 concentration<br>• Change in hemoglobin (Hb) concentration after 38 weeks
Children 2-5 y	• Change in micronutrient status after 38 weeks:<br>o **Plasma** retinol binding protein **(**RBP) concentrations<br>o **Plasma** ferritin and soluble transferrin receptor (sTfR) concentrations, and calculated body iron stores (BIS)<br>o **Plasma** zinc concentration<br>o **Plasma** folate concentration<br>o **Plasma** vitamin B12 concentration<br>• Change in hemoglobin concentration after 38 weeks
Non-pregnant, lactating women	• Change in breast milk vitamin A concentration after 12 weeks (controlling for milk fat content)<br>• Change in breast milk vitamin B12 concentration after 12 weeks

[1] **Primary outcomes are measured at baseline and endline, and there are no pre-planned interim analyses of intervention efficacy.**

Table 2. Secondary outcomes

Target group	Secondary outcomes
Household	• Household consumption of bouillon (total and per capita)<br>• Adherence (consumption of study-provided bouillon, expressed as % of total household bouillon consumption)
Non-pregnant, non-lactating women of reproductive age	• Change in serum **or plasma** retinol and RBP concentrations after 38 weeks<br>• Change in serum folate concentrations after 38 weeks<br>• Change in urinary iodine concentrations after 38 weeks<br>• Urinary sodium concentrations, and sodium:potassium ratio<br>• Change in prevalence of micronutrient deficiency after 38 weeks<br>• Change in prevalence of anaemia after 38 weeks<br>• Inflammation (concentrations of C-reactive protein (CRP) and alpha-1-acid glycoprotein (AGP) and prevalence of elevated values, and other markers including amyloid A, monocyte chemoattractant protein-1 (MCP-1), IL-6, IL-10, IL1beta, and adiponectin) at baseline and endline<br>• Current or recent malaria (by rapid diagnostic test; RDT) at baseline and endline<br>• Morbidity (cumulative days of symptoms over study duration)<br>• Dietary intake of bouillon, salt, fortifiable foods, energy, macronutrients, and micronutrients<br>• Blood pressure and hypertension prevalence
Children 2-5 y	• Change in urinary iodine concentrations after 38 weeks<br>• Change in prevalence of micronutrient deficiency after 38 weeks<br>• Change in prevalence of anaemia after 38 weeks<br>• Change in **plasma** retinol concentrations after 38 weeks<br>• Inflammation (concentrations of C-reactive protein and alpha-a-acid glycoprotein and prevalence of elevated values) at baseline and endline<br>• Current or recent malaria (by RDT) at baseline and endline<br>• Faecal calprotectin concentrations at baseline and endline<br>• **Faecal microbiota at baseline and endline**<br>• Morbidity (cumulative days of symptoms over study duration)<br>• Dietary intake of bouillon, salt, fortifiable foods, energy, macronutrients, and micronutrients<br>• Anthropometric measurements (change in height-for-age and weight-for-height Z-scores; prevalence of stunting and wasting)<br>• Change in Malawi Development Assessment Tool (MDAT) scores in 4 domains (gross motor, fine motor, language, social) after 38 weeks<br>• **Change in Early Years Toolbox (EYT) scores after 38 weeks**
Non-pregnant, lactating women	• Change in urinary iodine concentrations after 12 weeks<br>• Change in prevalence of low milk nutrient concentrations after 12 weeks<br>• Dietary intake of bouillon, salt, fortifiable foods, energy, macronutrients, and micronutrients

**Target group**	**Secondary outcomes**
Household	• Household consumption of bouillon (total and per capita)<br>• Adherence (consumption of study-provided bouillon, expressed as % of total household bouillon consumption)
	• Morbidity (cumulative days of symptoms over study duration)

Box 1. Specification and definitions of haemoglobin and micronutrient biomarker variables for children 2-5 years of age[1]

**Outcome variables**	**Definitions of dichotomous outcome variables**
Hemoglobin (Hb; g/L)	
Anemia	Hb < 110 g/L (WHO, 2011)
Mild anemia	Hb 100-109 g/L
Moderate to severe anemia	Hb 70-99 g/L
Severe anemia	Hb < 70 g/L
**Plasma** ferritin (µg/L)	
Iron deficiency (low **plasma** ferritin concentration)	**Plasma** ferritin < 12 µg/L (WHO, 2011)
**Plasma** soluble transferrin receptor concentration (sTfR; mg/L)	
Elevated **plasma** sTfR concentration	**Plasma** sTfR > 8.3 mg/L (Erhardt, Estes, Pfeiffer, Biesalski, & Craft, 2004)
Any iron deficiency	**Plasma** ferritin <12 µg/L and/or sTfR > 8.3 mg/L
Iron deficiency anemia	(**plasma** ferritin <12 µg/L and/or sTfR > 8.3 mg/L) and Hb < 110 g/L (World Health Organization)
Low ferritin and anemia	**Plasma** ferritin <12 µg/L and Hb < 110 g/L (World Health Organization)
Body iron stores (BIS; mg/kg)	
Low BIS	< 0 mg/kg (Cook, Flowers, & Skikne, 2003)
**Plasma** zinc concentration (µg/dL)	
Low **plasma** zinc concentration	**Plasma** zinc < 65 µg/dL (Brown et al., 2004)
**Plasma** retinol concentration (µmol/L)	
Low retinol	**Plasma** retinol < 0.70 µmol/L (WHO, 2011)
Marginal retinol	**Plasma** retinol < 1.05 µmol/L
**Plasma** retinol binding protein concentration (RBP; µmol/L)[2]	
Low RBP	Study-specific cut-off to be determined
Marginal RBP	Study-specific cut-off to be determined
**Plasma** folate concentrations (nmol/L)	
Low folate	< 10 nmol/L (L. B. Bailey et al., 2015)
Low folate and anemia	**Plasma**folate < 10nmol/L and anemia (Hb < 120 g/L)
**Plasma** vitamin B12 concentrations (pmol/L)	
Low vitamin B12	< 148 pmol/L (Allen et al., 2018)
Depleted vitamin B12	< 221 pmol/L
Low B12 and anemia	Vitamin B12 < 148 pmol/L and anemia (Hb < 120 g/L)

[1]Hb, hemoglobin; BIS, body iron stores; RBP, retinol binding protein; sTfR, soluble transferrin receptor. Procedures for adjusting micronutrient biomarkers for inflammation and methodological factors, as appropriate, will be described in the Statistical Analysis Plan. [2]There is currently no internationally established cut-off for RBP that reflects a **plasma** retinol concentration of < 0.70 µmol/L or < 1.05 µmol/L, and it has been recommended that trials determine the relationship between retinol and RBP in a sub-sample of the trial population to establish study-specific RBP cut-offs.

Box 2. Specification and definitions of haemoglobin and micronutrient biomarkers variables for non-pregnant, non-lactating women of reproductive age[1]

**Outcome variables**	**Definitions of dichotomous outcome variables**
Hemoglobin (Hb; g/L)	
Anemia	Hb < 120 g/L (WHO, 2011)
Mild anemia	Hb 110-119 g/L
Moderate to severe anemia	Hb 80-109 g/L
Severe anemia	Hb < 80 g/L
Hematocrit (Hct; L/L)	
Anemia	Hct < 0.36 L/L (World Health Organization)
**Plasma** ferritin (µg/L)	
Iron deficiency (low **plasma** ferritin concentration)	**Plasma** ferritin < 15 µg/L (WHO, 2011)
**Plasma** soluble transferrin receptor concentration (sTfR; mg/L)	
Elevated **plasma** sTfR concentration	**Plasma** sTfR > 8.3 mg/L (Erhardt et al., 2004)
Any iron deficiency	**Plasma** ferritin <15 µg/L and/or sTfR > 8.3 mg/L
Iron deficiency anemia	(**Plasma** ferritin <15 µg/L and/or sTfR > 8.3 mg/L) and (Hb < 120 g/L or Hct < 0.36 L/L)
Low ferritin and anemia	**Plasma** ferritin <15 µg/L and (Hb < 120 g/L or Hct < 0.36 L/L)
Body iron stores (BIS; mg/kg)	
Low BIS	BIS < 0 mg/kg (Cook et al., 2003)
**Plasma** zinc concentration (µg/dL)	
Low **plasma** zinc concentration	**Plasma** zinc < 70 µg/dL (fasting); < 66 µg/dL (non-fasting) (Brown et al., 2004)
Serum **(plasma)** retinol concentration (µmol/L)	
Low retinol	Serum **(plasma)** retinol < 0.70 µmol/L (WHO, 2011)
Marginal retinol	Serum **(plasma)** retinol < 1.05 µmol/L
**Plasma** retinol binding protein concentration (RBP; µmol/L)[2]	
Low RBP	Study-specific cut-off to be determined
Marginal RBP	Study-specific cut-off to be determined
Serum folate concentrations (nmol/L)	
Low serum folate	< 10 nmol/L (L. B. Bailey et al., 2015)
Erythrocyte (RBC) folate concentrations	
Low RBC folate	< 340 nmol/L (L. B. Bailey et al., 2015)
Low folate and anemia	RBC folate < 340 nmol/L or serum folate < 10nmol/L and anemia (Hb < 120 g/L)
**Plasma** vitamin B12 concentrations (pmol/L)	
Low vitamin B12	< 148 pmol/L (Allen et al., 2018)
Depleted vitamin B12	< 221 pmol/L
Low B12 and anemia	Vitamin B12 < 148 pmol/L and anemia (Hb < 120 g/L)

[1]Hb, hemoglobin; **Hct, haematocrit;** BIS, body iron stores; **RBC, red blood cell;** RBP, retinol binding protein; sTfR, soluble transferrin receptor. Procedures for adjusting micronutrient biomarkers for inflammation and methodological factors, as appropriate, will be described in the Statistical Analysis Plan. [2]There is currently no internationally established cut-off for RBP that reflects a **plasma** retinol concentration of < 0.70 µmol/L or < 1.05 µmol/L, and it has been recommended that trials determine the relationship between retinol and RBP in a sub-sample of the trial population to establish study-specific RBP cut-offs.

6. Methodology

Study design

The trial will follow a randomized, controlled doubly-masked design (see overview in Figure 1). Using a random walk method, we will conduct door-to-door recruitment and eligible participants will be enrolled from households in selected communities within the Kumbungu and Tolon districts. Women of reproductive age (WRA) will also undergo an additional screening visit for assessment of total body vitamin A using the retinol isotope dilution (RID) technique. Next, participants will **undergo a 3-week observation period (corresponding with the time frame between receipt of the vitamin A isotope dose and the baseline blood sample) for collection of data on typical bouillon purchasing, dietary intake, and morbidity.**

Subsequently, at baseline, eligible participants will be randomly assigned to receive household rations of one of two types of bouillon cubes:
(a) a multiple micronutrient-fortified bouillon cube containing vitamin A, folic acid, vitamin B12, iron, zinc, and iodine
(b) a control cube containing iodine only

Households will be advised to prepare their meals as usual, but substitute the bouillon provided by the study for the commercially available bouillon that they typically purchase. Households will be visited every 2 weeks, for delivery of study cubes, monitoring of adherence and recording of morbidity symptoms. The trial duration will be 9 months (38 weeks) for non-pregnant, non-lactating women and children 2-5 years of age, and 3 months (12 weeks) for lactating women. A subset of the primary outcomes will be measured once at baseline and twice at endline (i.e., after 35 and 38 weeks for blood biomarkers and after 11 and 12 weeks for breast milk biomarkers) to increase statistical power (see **Table 5**).

Figure 1. Overview of study procedures. For lactating women, the schedule is similar except that the total intervention period is 3 months and the two endline visits will be conducted at 11 weeks and 12 weeks. For participants in households in which there is no potentially eligible WRA, the participant(s) (child and/or lactating woman) **observation** period will be scheduled directly following recruitment.

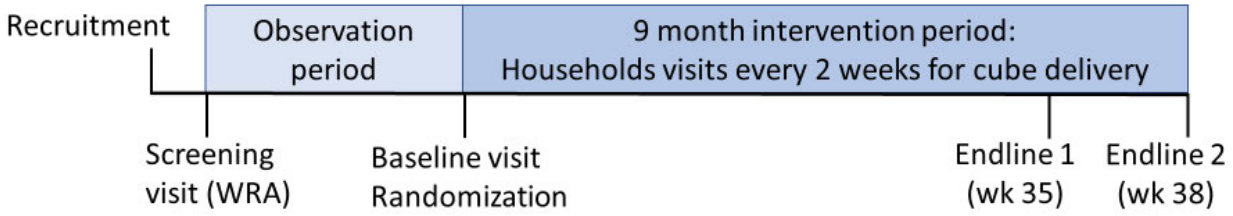

The flow of study activities for each of the 3 target groups (non-pregnant, non-lactating women of reproductive age, non-pregnant lactating women, and children 2-5 years of age), and summary of exclusion and deferral criteria, are shown below in **Figures 2-4**.

Figure 2. Flow of study activities and exclusion and deferral criteria for non-pregnant, non-lactating women of reproductive age.

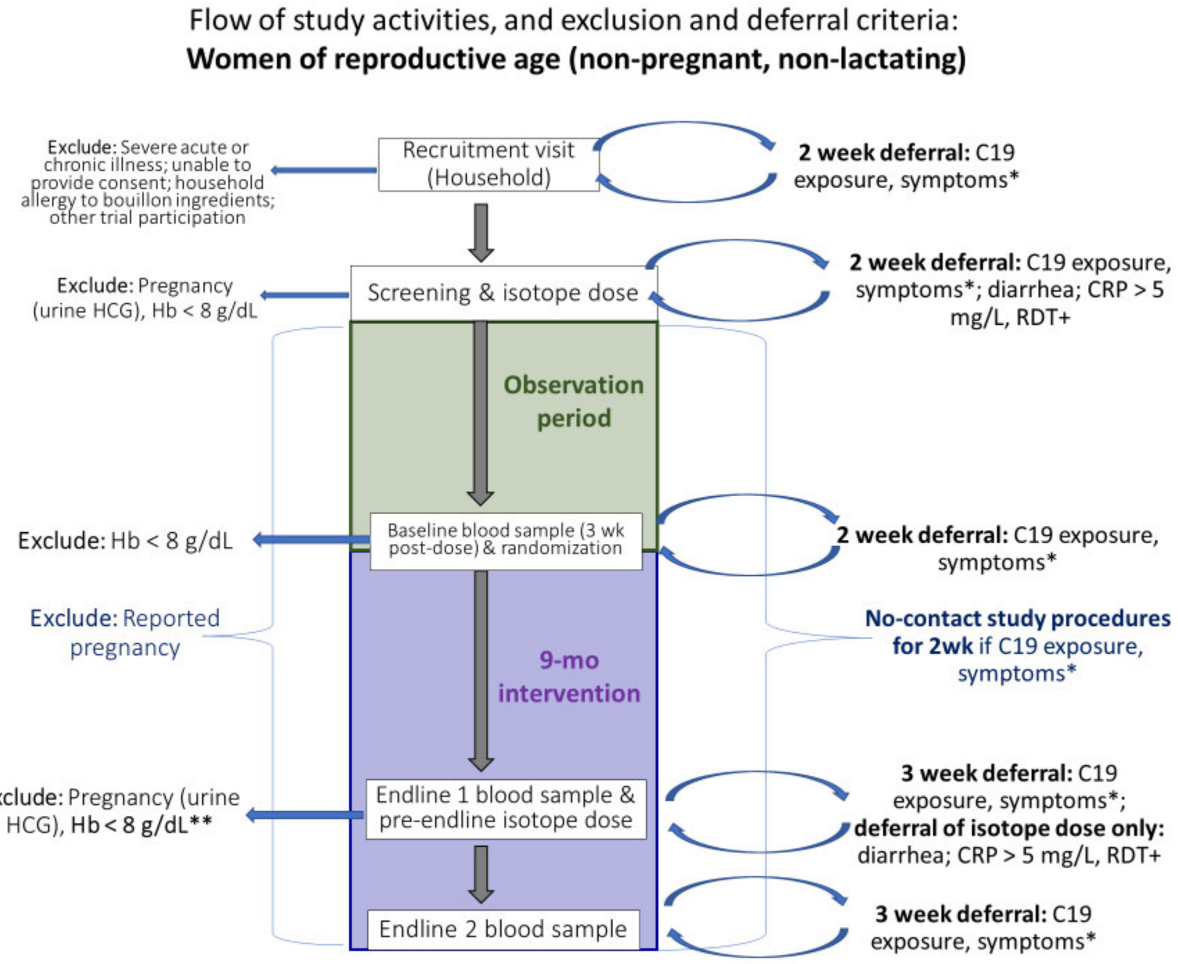

* Defined as participant positive test or COVID-19 diagnosis, or participant exposure to individual with positive COVID-19 test or diagnosis in previous 2 weeks; or selected symptoms experienced by participant (fever, chills/shaking, dry cough, shortness of breath or difficulty breathing, loss of smell or taste within the past 72 hours)

**** Participants with Hb < 8 g/dL at endline 1 will remain in the trial, but will not participate in the pre-endline isotope dosing, or the 2nd endline blood sample.**

Figure 3. Flow of study activities and exclusion and deferral criteria for children 2-5 years of age.

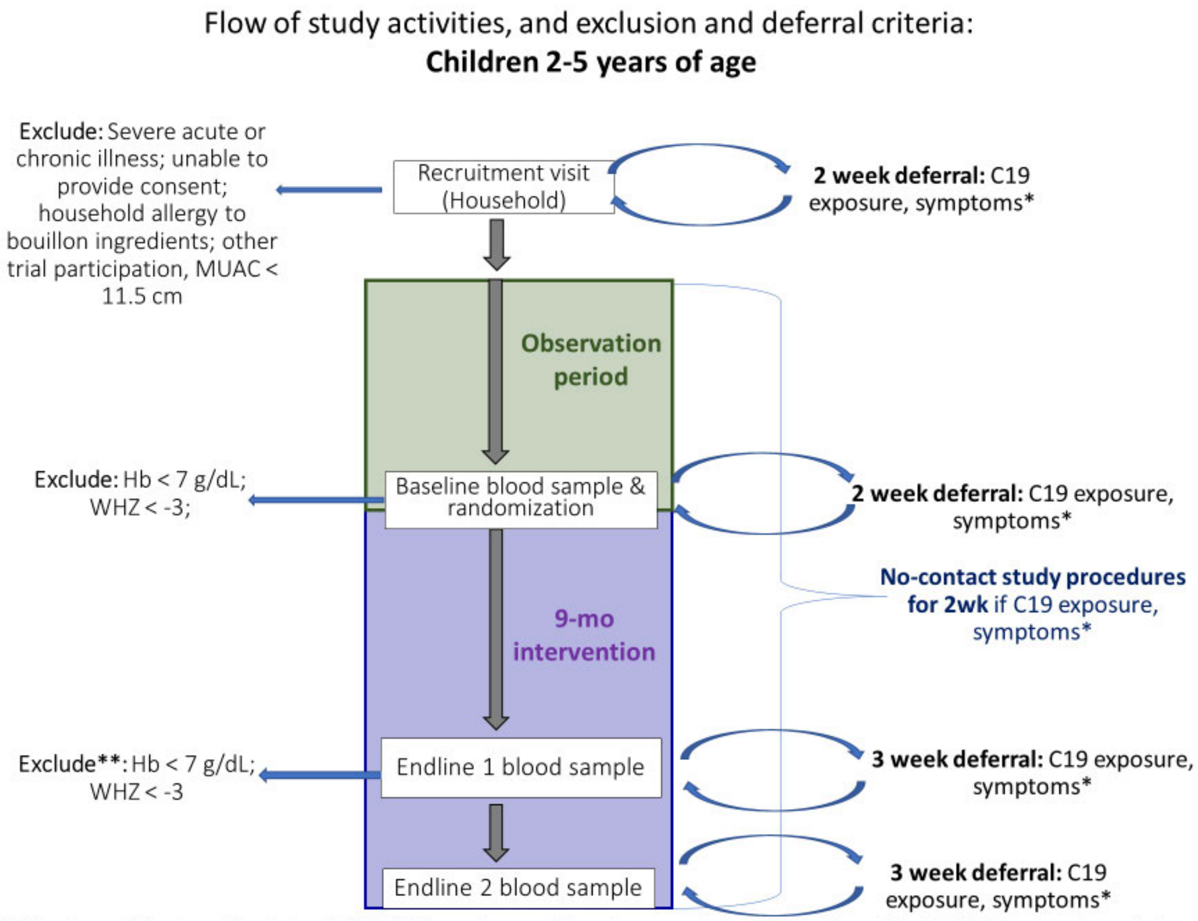

* Defined as participant positive test or COVID-19 diagnosis, or participant exposure to individual with positive COVID-19 test or diagnosis in previous 2 weeks; or selected symptoms experienced by participant (fever, chills/shaking, dry cough, shortness of breath or difficulty breathing, loss of smell or taste within the past 72 hours)

****Participants with Hb 7 8 g/dL or WHZ < -3 at endline 1 will remain in the trial, but will not participate in the 2nd endline blood sample.**

Figure 4. Flow of study activities and exclusion and deferral criteria for non-pregnant, lactating women.

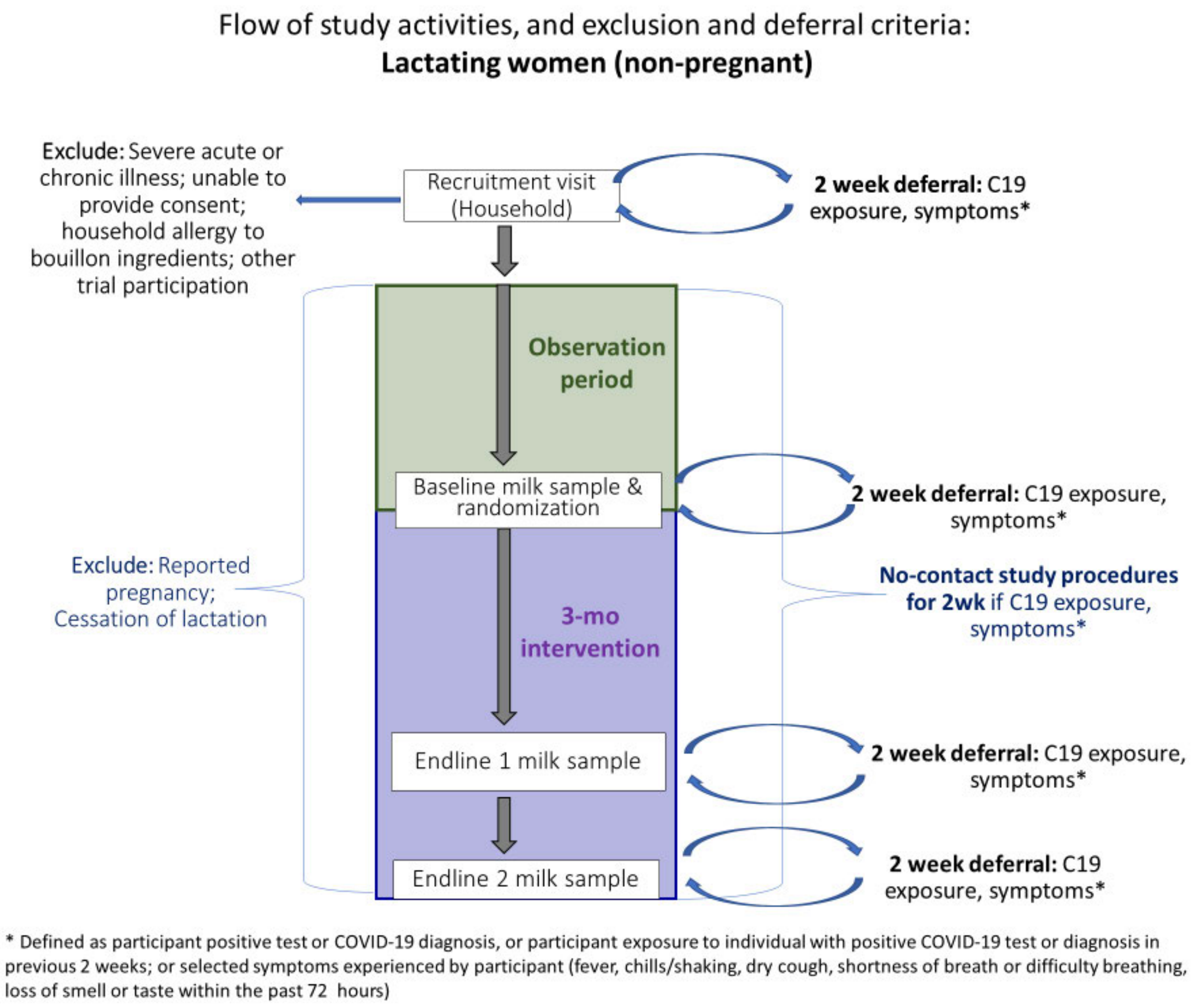

Study site

This proposed research will take place in the Tolon and Kumbungu districts, in the Northern Region. Most people in the area are subsistence farmers (Abizari, Pilime, Armar-Klemesu, & Brouwer, 2013) and the level of poverty is high. The diets consumed in the area mainly consist of local staples including millet, sorghum, cowpea and groundnuts. Malaria is endemic to the region; in pilot data collection, 27% of women and 24% of children showed evidence of current or recent malaria infection (positive RDT; data collected in November-December, 2020).

Being district capital towns, both Tolon and Kumbungu have reasonably good public amenities including treated water, electricity, mobile telephone service, health centres, and a tarred/paved road from Tamale, the largest city in northern Ghana, all of which may facilitate data and sample collection.

Pilot research suggested that food insecurity is widespread in the area: ~95% of households reported some level of food insecurity and 24% reported severe food insecurity (pilot data collected in November-December, 2020). Approximately 10% of women of reproductive age were underweight (BMI < 18.5). Anaemia is common among both women (31%) and children 2-5 years of age (36%). In addition, data on biomarkers of micronutrient status from this pilot work suggest that micronutrient deficiencies are common. For example, iron deficiency (inflammation-adjusted ferritin concentration <15 or <12 µg/L) was observed among 28% of women and 53% of children 2-5 years of age. While vitamin A status was generally adequate among women, 34% of children had evidence of vitamin A deficiency (serum retinol <0.70 µmol/L). In addition, pilot data indicated that 12% of women and 19% of children 2-5 y had serum B12 concentrations <221pmol/L, while low vitamin B12 concentrations in breast milk (<221 pmol/L) were observed among 40% of lactating women.

Pilot research has indicated that fewer than 10% of women and children interviewed had reportedly consumed micronutrient supplements of any kind in the previous 30 days. While staple food fortification is mandatory in Ghana (addition of vitamin A to cooking oil and vitamin A, iron, zinc, and folic acid to wheat flour), maintaining high compliance with target fortification levels is a challenge (Nyumuah et al., 2012). Data on micronutrient levels in food samples collected at the study site indicate that oil samples were fortified with amounts equivalent to ~69% of target vitamin A levels and wheat flour samples were fortified with ~33-34% of target levels for iron and zinc.

Study participants

Participants in the study will be 1) non-pregnant[2], non-lactating women of reproductive age (WRA; 15 - 49 years old), 2) children 2-5 years of age, and 3) non-pregnant, lactating

[2] Pregnant women will be excluded due to the effects of pregnancy on the primary outcome of nutrient biomarker concentrations, which would require different study design and logistics to assess appropriately. Although very early pregnancy may not be self-reported, fluctuations in micronutrient status due to pregnancy are likely to be limited at this very early stage. Participants are tested for pregnancy at the time of vitamin A-isotope dosing (WRA only) and are excluded from the study if pregnant because the RID method for estimating total body vitamin A stores has not been validated among pregnant women. The micronutrient levels included in the study cubes are intended to be safe for all household members, including pregnant women.

women **15 – 49 years old and** 4-18 months postpartum. Multiple participants may be recruited from the same household if they represent different target groups (for example, a woman of reproductive age and child 2-5 years of age), but only one index participant from each target group will be enrolled from each household. To achieve a minimum final sample size of 234 participants per intervention group for each physiological group, and accounting for expected attrition during the study as well as losses during the screening stage we will recruit 1028 WRA, 690 children, and 690 lactating women (see section on Sample Size below for details).

A household is defined as group of people who recognize the same head of household and who live together and share living expenses and meals. If a man has two or more wives and they and their children live and eat together (even if they eat together only sometimes), they form one household. If the wives and their children do not live together in the same compound and always eat separately, they will form more than one household. Members are included if they have lived with the household at least 6 out of the past 12 months. The following exceptions are always household members: the head of household, any child under 9 months of age, and those who intend to stay in the household for at least 6 months.

Inclusion and Exclusion Criteria

The sections below describe inclusion and exclusion criteria at each stage of the study: recruitment, screening (WRA only), baseline, and during the trial. For some criteria (particularly COVID-19-related symptoms) participants may defer the assessment for a 2-week period; if the participant still does not meet the criteria after the 2-week deferment period then they will be excluded.

Inclusion/exclusion criteria at recruitment (home visit):

Household

Inclusion criteria are:

(i) Head of household provides oral consent for the participation of household members (index participants), and willingness to have study-provided bouillon cubes used in their household cooking for the next 10 months;

Exclusion criteria are:

(i) Reported chronic medical condition requiring frequent blood transfusion (e.g. severe forms of thalassemia) among any household members;
(ii) Current participation of any household member in a clinical trial;
(iii)Reported shrimp, wheat, milk, soy, eggs, celery, fish, or mollusk allergy, or a previous adverse reaction to bouillon by the participant or any member of their household.

Non-pregnant, non-lactating women of reproductive age (WRA)

Inclusion criteria are:
(i) Non-pregnant non-lactating women of reproductive age (15 - 49 years);

(ii) Signed the informed consent form (or in the case of adolescents 15-17 years of age, unmarried and still living with their parents, assent provided from the index participant and consent from a parent or guardian);
(iii) Planning to remain in the study area for the next 10 months;
(iv) Willing to use study-provided bouillon in household cooking for the next 10 months;
(v) Not planning to become pregnant during the next 10 months.

Potential participants will be excluded if any of the following apply:
(i) Severe illness warranting immediate hospital referral;
(ii) COVID-19 diagnosis or exposure[3] in the previous two weeks *[individual may repeat eligibility assessment once after a deferral period of at least 2 weeks];*
(iii) Presence of morbidity symptoms suggesting COVID-19 infection (fever [temperature ≥ 38°C], chills/shaking, dry cough, shortness of breath or difficulty breathing, loss of smell or taste within the past 72 hours) *[individual may repeat eligibility assessment once after a deferral period of at least 2 weeks];*
(iv) Chronic severe medical condition (e.g. malignancy) or congenital anomalies requiring frequent medical attention or potentially interfering with nutritional status;
(v) Unable to provide informed consent due to impaired decision-making abilities.

<u>Children 2-5 years of age (24-59 mo)</u>

Inclusion criteria are:
(i) Child 2-5 years of age (24-59 mo);
(ii) Signed informed consent for the child's participation from a parent or guardian;
(iii) Planning to remain in the study area for the next 10 months;
(iv) Caregiver willing to use study-provided bouillon in household cooking for the next 10 months.

Potential participants will be excluded if any of the following apply:
(i) Severe illness warranting immediate hospital referral;
(ii) COVID-19 diagnosis or exposure in the previous two weeks *[individual may repeat eligibility assessment once after a deferral period of at least 2 weeks];*
(iii) Presence of morbidity symptoms suggesting COVID-19 infection (fever, [temperature ≥ 38°C], chills/shaking, dry cough, shortness of breath or difficulty breathing, loss of smell or taste within the past 72 hours) *[individual may repeat eligibility assessment once after a deferral period of at least 2 weeks];*
(iv) Mid-upper arm circumference (MUAC) < 11.5 cm;

[3] Exposure based on the WHO definition: "A contact is defined as anyone who had direct contact or was within 1 metre for at least 15 minutes with a person infected with the virus that causes COVID-19." https://www.who.int/news-room/q-a-detail/coronavirus-disease-covid-19-contact-tracing. Diagnosis may be based on positive test or diagnosis by a medical professional based on clinical symptoms.

(v) Chronic severe medical condition (e.g. malignancy) or congenital anomalies requiring frequent medical attention or potentially interfering with nutritional status.

Lactating women

Inclusion criteria are:

(i) Non-pregnant women of reproductive age (15 - 49 years); currently breastfeeding a child who is 4-18 months of age;
(ii) Signed the informed consent form (or in the case of adolescents 15-17 years of age, unmarried and still living with their parents, provide assent from the index participant and consent from a parent or guardian);
(iii) Planning to remain in the study area for the next 4 months;
(iv) Planning to breastfeed for the next 4 months;
(v) Willing to use study-provided bouillon in household cooking for the next 4 months;
(vi) Not planning to become pregnant during the next 4 months.

Potential participants will be excluded if any of the following apply:

(i) Pregnancy (determined by self-report);
(ii) Severe illness warranting immediate hospital referral;
(iii) COVID-19 diagnosis or exposure in the previous two weeks *[individual may repeat eligibility assessment once after a deferral period of at least 2 weeks];*
(iv) Presence of morbidity symptoms suggesting COVID-19 infection (fever[temperature $\geq$ 38°C], chills/shaking, dry cough, shortness of breath or difficulty breathing, loss of smell or taste within the past 72 hours) *[individual may repeat eligibility assessment once after a deferral period of at least 2 weeks];*
(v) Chronic severe medical condition (e.g. malignancy) or congenital anomalies requiring frequent medical attention or potentially interfering with nutritional status;
(vi) Unable to provide informed consent due to impaired decision-making abilities.

Exclusion criteria at baseline screening visit (WRA only):

Non-pregnant, non-lactating women of reproductive age

Potential participants will be excluded if any of the following apply:

(i) Hemoglobin < 80 g/L **at baseline screening visit**;
(ii) Severe illness warranting immediate hospital referral;
(iii) COVID-19 diagnosis or exposure in the previous two weeks *[individual may repeat eligibility assessment once after a deferral period of at least 2 weeks];*
(iv) Presence of morbidity symptoms suggesting COVID-19 infection (fever [temperature $\geq$ 38°C], chills/shaking, dry cough, shortness of breath or difficulty breathing, loss of smell or taste within the past 72 hours) *[individual may repeat eligibility assessment once after a deferral period of at least 2 weeks];*

(v) Recent diarrhea [≥3 liquid or semiliquid stools in 72 hours]) *[individual may repeat eligibility assessment once after a deferral period of at least 2 weeks];*
(vi) Reported consumption of vitamin A-rich foods (e.g., liver) in the previous 24 hours *[individual may repeat eligibility assessment once after a deferral period of at least 2 weeks];*
(vii) Pregnancy (as ascertained via urine pregnancy test for human chorionic gonadotropin**,** HCG, on the day of isotope dosing);
(viii) Incomplete consumption of vitamin A isotope dose;
(ix) Positive malaria RDT on the day of isotope dosing *[individual may repeat eligibility assessment once after a deferral period of at least 2 weeks];*
(x) CRP > 5 mg/L on the day of isotope dosing *[individual may repeat eligibility assessment once after a deferral period of at least 2 weeks].*

Exclusion criteria at baseline visit:

<u>Non-pregnant, non-lactating women of reproductive age</u>

(i) Hemoglobin < 80 g/L;
(ii) Severe illness warranting immediate hospital referral;
(iii) COVID-19 diagnosis or exposure in the previous two weeks *[individual may repeat eligibility assessment once after a deferral period of at least 2 weeks];*
(iv) Presence of morbidity symptoms suggesting COVID-19 infection (fever [temperature ≥ 38°C], chills/shaking, dry cough, shortness of breath or difficulty breathing, loss of smell or taste within the past 72 hours) *[individual may repeat eligibility assessment once after a deferral period of at least 2 weeks];*
(v) Pregnancy (determined by self-report).

<u>Children 2-5 years of age</u>

(i) Hemoglobin < 70 g/L;
(ii) Severe illness warranting immediate hospital referral;
(iii) COVID-19 diagnosis or exposure in the previous two weeks *[individual may repeat eligibility assessment once after a deferral period of at least 2 weeks];*
(iv) Presence of morbidity symptoms suggesting COVID-19 infection (fever [temperature ≥ 38°C], chills/shaking, dry cough, shortness of breath or difficulty breathing, loss of smell or taste within the past 72 hours) *[individual may repeat eligibility assessment once after a deferral period of at least 2 weeks];*
(v) Severe acute malnutrition at baseline (weight-for-height Z-score < -3 SD or bilateral oedema).

<u>Lactating women</u>

(i) Severe illness warranting immediate hospital referral;
(xi) COVID-19 diagnosis or exposure in the previous two weeks *[individual may repeat eligibility assessment once after a deferral period of at least 2 weeks];*
(xii) Presence of morbidity symptoms suggesting COVID-19 infection (fever [temperature ≥ 38°C], chills/shaking, dry cough, shortness of breath or

difficulty breathing, or loss of taste or smell within the past 72 hours) *[individual may repeat eligibility assessment once after a deferral period of at least 2 weeks];*

(ii) Pregnancy (determined by self-report);

(iii) Cessation of lactation, or planning to discontinue breastfeeding in the next three months.

Exclusion criteria during course of the intervention:

Non-pregnant, non-lactating women of reproductive age

(i) Pregnancy (determined by self-report);

(ii) Pregnancy (as ascertained via urine pregnancy test for human chorionic gonadotropin at the endline 1 visit [pre-endline isotope dosing]).

Lactating women

(i) Cessation of lactation (determined by self-report);

(ii) Pregnancy (determined by self-report).

Note: If a COVID-19 exposure or diagnosis, or if COVID-19-related morbidity symptoms are reported during biweekly visits during the intervention trial, the participant will be allowed to remain enrolled, but field staff will follow procedures for "no contact" study activities (e.g., "no contact" bouillon ration drop-off) for a 2-week period.

Deferral criteria for endline assessments

Replicate endline assessments (3 weeks apart for blood biomarkers and 1 week apart for breast milk biomarkers) will be conducted for each of the primary outcomes except estimation of total body vitamin A stores among women. For all participants, each endline assessment will be deferred if any of the following apply:

(i) COVID-19 exposure or positive test in the previous two weeks *[individual may repeat eligibility assessment once after a deferral period of at least 3 weeks for WRA and children or at least 2 weeks for lactating women];*

(ii) Presence of morbidity symptoms suggesting COVID-19 infection (fever [temperature $\geq$ 38°C], chills/shaking, dry cough, shortness of breath or difficulty breathing, or loss of taste or smell within the past 72 hours) *[individual may repeat eligibility assessment once after a deferral period of at least 3 weeks for WRA and children or at least 2 weeks for lactating women].*

For a participant who has both endline visits deferred, the maximum duration of participation in the study would be 44 weeks (that is, if Endline 1 is deferred from 35 weeks to 38 weeks and Endline 2 is deferred from 41 weeks to 44 weeks). In this case the household would continue to

receive their usual supply of study bouillon cubes until the participants complete the Endline 2 visit. Based on pilot data we expect this situation to be rare.

Note: If a non-pregnant, non-lactating WRA has a hemoglobin concentration < 80 g/L, or a 2-5 year old child has a hemoglobin concentration < 70 g/L or a WHZ < -3 SD at the first endline visit, participants may remain in the trial, but will not participate in the second endline blood sample.

Additional exclusion criteria for pre-endline isotope dose (WRA only):

Non-pregnant, non-lactating women of reproductive age

The pre-endline vitamin A isotope dose will be administered after the endline 1 blood sample. The isotope dose will not be given if any of the following apply:

(i) Hemoglobin < 80 g/L

Additional deferral criteria for pre-endline isotope dose (WRA only):

Non-pregnant, non-lactating women of reproductive age

The pre-endline vitamin A isotope dose will be administered after the endline 1 blood sample. The isotope dose will be deferred if any of the following apply:

(i) Recent diarrhea [≥3 liquid or semiliquid stools in 72 hours]) *[individual may repeat eligibility assessment once after a deferral period of at least 3 weeks];*
(ii) Reported consumption of vitamin A-rich foods (e.g., liver) in the previous 24 hours *[individual may repeat eligibility assessment up once after a deferral period of at least 3 weeks];*
(iii) Positive malaria RDT on the day of isotope dosing *[individual may repeat eligibility assessment once after a deferral period of at least 3 weeks];*
(iv) CRP > 5 mg/L on the day of isotope dosing *[individual may repeat eligibility assessment once after a deferral period of at least 3 weeks].*

Fortified bouillon development

Selection of fortificants and overage

Table 3 summarizes the micronutrient levels and specific chemical forms of micronutrients to be added to the study cubes. The specific fortificants were selected on the basis of technical compatibility with the food matrix and history of use as fortificants. In the case of iron, the combination of ferric pyrophosphate (FePP) with a citric acid/trisodium citrate buffer (CA/TSC) is expected to enhance bioavailability compared to FePP alone (Hackl et al., 2016).

In a parallel set of research activities, investigators at CSIRO and RISE have conducted preliminary testing of the effects of micronutrient addition to bouillon broth on sensory properties and micronutrient stability. These tests suggested that the selected levels for this study will have minimal impact on sensory attributes of the bouillon when cooked. Additional testing of the effects of micronutrient addition to bouillon cubes on the sensory attributes of the cubes and foods prepared with the cubes was conducted from November 23, 2021 – January 28, 2022

(Protocol #GHS-ERC 017/12/20, FDA/FOD/FRD/FER/21/4158 and UC Davis IRB 1687671). This acceptability study was conducted in the Kumbungu and Tolon districts in the Northern Region, where a recent survey showed that micronutrient deficiencies were common. We recruited non-pregnant adult women (15+ years old) (n = 84) who are responsible for household meal preparation, to participate in sensory testing of an uncooked fortified bouillon as well as a cooked food containing the fortified bouillon. Subsequently, participants received a 14-day supply of fortified bouillon cubes to use at home, after which they were asked their opinions about the bouillon. Three fortified bouillon formulations were tested: 1) upper-level bouillon, fortified with iron, zinc, iodine, vitamin A, folic acid, and vitamin B-12 (formulation proposed for the randomized controlled trial and detailed in Table 3 below), 2) lower-level bouillon, containing the same 6 micronutrients but with lower concentrations of some micronutrients (back-up formulation tested in case the upper-level formulation was not acceptable to study participants), and 3) a control bouillon, fortified with iodine only. **There were no significant differences in the mean overall liking of the three formulations when the bouillon was unprepared (dry) or prepared in either of two recipes during the center-based testing. Comparisons of specific attributes of the bouillon (such as taste, color, etc.) were also generally similar across groups. In addition, there was no significant difference in mean overall liking of the three formulations after participants each used one of the three bouillon formulations in their home cooking for two weeks. After this two-week in-home trial, $\geq$ 89% of participants rated their overall liking of the bouillon product formulation to which they were randomized as "like" (4) or "like very much" (5), and this did not differ among groups. Overall, the study results suggested that all three bouillon formulations were well liked and would be suitable for use in further research studies.**

Overage values to account for micronutrient loss **during storage and cooking were selected based on industry experience and storage and cooking trials conducted by CSIRO and RISE: 30%** for vitamin A, folic acid, **and** vitamin B12, **20% for iodine,** and **0**% for iron **and** zinc (these estimates are in addition to the target levels shown in **Table 3**).

In the future, innovations in fortificant forms, such as those with greater expected bioavailability and/or stability, may be considered for inclusion in this research. In such case, the details of the cube formulation and proposed modification will be submitted to the Ghana FDA and ethical review committees of the Ghana Health Service and UC Davis for approval.

Table 3. Chemical form and concentration of micronutrients to be added to each of the study bouillon cubes

Cube name	Target micronutrient content
Multiple MN-fortified bouillon cube	200 µg/g Vitamin A (retinyl palmitate)<br>80 µg/g Folic acid<br>1.2 µg/g Vitamin B12<br>3 mg/g Zinc (ZnO)<br>4 mg/g Iron (FePP/CA/TSC)<br>30 µg/g Iodine (KIO_3)
Iodine-fortified bouillon cube (control)	30 µg/g Iodine (KIO_3)

FePP/CA/TSC: ferric pyrophosphate/citric acid/trisodium citrate. Values do not include overage.

Selection of micronutrient concentrations for each study group

We selected micronutrient concentrations for the bouillon cubes that represent the best estimate of fortification levels that will be both efficacious for improving micronutrient status and reducing deficiency as well as safe for consumption by all household members, based on the modelling and literature review described below, and given what is known about micronutrient status and bouillon cube consumption at the study site. **Table 4** presents the micronutrient concentrations and equivalent target daily micronutrient doses for women and young children in the active arm of the study.

Selection of target fortification levels

The concentrations of micronutrients to be included in the multiple micronutrient-fortified bouillon were selected based on 5 types of information:

a) Modelling of national survey data from Ghana and other West African countries to estimate the contribution of hypothetical fortification of bouillon to adequacy of dietary micronutrient intake (to estimate the potential public health benefit)
b) Estimated amount of bouillon consumed in the communities selected for the study
c) Expected efficacy of selected daily micronutrient doses, based on a review of the literature on the effect of micronutrient interventions on change in micronutrient status as measured by biomarkers or in clinical outcomes (anemia and neural tube defects) and estimated accumulation of micronutrient stores (vitamin A and iron)
d) Safety considerations, including the likelihood of study participants or their household members exceeding the UL, and review of the literature to identify adverse effects of micronutrient interventions at varying daily micronutrient doses.
e) Effect of micronutrient fortification on sensory properties of the bouillon, and micronutrient stability

Please see attached Technical Appendix for additional detail on the rationale for the selected fortification levels and estimated daily micronutrient doses.

The Control cube formulation contains iodine but no other added micronutrients. This composition is consistent with current policy in Ghana, where salt iodization is mandated (15 mg/kg at household level) and commercial bouillon cubes are typically made with iodized salt. For this research study, due to concerns about variability in salt iodine content across batches, bouillon cubes will be made with non-iodized salt and equivalent levels of iodine will be added during the bouillon manufacturing process, to ensure standardization of iodine content of bouillon across treatment groups and over time.

Table 4. Target fortification levels, estimated daily dose of micronutrients delivered by fortified bouillon, and percent of the recommended daily allowance for women of reproductive age and young children.

Micronutrient	Target micronutrient concentration in bouillon cubes	Daily dose to women, assuming 2-2.5 g cube/day	% RDA for women 19-50 years, assuming 2-2.5 g cube/day	Daily dose to children, assuming 1 g cube/day	% RDA for children 1-3 years or 4-8 years, assuming 1 g cube/day
Vitamin A	200 µg/g	400 - 500 µg/d	57 - 71%	200 µg/d	50 - 67%
Folic acid	80 µg/g	160 - 200 µg/d	67 - 83%	80 µg/d	67 - 89%
Vitamin B12	1.2 µg/g	2.4 - 3.0 µg/d	100 - 125%	1.2 µg/d	100 - 133%
Iron	4 mg/g	8 - 10 mg/d	44 - 56%	4 mg/d	40 - 57%
Zinc	3 mg/g	6 - 7.5 mg/d	75 - 94%	3 mg/d	60 - 100%
Iodine	30 µg/g	60 - 75 µg/d	40 - 50%	30 µg/d	33%

The Recommended Daily Allowance (RDA) for women 19-50 years of age (US Institute of Medicine) is 700 µg RAE/d (vitamin A); 400 µg DFE/d, equivalent to 240 µg folic acid/d (folate); 2.4 µg vitamin B12/d; 18 mg iron/d; 8 mg zinc/d; and 150 µg iodine/d. For children 1-3 y and 4-8 y of age, the Recommended Daily Allowance is 300 and 400 µg RAE/d (vitamin A); 150 and 200 µg DFE/d, equivalent to 90 and 120 µg folic acid/d (folate); 0.9 and 1.2 µg vitamin B12/d; 7 and 10 mg iron/d; 3 and 5 mg zinc/d; and 90 µg iodine/d. Values do not include overage.

Bouillon production and shipping

Bouillon will be produced at industrial scale by GB Foods in Spain. Products will be labelled as described below (Section "Randomization and Blinding") to ensure blinding of the research team members and participants. Product documentation will be submitted to the Ghana FDA, as well as to the relevant ethical review committees, for approval prior to initiating the study.

Management of bouillon stocks

Bouillon will be received by the University of Ghana in Accra and transported to the study office at the field site. Boxes of the bouillon will be stored on pallets in a cool environment at the central study office or a dedicated storage facility with frequent temperature and humidity monitoring. We will consult with the manufacturer beforehand regarding expiration dates, and we will arrange product shipments accordingly.

In each shipment of bouillon to Ghana, a random sample of the bouillon cubes will be analysed for selected nutrient contents (with the results examined by someone external to the study team, to maintain blinding) to ensure proper coding and formulation of the bouillon cubes before distribution in the field.

Study procedures

COVID-19 precautions

As noted below, participants with fever or known COVID-19 exposure on the day of data collection will be deferred/excluded. Data collectors and participants will adhere to **COVID-19 prevention guidance as recommended by** Ghana Health Service**, to the extent that study procedures may allow. These guidelines may include handwashing/hand sanitizing, protective mask use, and physical distancing (participants and/or data collectors spaced a minimum of 1 meter apart)**. Additional information is given below (Section: Risks to Participants).

Overview

A summary of data collection procedures is shown in **Table 5**.

Table 5. Summary of data collection procedures[4]

	Recruit (in-home visit)	Screen (WRA)	Observation period	Baseline visit	Intervention Period				
					Initial Home Visit	Biweekly	4-weekly	Endline 1	Endline 2
**Questionnaires/observations**									
COVID-19 Screen, Morbidity surveillance, **AE/SAE**	X	X	X	X	**X**	X		X	X
**Exclusion criteria**	**X**	**X**		**X**	**X**	**X**		**X**	**X**
HH roster; socio-economic status	X								
HH bouillon **acquisition, consumption, stocks** (e.g., FACT, KAP, acceptability, **adherence**)	X		X		**X**	**X**		**X**	**X**
HH food & water security (HFIAS, HWISE)	X				**X**		X		
Health **history; demographic, health & lifestyle**			**X**	**X**	**X**				
**Diet (24h recalls; 2 per person)**			**X**		**X + 30 d**				**X – 30 d**
**Diet (e.g., FFQ, MDD-W, IYCF)**					**X**		**X**	**X**	**X**
**WTP**	**X**								**X**
**Child development (MDAT, HOME,EYT)**			**X**						**X – 21 d**
**OAA study**					**X + 60 d**	**(FGDs)**			**X – 60 d**

[4] AE, adverse events; APP, acute phase proteins (markers of inflammation); BP, blood pressure; CRP, C-reactive protein; **EYT, Early Years Toolbox;** FACT, Fortification Assessment Coverage Tool; **FGD, Focus group discussion;** FFQ, food frequency questionnaire; Hb, hemoglobin; HFIAS, Household Food Insecurity and Access Scale; HH, household; **IYCF, Infant and young child feeding;** KAP, Knowledge, attitudes and practices; MDD-W, minimum dietary diversity-women; MN, micronutrient biomarkers; MUAC, mid-upper arm circumference; **OAA, Opinions, Attitudes, and Acceptance;** RDT, rapid diagnostic test (for malaria); RID, retinol isotope dilution; SAE, severe adverse events; STEPS, WHO STEPs questionnaire on non-communicable disease risk; WRA, (non-pregnant, non-lactating) women of reproductive age; WTP, willingness-to-pay. RID screening includes: Hb, RDT, CRP, pregnancy test; oral vitamin A isotope dose. Morbidity surveillance includes symptom reporting, body temperature, and AE/SAE.

**Biospecimens**									
Anthropometry	(MUAC)			X				X	
Retinol isotope dilution (RID) screening (WRA)		X						X	
RID blood sample (WRA)				X					X
Blood collection (e.g., Hb, RDT, MN, APP)				X				X	X
**Blood pressure**		**X**		**X**				**X**	**X**
Milk collection				X				X	X
Urine collection		X		X				X	
**Stool collection (child)**				**X**				**X**	

Recruitment

Besides the ethics approval from the GHS-ERC, we will seek permission from the Regional (Northern Region) and District (Tolon and Kumbungu) Directors of Health of Services as well as community leaders before recruiting participants into the study. With the assistance of the local health personnel, we will then inform community leaders and residents of each of the communities in the catchment area about the objectives, methods and possible benefits of the study for the study participants and communities. In order to do so, we will hold general informational meetings with traditional leaders in the communities and other residents who express an interest in attending.

We will consult district and cluster maps to select communities and clusters within the Kumbungu and Tolon districts, including urban, peri-urban and rural areas. Recruitment of each physiological group (WRA, children, lactating women) will be balanced within each cluster so that the final sample of participants in each target group represents a similar geographic area. Multiple target groups may be enrolled from the same household (e.g. a lactating woman and a child 2-5 y) but only one participant from each target group will be enrolled from each household.

Recruitment and questionnaire administration will be conducted by trained interviewers who are fluent in English and the local language (mainly Dagbani). Because many people do not read or write the **local** language well, the questionnaires will not be translated into Dagbani, but interviewers will receive instructions for on-site translation of the questionnaires during training. The survey instruments have been pre-tested in the pilot survey and acceptability study that have proceeded this trial, and revised accordingly to ensure that all questions will be understood by participants and to minimize the time required to administer the interviews.

Recruitment will proceed as follows:

i. Households in selected communities within the Kumbungu and Tolon districts will be identified through a random walk method with door-to-door recruitment. Data collectors will follow a specific sampling protocol to select households to approach to assess eligibility. A household ID will be assigned to each household approached.

ii. When approaching a household for recruitment, data collectors will obtain verbal informed consent from the household head (or knowledgeable adult household member who is present), to assess eligibility for the study. Verbal consent will be documented by the data collector, and the data collector will then administer a brief questionnaire to assess exposure to COVID-19 by any household members and measure the temperature of the respondent. In the case of reported COVID-19 exposure or respondent with fever, the interview will be discontinued, and the household will be advised to seek medical advice.

iii. Next, a brief questionnaire will be conducted to determine whether the household includes any potentially eligible participants (i.e., non-pregnant, non-lactating women age 15 – 49 years, children 2-5 years, or non-pregnant lactating women 15-49 years with a child 4-18 months of age). If the household includes any potentially eligible participants, the data

collector will briefly describe the study procedures to the head of household, including the use of study bouillon cubes in household cooking. The data collector will document verbal agreement by the head of household that they support the participation of household members in the study. If the head of household agrees, **the data collector will ask a few questions about household exclusion criteria and then complete** a detailed household roster. If the head of household is not present in the home at the time, the data collector will schedule a return visit.

iv. Participants found eligible (based on recruitment inclusion/exclusion criteria) will be ***recruited*** after data collectors have explained the study procedures, and obtain informed consent. Only one household member per physiological group (i.e., non-pregnant, non-lactating women age 15 – 49 years, children 2-5 years, or non-pregnant lactating women 15-49 years with a child 4-18 months of age) will be ***recruited*** in the trial. Up to three household members (one from each physiological group) per household may be ***recruited*** if all are found to be eligible based on recruitment inclusion/exclusion criteria.

a. If anyone in the household matches current recruitment needs, and there is no more than one eligible individual currently present in each target group, the data collector will determine if they are potentially interested to participate. If so, the data collector will administer a separate questionnaire to the woman (or caregiver, in the case of the child) to determine eligibility using the inclusion and exclusion criteria listed above in the recruitment (home visit) section. If the woman (or child) meets eligibility criteria, then the data collector will explain the study procedures in detail, answer any questions the participant has, obtain and document written consent to participate. At this point, the enumerators will assign a participant ID to the individual participant.
b. If anyone in the household matches current recruitment needs, and there are more than one potentially eligible individuals per participant group who are currently present in the home (for example, if there are two children between 2-5 years of age), we will randomly select one individual per group to be enrolled, based on a Kish Table, and conduct the aforementioned enrolment procedures. If this individual does not provide informed written consent, or does not meet inclusion and exclusion criteria during recruitment, then we will randomly select another potentially eligible individual from the same participant group and repeat the aforementioned enrolment procedures.
c. If the household composition does not match current recruitment needs, or household members are not currently present in the home, the data collector will ask whether or not the household would be willing to be re-contacted in case enrolment needs change.

v. For those participants **recruited** in the study, the data collector will then conduct an initial interview to assess demographics and household characteristics, including indicators of socioeconomic status (housing characteristics, possessions), household consumption of fortified foods and typical patterns of salt and bouillon cube use.

vi. **Recruited** non-pregnant, non-lactating women of reproductive age (WRA) will then be invited to the mobile biospecimen collection site for the biospecimen screening visit to confirm eligibility; children and lactating women are now considered **recruited** for the **observation** period. For participants in households in which there is a potentially eligible WRA, the observation period will be scheduled after the WRA screening visit. For

participants in which there is no potentially eligible WRA, the participant(s) (child and/or lactating woman) will continue directly to scheduling the **observation** period**.**

vii. **Finally, enumerators will identify the household member who is primarily responsible for making decisions about bouillon purchasing to administer a brief questionnaire to assess perceptions of fortified bouillon cube and their hypothetical willingness to pay (WTP) for a new fortified bouillon cube.**

Biospecimen screening, for non-pregnant, non-lactating women of reproductive age

Upon arriving at the biospecimen collection site, participants will first be screened for COVID-19 by questionnaire and temperature check by an enumerator. Anyone with a recent COVID-19 diagnosis or known COVID-19 exposure, fever [temperature ≥ 38°C], chills/shaking, dry cough, shortness of breath or difficulty breathing, or loss of taste or smell within the last **72**h, or signs of severe illness (i.e., unresponsive) will be referred for medical treatment and no further procedures will be conducted that day. That participant, and any potentially eligible participants from that household, may resume study procedures after a 2-week deferral period.

Data collection at the biospecimen screening visit will include the following procedures:

1. Assessment of recent morbidity symptoms and vitamin A intake by enumerator-administered questionnaire: Participants who do not meet all criteria to continue to isotope dosing (as described above) will have the opportunity to repeat the screening a second time after at least 2 weeks have passed.
2. Pregnancy test: Women will be asked to provide a spot urine sample (~10ml) for pregnancy testing based on human chorionic gonadotropin in urine conducted by a nurse or a study enumerator. Participants with a positive pregnancy test will be excluded from the study. Aliquots will also be retained as an additional time point to increase the precision of assessment of urinary sodium and potassium concentrations.
3. Blood pressure: Blood pressure will be measured using a portable device.
4. Capillary blood collection: Trained phlebotomists will collect a capillary blood sample. The QuikRead Go instrument will be used to simultaneously measure haemoglobin and C-reactive protein. Measurement of inflammation is necessary to assess eligibility to receive the vitamin A isotope for assessment of vitamin A total body stores. Individuals with severe anaemia will be excluded from the study **at the baseline screening visit** and referred for medical evaluation. **At the endline screening visit, they will not receive the vitamin A isotope and they will not be scheduled for blood collection at the endline 2 visit but they will not be excluded from the study. They will be referred for medical evaluation and will remain eligible to continue with other study activities.** WRA with CRP > 5 mg/L will be excluded from further data collection on this day; these individuals will be eligible to be re-screened after at least 2 weeks have passed. Current or recent malaria infection will be assessed by rapid diagnostic test. Individuals with positive malaria RDT (indicating current or recent malaria infection) **will be** excluded from isotope dosing on this day. These individuals will be referred for treatment, and they will be eligible to be re-screened after at least 2 weeks have passed.
5. Isotope dosing: Women who meet the inclusion and exclusion criteria will receive a **2 mg** oral dose of d6-labelled vitamin A ("heavy" vitamin A) in oil, administered by a senior staff member, for assessment of baseline total body vitamin A stores using the retinol isotope dilution method. The isotope will be sourced from the company Buchem BV (Apeldorn, Netherlands), certified as food grade (Food Chemical Codex IV) and **will be** approved by the Ghana Food and Drugs Authority. Women will be

provided with a high fat snack (e.g., crackers with chocolate spread) to aid in absorption of the isotope dose. These women will subsequently be asked to avoid consuming high-vitamin A foods (liver, organ meats) and to avoid taking a micronutrient supplement for the rest of the day.

Participants that are found to be eligible for the study after completing the screening visit will continue into the **observation** period. All WRA who received the d6-labelled vitamin A dose will be invited to the biospecimen collection site 21 days (based on Green et al., 2021) after the isotope dose for a venous blood sample to assess total body vitamin A stores using the retinol isotope dilution (RID) technique. This 21-day visit will be scheduled to coincide with the trial baseline visit (procedures described below).

If the participant is excluded at the screening visit, depending on the status of recruitment, the study team will 1) continue with enrolment of children or lactating women (if any) from the same household or 2) determine if there are any other potentially eligible WRA from the same household and, if so, revisit the household to determine whether another WRA in the household is eligible and willing to participate. In the latter case, a new screening visit would be scheduled for the second WRA.

If there are multiple potentially eligible members of the same household and the WRA is eligible for deferred screening (i.e., due to elevated CRP or positive RDT on the day of screening), depending on the status of recruitment, the household may be given the option of 1) deferring enrolment of all participants and repeating the biospecimen screening for the non-pregnant non-lactating woman at a later date (at least 2 weeks later) or 2) continuing with enrolment only of the other household member(s).

Observation period

The 3-week time period between the WRA screening visit and the baseline visit will serve as an observation period during which additional detailed information will be collected about household bouillon consumption and use. It will also allow for baseline child development tasks to be completed prior to randomization and delivery of the intervention.

Procedures
Within approximately a week of recruitment (or as soon as possible after the screening visit if the household includes a WRA), an enumerator will visit the participating household to document current stocks of commercial bouillon products **and administer questionnaires to collect information on household bouillon purchasing and consumption patterns, household cooking arrangements (for example, number of cooks in the household), and meal sharing with non-household members. The enumerator will also gather information on health history, including receipt of essential interventions. For women, enumerators will administer an individual demographic, health and lifestyle questionnaire which includes questions from the WHO STEPwise Approach to NCD Risk Factor Surveillance (STEPS) questionnaire to assess health behaviors related to non-communicable disease risk. A second data collection visit will be conducted 2 weeks after the previous home visit to repeat the assessment of household bouillon stocks and recent bouillon acquisition and consumption.**

During the observation period, information on total dietary intakes will be collected by 24-hour dietary recall interview from each participant. This will enable comparison of dietary intake before and after households begin to receive the study bouillon cubes, and assessment of whether any change in bouillon consumption differs by intervention group. Interviews will be conducted by trained staff during a participant home visit. The interview will use a tablet-based data collection form (modified version of OpenDRS) to collect information on all foods and beverages consumed by the respondent on the day prior to the interview. For children, the interview will be administered to the primary caregiver of the child, with assistance from other household members as necessary to report all foods and beverages consumed by the child. For selected mixed dishes (e.g. with focus on those commonly containing bouillon), the interview will include collection of recipe information for mixed dishes prepared at home, including the type and amount of ingredients and the total quantity prepared.

During one of the home visits for the **observation period**, child development assessments will be conducted by trained study staff members. The data collector will administer a brief questionnaire to the child's caregiver to obtain background information about the child **and the child's primary guardian** (e.g., previous exposure to different languages or to mobile technology). The data collector will then administer the Home Observation Measurement of the Environment (HOME) tool, which includes questions addressed to the caregiver and observations of the home environment (such as availability of books and toys) and caregiver-child interactions. **The child will then be invited to a central location (see below) for** the MDAT assessment (Gladstone et al., 2010). The MDAT involves a brief series of tasks to be completed by the child (for example, building a tower of blocks or responding to questions or commands), along with observations by the data collector and questions addressed to the child's caregiver. The data collector will record the scores on each task, which are used to measure four domains of development (gross motor, fine motor, language, and personal-social). **Next, the data collector will administer the tablet-based assessment, the Early Years Toolbox (Howard et al., 2017). The Early Years Toolbox (EYT) involves a series of brief tablet-based games designed to measure children's cognitive development. For this task children are shown a series of images (such as fish and sharks) and are asked to tap or not tap on certain images. Information such as reaction time and the number of correct responses is recorded by the program for later analysis. The task takes about 5-8 minutes, including practice runs. If caregivers are unable to bring the children to the data collection site, the MDAT and tablet assessment may be completed at the participant's home in a space outside the home with minimal distractions. In a convenience subset of 30 children, the EYT assessment will be repeated approximately 1 week later (at the second observation home visit or at the baseline visit) to calculate test-retest reliability.**

Baseline visit

Eligible participants will be invited to the mobile biospecimen collection site after completion of the **observation** period for the baseline visit.

Upon arriving at the mobile biospecimen collection site, participants will first be screened for COVID-19 by questionnaire and temperature check. Anyone with a COVID-19 diagnosis or exposure in the previous 2 weeks, or morbidity symptoms possibly indicating COVID-19 (fever [temperature $\geq$38°C], chills/shaking, dry cough, shortness of breath or difficulty breathing, or

loss of taste or smell within the last 72h), or signs of severe illness (i.e., unresponsive) will be referred for medical treatment and will not participate in further data collection that day. Individuals with COVID-19 diagnosis, exposure, or recent symptoms may repeat the assessments after a period of at least 2 weeks; if the participant does not pass these criteria at the second visit, they will be excluded from the study.

Following the COVID-19 screening, participants will undergo baseline data collection procedures, as described below. Eligibility to participate in the trial will be based on the inclusion and exclusion criteria detailed above for the baseline visit (Inclusion and Exclusion Criteria). Following the baseline data collection procedures, participants will receive their group assignment for the remainder of the trial (see Section 7 below on Randomization and Blinding).

Data collection at the baseline visit will include the following procedures:

1) Non-pregnant, non-lactating women (WRA)
    1. Anthropometry: Height, weight, and waist and hip circumference of WRA will be measured (see detailed procedures below).
    2. Blood pressure: Blood pressure will be measured using a portable upper arm blood pressure monitor.
    3. Urine sample: A spot urine sample collection will be conducted to measure urinary iodine, sodium, and potassium concentrations.
    4. Venous blood collection: WRA will be requested to arrive at the mobile biospecimen collection site after an overnight fast (> 8 hours). Venous blood (up to 12 mL) will be collected for immediate analysis of haemoglobin (HemoCue), malaria (by RDT), **ZPP (iCheck Anemia)** and haematocrit. In addition, aliquots of whole blood, serum, and plasma will be retained for micronutrient biomarker analysis (see detail below). Following blood collection, WRA will receive a light breakfast. The light breakfast will consist of 1-2 slices of bread with chocolate spread and malt drink. We will avoid any food item with high susceptibility to microbial spoilage.
    5. Questionnaires: Interviewers will administer questionnaires to collect individual level information on non-COVID-related morbidity history**, health and reproductive history, fasting status, and other information related to interpretation of biospecimen results.**

2) Children 2-5 years
    1. Anthropometry: Height, weight, and mid-upper arm circumference of children will be measured (see detailed procedures below). Children with severe acute malnutrition, defined as weight-for-height Z score < -3 SD or bilateral oedema, will be excluded.
    2. Blood pressure: Blood pressure will be measured using a portable upper arm blood pressure monitor.
    3. Stool sample: During the **observation** period, caregivers will be provided with collection containers and instructions for collecting child stool (see detailed procedures below). Caregivers will be instructed to bring the container to the mobile biospecimen collection site.
    4. Urine sample: A spot urine sample collection will be conducted to measure urinary iodine and sodium concentrations.
    5. Venous blood collection: Venous blood (up to 7.5 mL) will be collected for immediate analysis of haemoglobin (HemoCue)**, ZPP (iCheck Anemia)** and

malaria (by RDT). In addition, aliquots of **plasma** will be retained for micronutrient biomarker analysis (see detail below). Children will not be requested to fast prior to the blood sample, but time of the most recent eating occasion will be recorded.

6. Questionnaires: **Interviewers will administer questionnaires to collect individual level information on non-COVID-related morbidity history, health history, fasting status, and other information related to interpretation of biospecimen results.**

3) Lactating women
   1. Anthropometry: Height and weight of WRA will be measured (see detailed procedures below).
   2. Milk collection: Lactating women will be requested to arrive at the mobile biospecimen collection site after an overnight fast (> 8 hours). A full milk sample will be collected using a breast pump. From the full milk sample, a ~10ml aliquot of well-mixed milk will be retained for analysis of milk fat (onsite using the Creamatocrit Plus) and micronutrients (with the remainder returned to the mother to be fed to the child with a cup and spoon). Lactating women will be provided with a light breakfast after milk samples are collected.
   3. Questionnaires: **Interviewers will administer questionnaires to collect individual level information on non-COVID-related morbidity history, health and reproductive history, fasting status, and other information related to interpretation of biospecimen results.**

Following baseline data collection, participants will be randomized to their group assignment **and considered to be *enrolled* into** the remainder of the trial (see details below). Field staff will then arrange a time for an enumerator to visit the household on the following day to deliver the first bouillon ration that corresponds to the assigned treatment group. **Participants and their households will be requested to use the study cubes in place of commercial cubes in any dish prepared for household consumption that typically includes bouillon as an ingredient. In the case of multiple cooks and/or bouillon storage locations in the household (for example, multiple wives in a polygamous household), participants will be asked to distribute the bouillon cubes among cooks as they would typically do. The enumerator will document the brand(s) and quantity of commercial bouillon cube stocks in the household. The enumerator will then request to collect all commercial bouillon in the household; households will be compensated according to a table of current brand-specific market prices for bouillon (up to a certain quantity, e.g. 2 cartons). The enumerator will provide an opportunity to hear questions or concerns from any household members during this visit and will rely on scripted 'talking points' to address questions and potential barriers to adherence.** Additional questionnaires to collect baseline information on **household food insecurity, water insecurity, consumption of fortified foods, and dietary patterns (e.g., dietary diversity and child feeding)** will also be administered during this visit**; these questionnaires will be administered every 4 weeks thereafter (Table 5)**.

For all groups, baseline dietary assessment will be conducted by administering two 24-hour dietary recall interviews per participant on non-consecutive days within 30 days after the baseline visit**, using the modified OpenDRS platform, as described above**.

Randomization and blinding

The unit of randomization will be the household level, regardless of the number of individual participants eligible within that household. Once one or more participants within a household have been confirmed to be eligible for the study and successfully completed the **baseline visit**, the participants will be enrolled in the full trial.

UC Davis statisticians will create a computer-generated block randomization scheme with randomly selected block lengths of four or eight. Sheets bearing supplement allocations represented by 4 different color and number codes (2 per study arm) and numbered 1–2408 (maximum number of households if each participant resides in a different household) will be placed in opaque envelopes and stacked in increasing order. At each enrollment, the assigned study enumerator will select the four topmost envelopes on the stack and ask the participant to choose an envelope to reveal their study group allocation.

At this point, the household ID (and the participant IDs for each eligible participant within the household) will be linked to one of the four study codes indicating the intervention that the household will receive.

All study bouillon cubes will be individually wrapped in identical packaging and will be identified only by the study code (a 3-digit code). Boxes of cubes will be labelled with the study code and the associated group colour. Participants and study personnel will know the group colour to which each household is assigned but will not know the micronutrient content of the products.

Two code keepers (one at UC Davis and one at U Ghana) who are not otherwise affiliated with the study will be responsible for assigning the 4 study codes to the intervention product and communicating directly with the manufacturer about the group assignments. In addition, a hard copy of the randomization code will be stored in a sealed envelope in in a locked filing cabinet at the University of Ghana. Two individuals, unaffiliated with the study, will have a key to the office and access to the key to the filing cabinet.

In the case of an emergency pertaining to an individual participant, the blinding code will be broken only when the identity of the investigational product that the participant received will determine the treatment to be given to that participant for the emergency. Only individuals directly involved in the treatment of the study participant should be unblinded on a strict need-to-know basis.

The Ghana Health Service Ethical Review Committee, Ghana Food and Drugs Authority, UC Davis Institutional Review Board, or the DSMB may request unblinded safety data to discuss in closed sessions. Unblinding of the treatment may be requested if there is a pattern of SAEs which may be related to study treatment where the DSMB or other regulatory agency feels there is “potential for harm”. In this case, the randomization code will be provided by one of the two code keepers.

The specific product associated with each of the study codes will also be stored in sealed envelopes held by the co-principal investigators (PIs) and the statistician. The envelopes will be opened only after statistical analyses of the primary outcomes are completed and the investigators have reached consensus on interpretation of the results.

<u>Calculation of household bouillon ration</u>

Households will be randomly assigned to receive one of two types of bouillon cubes to be used in household cooking for the duration of the trial. Bouillon rations will be delivered by enumerators to participants' homes every 2 weeks. The household will be instructed to distribute and store the study-provided bouillon cubes as they would typically do with bouillon they purchase (such that all household members, and not just the index participant(s), have bouillon available for their household cooking). Households will be provided with plastic storage containers, in which to keep the bouillon.

The household bouillon ration will be determined based on household size (number of individuals in the household) and estimates of per capita bouillon consumption from previous research in the study community (pilot survey and acceptability study). Analysis of pilot data showed that calculating rations based on number of people in the household gave similar results compared to using the adult male equivalent (AME) method to account for the age and sex of household members. The household's assigned bouillon ration will be evaluated based on observations and questionnaire responses throughout the intervention period and adjusted if necessary. To discourage cube sharing or excessive consumption, household rations will not exceed 4 g/capita/d, even if households report consistently consuming more than this amount. Households will be encouraged to use the study bouillon according to their typical cooking habits, and they are free to purchase additional commercial bouillon cubes if they choose.

Intervention period procedures

Each visit will begin with screening the participant (and the caregiver, who will respond on behalf of participating children) for COVID-19 exposure and symptoms, including measurement of body temperature. If COVID-19 exposure or symptoms are reported, the study staff member will end the interview, advise the participant to seek medical advice, and conduct a "no-contact" bouillon drop-off for the household ration. In this case, the participant will be requested to do a "no contact" transfer of bouillon wrappers and data collection for that time point (e.g., morbidity and adherence) will be conducted by phone if feasible. To conduct data collection by telephone, the enumerator will arrange a time to call the participant by phone to administer the questionnaires assigned for the specific time point. The enumerator will read all questions to the participant and record responses in the same way as for in-person visits. Any responses requiring observations (such as observations of bouillon storage conditions) may be left blank. Calls will be placed from a study phone so participants will not bear a cost burden.

If no COVID-19 exposure or symptoms are reported, enumerators will administer a questionnaire to record any other morbidity symptoms or adverse events experienced by the index participants during the 2 weeks since the previous home visit. Index participants will also be requested to report any symptoms or adverse events (e.g., hospitalizations) experienced by other household members. In the case of symptoms experienced by other household members, limited descriptive information (such as whether the individual is an adult or child) will be recorded, but no identifying information (name, age, etc.) will be obtained for the individual.

To monitor adherence, at each biweekly visit, enumerators will then record an inventory of all bouillon cubes in the household (study-provided and commercial). Households will be requested to save wrappers from all bouillon used in the household (i.e., study-provided bouillon and any

commercial bouillon), which will be counted at each visit. The enumerator will also administer a brief questionnaire about household bouillon use **and recent purchases** at each visit, as well as exclusion criteria (self-reported pregnancy among WRA or lactating women, or discontinuation of breastfeeding among lactating women).

Additional data collection that will take place **every 4 weeks** during the intervention period will include: 1) assessment of household food security **and water security**, 2) breastfeeding status of enrolled children 2-5 years of age, and 3) dietary assessment among all enrolled participants **(brief food frequency questionnaire)**.

Endline visits

At the end of the intervention period, participants will be invited to the biospecimen collection site for the two endline data collection visits. As previously mentioned, replicate endline assessments will be conducted for some outcomes to increase statistical power. The duration of the intervention period will be 38 weeks for WRA (36-44 weeks allowable, considering scheduling logistics and potential deferrals), 38 weeks for children (36-42 weeks), and 12 weeks (11-15 weeks) in the case of households in which a lactating woman is the only participant). For blood samples, the endline assessments will be scheduled for 35 weeks and 38 weeks and for breast milk samples, the endline assessments will be scheduled for 11 weeks and 12 weeks. Delivery of study bouillon cubes will continue through the last endline assessment for each household.

Data collection procedures at the first endline visit will be similar to those described at the baseline visit. Briefly, data collection will include anthropometric measurements, morbidity symptoms, and spot urine samples (all groups), blood pressure measurement and venous blood collection (WRA and children), breast milk collection (lactating women), and stool collection and developmental assessments (children). For WRA, the amount of blood collected at the first endline visit will be lower (up to 8 ml) compared to the baseline and Endline 2 visit (up to 12 ml), considering that the vitamin A isotopes will not be measured in the Endline 1 sample. **However, we will collect up to 12 ml of blood in a randomly selected subset of women (n=50/group) at the Endline 1 visit to measure the labelled vitamin A. These results will be needed to inform the TBS assessments in the Endline 2 sample.**

In addition, at the first endline visit (35 wk), WRA will receive the oral vitamin A isotope dose to enable measurement of total body vitamin A stores at endline. **If we need to** use a different stable isotope ($^{13}C_{10}$-labelled vitamin A) **then we will submit safety documentation for this isotope to the Ghana Food and Drugs Authority for approval**. **Results from our RID pilot study will allow us to determine whether we need to use $^{13}C_{10}$-retinyl acetate at endline (for example, if we learn that the baseline dose of labelled vitamin A could potentially affect endline assessment of total body vitamin A stores if the same isotope is used). (Both d6-retinyl acetate and $^{13}C_{10}$-retinyl acetate are sourced from the company Buchem BV (Apeldorn, Netherlands), certified as food grade (Food Chemical Codex IV)**. To assess eligibility for the dose, participants will undergo screening procedures identical to those used prior to isotope dosing at baseline (described above). These include questionnaires to assess morbidity symptoms and vitamin A intake, a spot urine sample to assess pregnancy, and a venous blood sample to assess anaemia, malaria infection and inflammation. Similar to baseline, individuals who are pregnant **will be excluded from the study. Individuals** who have severe anaemia will be excluded from receiving the vitamin A isotope dose **and from blood collection**

at the endline 2 visit. They will be referred for medical evaluation but will not be excluded from the study. WRA with CRP > 5 mg/L or a positive malaria RDT (or who meet other deferral criteria) will not receive the vitamin A isotope dose on this day; these individuals will defer the isotope dosing until the second endline visit (3 weeks later).

Endline dietary data collection will be conducted by administering the 24-hour dietary recall interview during two home visits on non-consecutive days during the 30 days prior to Endline 2. **In each household, enumerators will also repeat the baseline assessment of hypothetical willingness to pay for a new fortified bouillon cube.** A single endline child development assessment (administration of the HOME, MDAT **and EYT**) will be completed during a home visit **or a visit to a central location** scheduled between the two endline visits (35wk and 38wk).

The second endline assessment will be scheduled approximately 3 weeks after the first endline assessment (38 wk). Data collection procedures at the second endline assessment will include a subset of the outcomes measured at the first endline assessment, measured using identical methods. These will include morbidity symptoms (all groups), venous blood collection (WRA and children) and breast milk collection (lactating women). WRA who defer the vitamin A isotope dose after the first endline will be screened using the same procedures for eligibility to receive the isotope dose at the second endline. WRA who are eligible to receive the isotope dose at the second endline will be asked to return for an additional follow-up venous blood sample (up to 6ml) 3 weeks later.

The study exit form will be completed at the second endline visit (or final venous blood sample in the case of WRA with a deferred second endline visit), or at a final subsequent home visit according to participant convenience. At this time, all remaining study bouillon cubes will be collected from the household.

Data collection methods

Household information

Socio-economic status (SES) and household characteristics: Information on demographic and SES **characteristics of the household and index participants** will be collected at the time of the recruitment in-home visit **and during the observation period**. Demographic and socio-economic information will be obtained by using structured interviews (household demographic composition; age, education and employment status of household members; material possessions and assets), inspection of housing quality (construction materials for roof, walls, and floor; sanitary facilities) and availability of services (water, sewerage, electricity, and distance to road, market and clinic).

Food and water security: Assessment of household food security will be based on the USAID FANTA 360 Household Food Insecurity Access Scale (HFIAS) and will be conducted every 4 weeks throughout the **intervention period**. Water insecurity will be assessed every 4 weeks using the Household Water InSecurity Experience (HWISE) scale.

Adherence

At the time of the initial home visit after randomization and at each biweekly household visit, enumerators will record an inventory of all bouillon cubes in the household (study-provided and

commercial). Households will be requested to save wrappers from all bouillon consumed during the 2-week period (study and non-study cubes), which will be counted at each visit. The enumerator will also administer a brief questionnaire about household bouillon **purchase and** use at each visit, including sharing of study bouillon with non-household members. Households with sustained low adherence will be identified through routine monitoring, and enumerators will be instructed to inquire about the reasons for low adherence and to encourage use of the study bouillon.

Morbidity

Enumerators will visit each home in their assigned communities on a biweekly basis during the intervention period to assess morbidity (i.e., every two weeks). Information will be obtained systematically on the presence and severity of selected symptoms of pre-defined diseases for each day of the preceding two weeks. Specifically, the enumerators will inquire about stool number and consistency, the presence of fever, vomiting, cough, nasal discharge, skin rash, depressed appetite, and any other symptoms of concern to the participant (or caregiver). Temperature will be measured at every home visit.

Women of reproductive age and lactating women will be asked (privately/discreetly) at each biweekly visit if they are currently pregnant (self-report).

Index participants will also be requested to report any adverse events (e.g., hospitalizations) experienced by other household members. In such cases, limited descriptive information (such as whether the individual is an adult or child) will be recorded, but no identifying information (name, age, etc.) will be obtained for the individual.

Diarrhoea will be defined as the presence of three or more loose or liquid stools per 24 hours, and upper respiratory infection as the presence of cough and purulent nasal discharge. The enumerator will ascertain whether the study participant sought medical treatment (from any sources) and what treatments was provided to the study participant.

If at the time of the biweekly household visit, the enumerator learns that a study participant has symptoms of illness (e.g., diarrhoea, malaria), the study participant will be referred to the local health centre or district hospital for evaluation. Study participants with any danger signs (altered consciousness, seizures, refusal to eat or drink, or vomiting of everything consumed) will be referred immediately to the local health centre or district hospital**.** If the study participant needs transportation to have the first contact with the health centre or hospital, the project will provide the transportation.

Anthropometry

Weight of women and children will be measured in duplicate to the nearest 0.1 kg using an electronic scale (Seca 874). If the 2 measurements differ by more than 0.5 kg, a 3rd measurement will be taken and the closest 2 measurements retained for data analysis.

Standing height of women and children will be measured in duplicate to the nearest 0.1 cm using a stadiometer (Seca 217). If the 2 measurements differ by more than 0.5 cm, a 3rd measurement will be taken and the closest 2 measurements retained for data analysis. Participants will be

asked to remove shoes and hats or head coverings prior to measurement. In the case of hair or head coverings that cannot be removed or compressed, the height of the hair will be estimated separately and subtracted from the height measurement.

Mid-upper arm circumference will be measured in duplicate among children to the nearest 0.1 cm using a Shorr Child MUAC Tape measuring tape. If 2 measurements differ by more than 0.5 cm, a 3rd measurement will be taken and the closest 2 measurements retained for data analysis.

Waist and hip circumference of women will be measured in duplicate to the nearest 0.1 cm using a nonflexible measuring tape. If 2 measurements differ by more than 0.5 cm, a 3rd measurement will be taken and the closest 2 measurements retained for data analysis. Waist circumference will be measured over no or light clothing at the midpoint between the iliac crest and lowest rib. Hip circumference will be measured over light clothing at the widest (maximum) hip circumference.

Dietary intake

24 hour recalls

Quantitative dietary intake data will be collected through 24-hour dietary recall interviews **during the observation period, and** at baseline and endline. For each participant, two dietary recalls will be conducted on non-consecutive days during the first 4 weeks of the intervention period to capture usual bouillon and nutrient intake early in the study. In addition, two recalls will be collected on non-consecutive days during the last 4 weeks of the trial, to assess usual bouillon intake at the end of the intervention period. Collection of dietary intake data at these ‘early’ and ‘late’ periods will enable comparison of whether there were groupwise differences in change in mean bouillon or nutrient consumption over the course of the study. Due to the shorter study duration for lactating women, only the 2 baseline dietary recalls will be collected for lactating women. **Comparison of dietary data from the observation period and the baseline and endline time points will allow us to determine the extent to which bouillon intake changed and whether this change differed by intervention group.**

Dietary recalls will be conducted by study staff with extensive training and practice on the 24 hour recall technique. Interviews will follow a multiple pass methodology to collect information on all foods and beverages consumed during a defined 24 hour period. Because eating from a common pot is common in the study area, women (and caregivers of children) will be notified of the interview day in advance and requested to use an individual plate or bowls on the index day (e.g., the day before the 24 hour recall interview to assist in visualizing portion sizes consumed. For children 2-5 years of age, the respondent may consult informally with multiple household members to help them answer the questions and obtain information about all foods or beverages consumed by the child during the index time period. Recall data will be collected using a tablet-based data collection system (an adaptation of OpenDRS) that allows for direct entry of dietary data on the tablet, through a platform that guides interviewers through the dietary recall interview and suggests prompts to aid in the interview process. Where recipe data already exist, enumerators will ask for descriptive information on the recipe (e.g., main ingredients) but will not collect the full recipe information (since recipe data will have already been collected during pilot work in the study area). However, for recipes not already collected, **recipes containing bouillon,** or for which there is large inter-household variation in recipe composition, the interview will also include collection of all information about the recipe, including types and amounts of ingredients, cooking method, and total yield.

Dietary recalls will be conducted in participant households to take advantage of the opportunity to use the participants' typical utensils for portion size estimation where possible. Enumerators will also carry "kits" with common utensils and foods (e.g., the staple food TZ, dried leaves, spices, etc.) to obtain weights for portions and ingredients where possible.

Other dietary intake data
Information on breastfeeding practices, dietary diversity, and consumption of specific foods and food groups (including bouillon) will be assessed at **the initial household visit**, endline, and every 4 weeks throughout the study.

Among children 2-5 y, baseline assessments will include whether a child was ever breastfed and reported continued breastfeeding at 2 years of age. In addition, for children who are still breastfeeding at enrolment, questionnaires administered every 4 weeks will be used to determine whether the child continued breastfeeding during each interval.

Questions regarding breastfeeding practices will also be asked of lactating women (children 4-18 months of age at baseline).

Brief food frequency questionnaires covering the period of the previous 24 hours will be administered to all participants during the home visit to assess dietary diversity. Minimum dietary diversity for women (MDD-W) will be calculated for women of reproductive age and lactating women. Selected foods/food groups will also be assessed over a **7-day or 14**-day recall period.

Blood sample collection and processing

Study participants will be assessed for indicators of haematological, inflammation and micronutrient status.

All biological sample collection will be conducted by trained phlebotomy or laboratory technicians. Standard procedures will be followed to ensure proper handling of biological samples and disposal of biological waste. Technicians will wear lab coats and disposable gloves and will change gloves between participants.

Samples will be collected at a central location in the community in the early morning. We will create mobile or temporary biospecimen collection sites as was done in the Ghana Micronutrient Survey and the pilot survey for this project, so that participants will not have to travel long distances to have their samples collected. At each temporary biospecimen collection site, we will ensure that our mode of operation is consistent with the Ghana Health Service's COVID-19 mitigation measures. We will bring along enough supplies, including a non-contact infra-red thermometer, tables and chairs, as well as personal protective equipment (PPE) such as masks, hand-washing stations, and hand sanitizers.

Trained phlebotomists will collect venous blood (up to 12 ml, or ~2.5 teaspoons, from WRA or up to 7.5 ml, or ~ 1.5 teaspoons, from children) from the antecubital vein for measurement of nutritional biomarkers. This quantity is in accordance with the World Health Organization and the University of California, Davis guidelines for minimal risk among young children (< 3 ml/kg body weight/8 week period). The amount of blood to collect was calculated by estimating the

amount of blood required for each of the micronutrient biomarkers listed in Table 1 (biomarkers of vitamin A, zinc, iron, vitamin B-12, and folate status). The quantity of blood samples is greater for WRA compared to children because of the additional volume of specimen required for the retinol isotope dilution test and erythrocyte folate measurement, which will not be conducted among children. For a complex intervention such as ours, a range of outcome measures is necessary to make best use of the data and provide an adequate assessment of the success or otherwise of the intervention, which may have effects across several domains. Blood will be collected into vacutainer tubes and placed immediately into a battery-operated cold box or cooler containing cold packs until centrifugation.

If participants are acutely ill with fever (or other symptoms as described above under Exclusion/Deferral criteria) on the day of a scheduled blood drawing or report these symptoms in the 72 hours prior to the blood draw, the sample collection will be postponed for 2 weeks (baseline visit) or 3 weeks (endline visit). Information necessary to interpret biomarker values, such as the time of the participant's most recent meal, will be collected at the time of the blood sample. WRA, but not children, will be asked to fast for at least 8 hours prior to their blood draw appointment.

a. Depending on timepoint (see **Table 6**), hemoglobin concentrations will be measured in capillary or venous whole blood immediately after collection. For the two RID screening/dosing time points for WRA, haemoglobin and CRP concentrations will be measured by the QuikRead go instrument (Aidian Oy, Espoo, Finland). At baseline and endline**,** hemoglobin will be measured using a portable photometer (HemoCue, Inc.). In addition, haemoglobin and zinc protoporphyrin (ZPP) will be measured in the same blood sample using a point-of-care device (iCheck Anemia). At each time point, malaria infection will be assessed with a rapid diagnostic test (RDT). At time points which specify a venous collection, if the phlebotomist is unable to obtain a venous blood sample after 2 attempts, a capillary sample will be obtained by finger stick for measurement of **micronutrients/acute phase proteins (MN/APP, measured by ELISA),** hemoglobin**,** CRP and malaria, as needed. The results of the analyses for hemoglobin and the malaria RDT will be explained verbally to the participant, and a written copy of the results will be provided. Participants diagnosed with un-treated malaria will be referred to the nearest health centre for further evaluation. WRA and children with severe anaemia at screening and/or baseline (Hb < 80 g/L for non-pregnant women or < 70 g/L for children) will be excluded from participating in the trial, per established inclusion/exclusion criteria and will be referred to Tamale West Hospital. WRA and children with severe anaemic at endline 1 (Hb < 80 g/L for non-pregnant women or < 70 g/L for children) will be referred to Tamale West Hospital. They may remain in the trial, but will not receive the vitamin A isotope dose (WRA), and blood samples at endline 2 will not be collected. Participants diagnosed with any anaemia (Hb < 120 g/L for non-pregnant women or < 110 g/L for children) at endline 2 will be referred to the nearest health centre for medical evaluation.

b. Serum **and/or plasma** will be separated within 8 hours after blood collection by centrifuging blood samples. **Samples** will be aliquoted into polypropylene cryovials

for measurement of indicators of micronutrient status, such as vitamins A and E, minerals (e.g., iron and zinc), and B vitamins (e.g., B9 and vitamin B12). In addition, indicators of systemic inflammation (such as C-reactive protein, CRP, and alpha-1-acid glycoprotein, AGP) will be measured to assist in interpretation of the micronutrient biomarkers. **Among WRA only, additional sample will be aliquoted into polypropylene cryovials for measurement of vitamin A isotope concentrations.**

c. Among WRA (but not children), whole blood hemolysates will be prepared using an ascorbic acid solution and then aliquoted into polypropylene cryovials for measurement of folate concentration (to calculate erythrocyte folate). Haematocrit will also be measured among women for the assessment of erythrocyte folate.

All samples will be transported from the biospecimen collection site to the District Hospital at Tolon (see enclosed letter of support) daily for storage in a -86 ºC freezer, with regular temperature monitoring. Samples thus stored will be periodically transported to the University of Ghana for a more permanent storage before analysis.

Table 6. Schedule for blood collection.

**Participants**	**Screening for RID isotope dosing 1 (Finger-prick blood)**	**Baseline (Venous blood)***	**Intervention period**	**Endline 1 (RID isotope dosing 2) (Venous blood)***	**Endline 2 (Venous blood)***
**WRA**	Hb, CRP, RDT	Hb, Hct, RDT, ZPP, MN/APP, pZn (minerals), retinol/vitamin A isotopes, vitamin E, B vitamins	--	Hb, Hct, RDT, CRP, ZPP, MN/APP, pZn (minerals), retinol, vitamin E, B vitamins,	Hb, Hct, RDT, ZPP, MN/APP, pZn (minerals), retinol/vitamin A isotopes, vitamin E, B vitamins, RBC folate
**Children**	--	Hb, RDT, ZPP, MN/APP, pZn (minerals), retinol, vitamin E, B vitamins	--	Hb, RDT, ZPP, MN/APP, pZn (minerals), retinol, vitamin E, B vitamins	Hb, RDT, ZPP, MN/APP, pZn (minerals), retinol, vitamin E, B vitamins
**Lactating women**	--	--	--	--	--

Hb, haemoglobin; Hct, haematocrit; CRP, C-reactive protein; MN/APP, micronutrient/acute phase protein ELISA panel conducted by the Vit-Min lab; pZn, plasma zinc (analysis includes a suite of minerals, including zinc and iron), RBC, red blood cell; RDT, rapid diagnostic test (for malaria)**;** WRA, women of reproductive age; ZPP, zinc protoporphyrin

* At time points which specify a venous collection, if the phlebotomist is unable to obtain a venous blood sample after 2 attempts, a capillary sample will be obtained by finger stick for measurement of **micronutrients/acute phase proteins (MN/APP),** hemoglobin, CRP and malaria, as needed.
Note: If RID pre-endline isotope dose is deferred (for example, due to positive malaria RDT; see deferral criteria above), then eligibility for isotope dosing will be assessed again at Endline 2; if the woman is eligible for isotope dosing at Endline 2, then an additional venous blood sample will be collected 3 weeks later, with a quantity sufficient only for retinol/vitamin A isotopes and MN/APP measurement. If a WRA receives the pre-endline dose at 35 weeks as planned and then has to defer the week 38 visit, the deferred Endline 2 blood sample at 41 weeks will be used for the RID measurement as well as the other micronutrient biomarkers.

Breast milk sample collection and processing

Breast milk samples will be collected from lactating women for measurement of concentrations of micronutrients, such as vitamins A and E and B vitamins (e.g., B-12). Breast milk samples will be collected using the "full milk sample" methodology. Breast milk will be collected from the breast from which the infant has not fed for a longer time (i.e., the breast that is 'more full'), with a minimum of one hour elapsed between the milk sample collection and previous feeding. Time of day and time since a previous feed will be recorded. A trained member of the study team will assist the woman in using a breast pump to collect all milk from a single breast. A ~10ml sample of well-mixed milk will be retained for measurement of milk fat and vitamins. The remainder of the milk will be returned to the mother to be fed to the infant using a cup and spoon (avoiding bottle use). The amount of milk retained for the study (~10ml) is small relative to an infant's daily intake (<2% for infants < 12 months of age) and unlikely to affect breastmilk intake of the breastfeeding infant. Breast pump use is likely to be uncommon in the study site, but these procedures have been used successfully in studies in similar populations. Before collecting the sample, the study staff member will explain how the breast pump works and ensure that the mother is comfortable with the procedure and has the opportunity to have any questions answered. The collection will be done in a private area.

Milk fat content will be measured in fresh milk at the time of sample collection using the "Creamatocrit" method (Lucas et al., 1978; Engle-Stone et al, J Nutr, 2017). This method involves centrifuging the milk to separate the fat and can be conducted using a standard centrifuge and callipers or a specialized "Creamatocrit" centrifuge. The 'Creamatocrit' centrifuge is similar to a standard centrifuge but is pre-calibrated with the appropriate centrifugation speed for milk samples and includes a built-in 'calliper' that records the length of the fat and total milk samples in the tube. The method does not require any additional chemicals or reagents. To apply this method, milk samples will be collected in microhematocrit tubes and centrifuged to separate the aqueous and lipid layers, and the length of each layer will be measured to calculate lipid content expressed as % of volume, which will be converted to lipid content in g/L using a validated regression equation.

As for blood, milk samples will be transported from the mobile biospecimen collection site to the District Hospital at Tolon daily for storage in a -86 ºC freezer, with regular temperature monitoring. Samples thus stored will be periodically transported to the University of Ghana for a more permanent storage before analysis. Breast milk samples will be shipped on dry ice to UC

Davis for analysis after study completion. Milk samples will be analysed to measure concentrations of vitamins A and E, as well as B vitamins.

Urine collection and processing

A spot urine sample will be collected at **screening,** baseline and endline **1** from non-pregnant, non-lactating women, and **at baseline and endline 1 from** children, for measurement of iodine and sodium concentrations. Urinary creatinine and potassium will also be measured to interpret the sodium concentrations. Proper precautions will be used to avoid iodine contamination. Samples will be aliquoted at the mobile biospecimen collection site and placed in a cool box and transported to the study laboratory, where they will be stored at -20°C.

Among WRA only, spot urine samples collected on the day of screening and the pre-endline isotope dosing will **also** be used to assess pregnancy status based on rapid tests for human chorionic gonadotropic (HCG).

Stool collection (children only)

Stool samples will be collected from children to measure faecal calprotectin, as a marker of intestinal inflammation (a safety measure). Calprotectin in stool is considered stable for at least 3 days in room temperature (Tøn et al. (2000) Clin Chim Acta), but we will attempt to refrigerate stool within 12 hours of collection and freeze within 24 hours of collection. **In addition, in a randomly selected subset of participants, an aliquot of stool will be transferred to a separate collection tube for characterization of the gut microbiome.**

During the **baseline observation** period **(and again during the last biweekly visit prior to endline)**, the child's caregiver will be provided with a labelled stool container, a **portable potty and/or** diaper **in** which to collect the stool, gloves, **a** plastic spoon, **2 specimen collection containers** and 2 plastic bags. The caregivers will be **reminded** one day before the planned stool collection (baseline and endline) and requested to collect the first available fresh stool sample from the child on the morning of the planned visit to the central mobile biospecimen collection site. The caregiver will be instructed to use the plastic spoon to collect 3-4 spoons of stool and place it in the stool container, close the lid tightly and place the container in the plastic bag. **For the subset of children selected for microbiome analysis, the caregiver will also use the provided scoop to collect a small sample of stool into the microbiome analysis tube. If the child does not pass stool in the morning prior to visiting the biospecimen collection site, the caregiver will be instructed to keep the collection materials and collect the stool samples later in the same day. When the stool sample is collected the caregiver may either call the enumerator to come pick up the stool sample, or the enumerator may collect the sample when they conduct the initial visit to the household the following day.**

Stool samples will only be stored and analysed from children who are eligible to participate in the study at baseline and randomized to a treatment group.

Follow-up of women exiting the study after self-reporting or testing positive for pregnancy

Among non-pregnant, non-lactating women of reproductive age (WRA) participants, pregnancy during the intervention may be determined via self-report assessment at any point

during the intervention or during the scheduled pregnancy testing at the Endline Visit #1. Among lactating women, pregnancy during the intervention may be determined via self-report assessment at any point during the intervention.

Some of the women exiting the study after self-reporting or testing positive for pregnancy may travel elsewhere for family or economic reasons or go to extended family members (e.g., mothers, in-laws, etc.) elsewhere just to deliver their babies, which is a common practice in Ghana. Therefore, using telephone calls would be the most practical and efficient way to follow-up the pregnant women once they have exited the study. We will obtain at least two phone numbers for each pregnant woman exiting the study after self-reporting or testing positive for pregnancy, for subsequent follow-up contacts until one month after child delivery. If a pregnant woman does not have access to a phone, we will provide one and ask her to make sure she can receive our scheduled calls.

Enumerators will contact pregnant women exiting the study bimonthly by phone and interview them about the occurrence of any of the following: (a) miscarriage, (b) still birth, and (c) congenital abnormality. These data will be reported by intervention group.

Biological sample storage, transport, and analysis

Storage and transport

All samples will be transported from the mobile biospecimen collection site to the District Hospital at Tolon daily for storage in a -86 ºC freezer, with regular temperature monitoring. Samples thus stored will be periodically transported to the University of Ghana for longer-term frozen storage before analysis. Samples to be analysed outside Ghana will be shipped by air on dry ice to the respective labs (as described below).

Laboratory analysis

Indicators of iron (ferritin, soluble transferrin receptor) and vitamin A status (retinol binding protein) and inflammation (CRP and AGP) will be measured using a sandwich ELISA technique at the Vit-Min Laboratory (Willsteadt, Germany). Serum concentrations of d6-retinol and 12C-retinol will be measured by LC/MS/MS at Newcastle University (Newcastle, UK). Breast milk and serum/**plasma** vitamin A and E will be measured by high performance liquid chromatography (HPLC) at **the University of California** (vitamin A is the primary analyte of interest, but data for vitamin E can be generated in the same analytical run). **Plasma** mineral concentrations (e.g., zinc and iron) will be measured by ICP-OES at the University of California. Additional markers of inflammation (amyloid A, MCP-1, IL-6, IL-10, IL1beta, and adiponectin) will be measured by ELISA at the University of California. B vitamin concentrations in serum/**plasma** and breast milk will be measured on the University of California, Davis campus at the Western Human Nutrition Research Center (WHNRC) building. Analyses will focus on folate (B9) and vitamin B12 concentrations as the primary B vitamins of interest. Erythrocyte **and serum/plasma** folate concentrations will be measured at the US CDC (Atlanta, Georgia). Urinary iodine concentration will be assessed using a modification of the Sandell-Kolthoff reaction at the University of Ghana. Urinary sodium and potassium will also be measured at the University of Ghana. Urinary creatinine will be measured for interpretation of urinary sodium concentrations by the Jaffe reaction using a commercially available kit and autoanalyzer at the University of California. Stool calprotectin will be analysed using a commercially available kit **and gut microbiota will be characterized by bacterial 16S rRNA sequencing** at the

University of California. Appropriate standard reference materials and quality control standards will be included in and analysed with all sample runs. Novel indicators and other indicators of micronutrient status, growth and infection may be assessed if funding becomes available.

Laboratory competency

Table 7 describes relevant publications and validation studies illustrating the competency of each of the laboratories listed above for performing the proposed analyses.

Table 7. Laboratory site and evidence of competency for biospecimen and bouillon cube lab analyses.

Institution	Analysis	Citation(s)	Notes
University of Ghana	Urinary iodine	National iodine survey, Ghana, 2015<br><br>Certificate of successful participation in the EQUIP program (Ensuring the Quality of Urinary Iodine Procedures)	National survey report and certificate of successful participation in global quality assurance program (US CDC)
University of Ghana	Vitamin A in bouillon	Rapid measurement of iron, iodine, and vitamin A in bouillon with iChecks. BioAnalyt Application paper, October 2019.	Manufacturer-provided summary of method validation.
University of Ghana, Ecological Laboratory (ECOLAB).	Urinary sodium and potassium	Fosu-Mensah et al. Organophosphorus pesticide residues in soils and drinking water sources from cocoa producing areas in Ghana. Environ Syst Res (2016) 5:10<br>DOI 10.1186/s40068-016-0063-4	Peer-reviewed paper demonstrating application of methodology for analysis of urinary sodium.
University of California	**Plasma** minerals (e.g., zinc and iron)**;** minerals in bouillon cubes	Wessells et al. Plasma zinc concentration responds rapidly to the initiation and discontinuation of short-term zinc supplementation in healthy men. Journal of Nutrition. 2010 Dec; 140(12): 2128-2133.<br><br>Engle-Stone R, Nankap M, Ndjebayi AO, Allen LH, Shahab-Ferdows S, Hampel D, Killilea DW, Gimou MM, Houghton LA, Friedman A, Tarini A, Stamm RA, Brown KH. Iron, zinc, folate, and vitamin B12 status increased among women and children in Yaounde and Douala, Cameroon, 1 year after introducing fortified wheat flour. J Nutr. 2017 Jul; 147(7):1426-1436.	Peer-reviewed articles demonstrating application of methodology, including quality control data
	Serum/**plasma** and breast milk vitamin A **and E concentratio**	Engle-Stone R, Nankap M, Ndjebayi A, Gimou MM, Friedman A, Haskell MJ, Tarini A, Brown KH. Vitamin A status of women and children in Yaoundé and Douala, Cameroon, is unchanged one	Peer-reviewed articles demonstrating application of methodology, including quality

	**n**	year after initiation of a national vitamin A oil fortification. Nutrients. 2017 May 20; 9(5).<br><br>Palmer AC, Chileshe J, Hall AG, Barffour MA, Molobeka N, West KP Jr, Haskell MJ. Short-term daily consumption of provitamin A carotenoid-biofortified maize has limited impact on breast milk retinol concentrations in Zambian women enrolled in a randomized controlled feeding trial. J Nutr 2016 sep; 146(9):1783-92.	control data
	B vitamins in blood/plasma	Engle-Stone R, Nankap M, Ndjebayi AO, Allen LH, Shahab-Ferdows S, Hampel D, Killilea DW, Gimou MM, Houghton LA, Friedman A, Tarini A, Stamm RA, Brown KH. Iron, zinc, folate, and vitamin B12 status increased among women and children in Yaounde and Douala, Cameroon, 1 year after introducing fortified wheat flour. J Nutr. 2017 Jul; 147(7):1426-1436.	Peer-reviewed articles demonstrating application of methodology, including quality control data
	Breast milk vitamin B12 **concentration**	Hampel D, Dror DK, Allen LH. Micronutrient in human milk: Analytical methods. Adv Nutr. 2018 May 1; 9(suppl_1):313S-331S.	Peer-reviewed article describing method validation, and review of methodological issues
	Markers of inflammation (amyloid A, MCP-1, IL-6, IL-10, IL1beta, and adiponectin)	Butler AA, Price CA, Graham JL, Stanhope KL, King S, Hung YH, Sethupathy P, Wong S, Hamilton J, Krauss RM, Bremer AA, Havel PJ. Fructose-induced hypertriglyceridemia in rhesus macaques is attenuated with fish oil or ApoC3 RNA interference. J Lipid Res. 2019 Apr; 60(4):805-818.	Peer-reviewed article demonstrated application of methodology, including quality control data
	Urinary creatinine	Wessells KR, Hess S, Rouamba N, Ouédraogo Z, Kellogg M, Goto R, Duggan C, Ouédraogo J, Brown KH. Associations between intestinal mucosal function and changes in plasma zinc concentration following zinc supplementation. J Pediatr Gastroenterol Nutr, 2013; 57(3):348-55.	Peer-reviewed article demonstrated application of methodology, including quality control data
	Stool calprotectin	Hinnouho G-M, Wessells KR, Barffour MA, Sayasone S, Arnold CD, Kounnavong S, Hess SY. Impact of different strategies for delivering supplemental zinc on selected fecal markers of environmental enteric dysfunction among young Laotian children: A randomized controlled	Peer-reviewed article demonstrated application of methodology, including quality control data

		trial. Am J Trop Med Hyg. 2020 Oct; 103(4):1416-1426.	
	**Gut microbiota**	**Hughes RL, Arnold CD, Young RR, Ashorn P, Maleta K, Fan Y-M, Ashorn U, Chaima D, Malamba-Banda C, Kable ME, Dewey KG. Infant gut microbiota characteristics generally do not modify the effects of lipid-based nutrient supplementation on growth or inflammation: secondary analysis of a randomized controlled trial in Malawi.**	**Peer-reviewed article demonstrated application of methodology, including quality control data**
CDC	RBC**, whole blood, and serum** folate	Zhang M, Sternberg MR, Yeung LF, Pfeiffer CM. Population RBC folate concentrations can be accurately estimated from measured whole blood folate, measured hemoglobin, and predicted serum folate – cross-sectional data from the NHANES 1988-2010. Am J Clin Nutr. 2020 Mar 1; 111(3):601-612.	Peer-reviewed article demonstrated application of methodology, including quality control data
Vit-Min lab	ELISA analysis of biomarkers of iron (ferritin, transferrin receptor) and vitamin A status (retinol-binding protein), and markers of inflammation (CRP, AGP)	Erhardt JG, Estes JE, Pfeiffer CM,Biesalski HK, Craft NE. Combinedmeasurement of ferritin, soluble transferrin receptor, retinol binding protein, and C-reactive protein by an inexpensive, sensitive and simple sandwich enzyme-linked immunosorbent assay technique. J Nutr.2004 Nov; 134(11):3127-32.	Peer-reviewed article describing method validation.
Newcastle University	d6-retinol and 12-C retinol in serum and dried serum spots	Green MH, ford JL, Green JB, Berry P, Boddy AV, Oxley A Lietz G. A retinol isotope dilution equation predicts both group and individual total body vitaminA stores in adults based on data from anearly postdosing blood sample. J Nutr. 2016 Oct; 146910):2137-2142.	Peer-reviewed article providing detailed description of retinol isotope dilution methodology, including lab analysis.

7. Sample size

The primary sample size calculation for the trial is based on detecting a difference in micronutrient status indicators of 0.26 standard deviations or more (see justification below), assuming level of significance of alpha=0.05 and 80% power, and a single baseline and single endline sample for each primary outcome. This would require a sample size of n=234 per group for a 2-arm trial. This

sample size would also allow us to detect a decrease of >13 percentage points in the prevalence of micronutrient deficiency, assuming a conservative overall prevalence of 50%. A higher or lower overall prevalence of deficiency would power us to detect smaller percentage point changes.

Assuming 15% attrition after randomization (e.g., due to withdrawal of consent, loss to follow up, or missing blood samples), a total of n=276 participants per intervention group will be required at baseline, or n=552 in total per target group for children 2-5 y and lactating women. For WRA, we assume an attrition rate of 30% after randomization to account for participant exclusion due to pregnancy (given that fertility rates are high in the area), which would require 334 participants per intervention group, or n=668 total at baseline.

Based on pilot data, we further estimated the target number of participants to enrol in order to achieve the expected sample size after randomization. These estimates are 20% for lactating women and children 2-5 y of age, and 35% for non-pregnant, non-lactating women (given the additional exclusion criteria applied). Therefore, the target enrolment for each group will be: 690 lactating women, 690 children 2-5 y of age, and 1028 non-pregnant, non-lactating women. We will monitor enrolment and attrition rates, with a focus on attrition prior to randomization, to determine whether any changes in recruitment targets are necessary.

7.1 Repeated endline samples

Repeated endline samples will be collected for selected biomarker (as described above) to increase the statistical power to detect an effect size of 0.26 or larger. Simulations conducted for this project by Mediana indicate that addition of a second endline measure would increase the statistical power to detect this effect size from 80% to 90-100%, depending on the correlations between endline samples and between baseline and endline samples. Considering we may observe smaller than expected effect sizes or have larger than expected attrition, adding an additional endline measure is a cost-effective way to ensure the study will be properly powered to detect the intervention effect.

7.2 Rationale for target effect size

The effect size of 0.26 was based on calculations of the change in total body vitamin A stores among women (assuming 9-month trial duration and total vitamin A intake from bouillon cube of 500 micrograms vitamin A per day). We also estimated the change in body iron stores among children and women (assuming 9 month trial duration, 4 mg iron per gram of cube, and bouillon cube intake of 1.0, and 2.5 grams per day, respectively). For iron, the calculations assume that cubes are fortified using ferric pyrophosphate, which we assumed would have low absorption of ~2%. Greater absorption would be expected to reduce the sample size required to detect an effect on iron status.

For the indicator ferritin, an increase of 0.26 standard deviations would roughly translate into an increase of 9.1-10.4 µg/L, assuming a standard deviation of 35 µg/L or 40 µg/L, based on results for children and WRA, respectively, in the pilot survey. For RBP as a marker of vitamin A status among children, an effect size of 0.26 would translate into an increase of ~0.05 µmol/L RBP concentrations, based on the standard deviation of 0.19 µmol/L observed in the pilot study (for

women, this would translate into an increase of ~0.10 µmol/L, based on an observed SD of 0.39 for RBP).

Regarding serum or plasma zinc as an indicator of zinc status, a Cochrane review on zinc fortification reported that zinc fortification in combination with other micronutrients may not increase plasma zinc concentration, but that foods fortified with zinc in comparison to foods without added zinc increased plasma zinc by 2.12 µmol/L (95% CI: 1.25-3.00 µmol/L) (Shah et al., 2016). **Additionally, a review and meta-analysis by Tsang and colleagues found that zinc fortification with and without multiple micronutrients increased plasma zinc concentrations (4.68 µg/dL, 95% CI: 2.62-6.75; equivalent to 0.72 µmol/L, 95% CI: 0.40-1.03 µmol/L).** Based on the SD of plasma zinc concentrations observed in the pilot survey (1.32 µmol/L among children and 1.48 µmol/L among WRA), an effect size of 0.26 would translate to an increase of ~0.34-38 µmol/L in mean plasma zinc concentration.

We expect that the target sample size set for iron and vitamin A biomarkers will be sufficient to detect a change in indicators of folate and vitamin B12 status, for which we expect to observe a larger change (effect size ≥ 0.5; Engle-Stone et al., J Nutr, 2017; Crider et al., 2019). Based on plasma B12 concentrations in the pilot survey (SD of plasma B12 was 195 pmol/L for children and 223 pmol/L for WRA), we could detect a minimum change of 51 pmol/L for children and 58 pmol/L for WRA. At the planned sample size, we would be able to detect an increase of at least 13 percentage points (assuming a worst case scenario of 50% baseline prevalence) in the prevalence of RBC folate concentrations above 906 nmol/L, the threshold associated with minimal neural tube defect risk (WHO, 2020).

<u>Opinions, attitude, and acceptance (OAA) towards the study bouillon among adult household members</u>

We will use a mixed methods approach (quantitative survey and focus group discussions) to collect data on the opinions, attitude, and acceptance towards the study-supplied bouillon cubes among adult household members of the women and children enrolled in the randomized trial.

i. Participants

The quantitative survey will involve participants from all the households of women and children participating in the RCT. Thus, the actual number of households involved this OAA study will depend on the number of households involved in the RCT. For example, if on average households have 1.5 participants then the actual number of households would be about 1600; the maximum number would be 2408 if only one participant were enrolled from each household and without attrition. The focus group discussion (FGD) will involve participants randomly selected from among those who participated in the quantitative survey.

ii. Subject recruitment

An adult household member will be recruited into the OAA study during the 3-week observation period between recruitment (informed consent) and the baseline biospecimen collection visit for the WRA, lactating woman, and/or child from the same household participating in the randomized trial. We will use the household's roster (which was completed for the RCT participant) to identify the adult household members (≥ 15 years of age) who are not recruited in the RCT. If a household has only one adult member not

recruited in the RCT, that household member will automatically become eligible to participate in the quantitative survey, and if selected, the FGD. If a household has ≥ 2 adult members not recruited in the RCT, only of them will be randomly selected (using a Kish Table) to participate in the quantitative survey, and if selected, the FGD. The selection of the households to participate in the FGDs will be built into the overall randomization scheme, so that equal or approximately equal number of households from the intervention and control arms will be selected for the FGDs.

Recruitment into the OAA study (quantitative survey, and if selected, the FGD) will be conducted by trained interviewers who are fluent in English and the local language (mainly Dagbani). Because many people do not read or write the local language well, the participant information sheet (and subsequently, the study questionnaires) will not be written into Dagbani, but interviewers will receive instructions for on-site translation of the questionnaires during training.

Subject recruitment into the OAA study will proceed as follows:
Trained interviewers will obtain informed consent after describing the study procedures in detail to potential participants, answering any questions the potential participant may have, and inviting the potential participant to take part in the study. Potential participants will indicate their informed consent to participate in the study by signing or thumbprinting the informed consent form. Subsequently, interviewers will assign a participant ID to the individual OAA study participant, which will be linked to the ID of the woman or child enrolled in the RCT from the same household. If a household has ≥ 2 adult members not recruited in the RCT and the individual randomly selected from that household declines to provide informed consent, then we will randomly select another potentially eligible individual from the same household and repeat the aforementioned enrolment procedures. The individual selected for the OAA study will remain eligible as long as at least one household member (WRA, lactating woman, or child) is recruited (given informed consent but not yet randomized) or enrolled (randomized) into the RCT; that is, if all RCT participants are excluded or withdraw consent, then the OAA participant would also be excluded.

iii. OAA data collection

a) Quantitative survey

For adult households members of non-pregnant non-lactating women of reproductive age (15 - 49 years old) and children 2-5 years of age, data on background characteristics and opinions and attitudes about any bouillon cubes use in household cooking will be collected at the time of recruitment to be followed by two rounds of quantitative survey during the 9-month intervention duration. One round of quantitative survey will take place within the first two months of receiving the study-supplied bouillon cubes in the household and the second round, within the last two months of receiving the study-supplied bouillon so that the two surveys will be last least 6 months apart. In each survey round, data on participants' opinions, attitudes, and acceptance towards the study-supplied bouillon cubes will be collected using multi-item Likert scales, which will include a set of closed-ended questions on a Likert scale (1: disagree completely, 2: disagree, 3: neither disagree nor agree, 4: agree, and 5: agree completely) for each of the three evaluation domains (opinions, attitudes, and acceptance). The 2 survey rounds will allow us to determine whether there is any change in the adult household members' OAA about the use of the

study-supplied bouillon cubes during the intervention. If the only participant in the household enrolled in the main trial is a non-pregnant lactating women, there will be only one round of quantitative OAA survey for the adult household member, which will be conducted at least one month into the 3 months of the intervention.

b) Focus Group Discussions

We will use the FGDs to investigate the opinions, attitudes, and acceptance related to the study bouillon cube use among the participants. We will conduct one round of FGD. In the case of OAA study participants in households with non-pregnant non-lactating women of reproductive age and children 2-5 years of age, the FGDs will be conducted during the 6-8 month period in between the 2 quantitative survey rounds. In the case of households with only a lactating woman participating in the trial, the FGDs will be conducted during the same period as the quantitative survey. As much as possible, focus groups will be stratified according to important respondent characteristics, for example, separate groups for men and women, or for younger and older women. All FGDs will be audio-recorded for complete transcripts, a note-taker will be present to record non-verbal expressions/postures (for the focus groups), and immediately following the focus group, the study staff will have a team debriefing to discuss and record overall impressions. Focus groups will be conducted using a pre-tested topic guide. Although the focus groups will occur in the local languages, the transcripts of the interviews and focus groups will be translated and recorded in English by two individuals to ensure accuracy. Any discrepancies between the two translations will be discussed and resolved between the two transcribers and a neutral third party.

8. Data and safety monitoring

Data collection will be monitored daily by field supervisors. The data manager (based at UC Davis) will produce a weekly report for monitoring of key variables, including study enrollment, attrition, missing data/forms, adherence, and selected quality control monitoring (e.g., descriptive analyses to identify potential outliers). This information will be communicated to the investigators as well as field supervisors, to identify actions required for the field team (e.g., changes in enrollment, refresher training, participant visits to encourage adherence, etc.).

A Data and Safety Monitoring Board (DSMB) will be established for this project. The board will consist of 3-5 members not otherwise involved in this study, without competing interests, and who are experts in child health and nutrition research. At least one member will be based in or have extensive experience working in Ghana and at least one member will be a physician and/or have expertise in infectious disease. In addition, at least one member will have expertise in biostatistics. Specific terms of reference for the DSMB will be reviewed and signed by DSMB members prior to beginning the trial. DSMB members will review the study protocol and confirm that they have no major reservations prior to agreeing to serve on the DSMB. The DSMB will meet at predefined intervals (with intermediate meetings added if deemed necessary by the committee or investigators) to review study progress (including recruitment, loss to follow up, and protocol compliance) and safety monitoring data. The DSMB may recommend actions or modifications, which will be addressed by the study investigators, who will notify and/or seek approval from the relevant ethical review committees, as appropriate. Blinded interim analysis of serious adverse events (SAE) reported for participants will be conducted for safety considerations. No interim analyses related to intervention efficacy are pre-planned.

9. Data analysis

Statistical analysis plans (SAPs) will be developed prior to conducting any data analysis. For the primary outcomes, investigators will remain blinded to treatment group assignment until the primary analysis code is finalized. For each objective, a flow diagram for participant recruitment, enrolment, allocation, and attrition will be prepared according to CONSORT guidelines. Descriptive analyses will help guide data cleaning.

Separate analyses will be conducted for each target group. Because randomization will occur at the household level and only one individual per target group will be enrolled per household (for a maximum of one WRA, one child, and one lactating woman), the participants will be considered independent with repeated measurements on the same participant being accounted for with robust standard errors (White, 1980). Additionally, a variable representing the community of residence of each participant will be included as a control covariate.

The primary analysis will be based on complete-case intention to treat and analysed as a pre-post study design, controlling for baseline measure of the outcome. Testing will be two-sided superiority testing. For continuous outcomes, this would be an ANCOVA approach with non-normal outcomes transformed prior to analysis (typically log transformed). For binary outcomes, we will use either logistic regression to estimate odds ratios or binomial log-link / modified poisson regression to estimate risk ratios (approach will be pre-specified in each SAP). In addition to the outcome-specific testing, certain analyses may use a global test to examine the simultaneous impact of the intervention on multiple outcomes.

We anticipate that randomization will lead to good balance in household and individual characteristics within the study sample. Therefore, we will rely on the minimally adjusted analysis (only controlling for baseline measure and community) as our primary analysis. For selected biomarkers (for example, **plasma** ferritin), an additional minimally adjusted analysis will include markers of inflammation (CRP and AGP) at each time point, if biologically relevant and if the outcome is associated with the markers of inflammation in bivariate analyses (details will be pre-specified in each SAP). In a secondary analysis, we will estimate adjusted parameters by including variables that are strongly associated with the outcome to potentially improve the precision of our estimates (decrease the SEs). Variable selection approach will be pre-specified in each SAP. Additionally, pre-specified effect modification testing will be conducted by including an interaction term in minimally adjusted models.

Substantial attrition (>20% loss) after randomization will trigger sensitivity analyses to assess the impact of missingness on inference. This will be conducted either by multiple imputation or inverse probability of censoring-weighted analysis (pre-specified in each SAP). We will also conduct a per protocol analysis using data on adherence to identify the subgroup of participants in “high compliance” households.

10. Training

All field staff (enumerators, phlebotomists, supervisors, etc.) and researchers will be trained on human subjects research ethics and how to ensure confidentiality of study participants. As part of the training, we will introduce field staff to the purpose and objectives of the study, and they will review the study manual and data collection forms thoroughly. Training will include demonstrations and hands-on practice sessions, with review of practice data used to determine

whether additional practice sessions are warranted. Refresher trainings on specific topics (e.g., anthropometry, dietary assessment, etc.) will be held at scheduled intervals, with additional sessions scheduled as needed based on data monitoring review or observations of field supervisors.

Phlebotomists will be trained laboratory personnel, each with at least a Bachelor's Degree in Medical Laboratory Science or Medical Laboratory Technology.

11. Quality assurance

Quality control of data collection will occur at several levels. All procedures will be described in a manual of Standard Operating Procedures (SOPs), which will form the basis for all training and supervision of the data collection teams. Supervisors will oversee all data collectors in the field on a daily basis, and their performance will be reviewed. The data manager will produce a weekly report for monitoring of key variables that are collected onto tablets and uploaded for storage each day. The data collection team supervisors will be alerted to any apparent anomalies in the data to enable appropriate investigation and corrective action. Study investigators will review data monitoring reports and hold regular meetings with the project manager at the study site to ensure timely resolution of issues that arise during data collection.
Standard materials (such as calibration weights) will be used to confirm the accuracy of anthropometric equipment. Quality control procedures for kits for point-of-care analyses (such as hemoglobin) or laboratory measurements will be conducted with calibrators and controls provided by manufacturers.

12. Expected outcomes of the study

This study will generate information on the effects of micronutrient-fortified bouillon cube on the micronutrient status of vulnerable individuals (women and young children). This information will be useful to national, regional and global stakeholders involved in assessing the potential for bouillon cubes to contribute to addressing deficiencies in one or more of the micronutrients examined.

13. Dissemination of results and publication policy

The results from this study will be disseminated in national and international conferences and published as Open Access publications in peer-reviewed journals. Dissemination workshops will be held with national stakeholders in Ghana as well as regional and district stakeholders in the study area. We will work with the Ghana Health Service in the Northern Region as well as the study districts to determine how to best disseminate the results in the region and the study districts. Study results will also be disseminated through the network of bouillon-related partners and stakeholders associated with this project, with particular focus on the West Africa region.

14. Duration of the project

The field work portion of this study has a duration of approximately 18 months (date of the first visit of the first participant to the date of the last visit of the last participant) (assuming recruitment takes places over ~6 months). The study is anticipated to take place between **late-**2022 and **early 2024** (see Work Plan below).

The end of trial will be defined as the date the last data is collected, inclusive of the analysis of biological samples of study participants (i.e., immediately following the completion of the laboratory analysis of outcomes listed in Tables 1 and 2 has been completed).

15. Problems anticipated

Withdrawals from the study might occur if a woman consents to participate without consulting other household members. We will therefore encourage the participating women (or caregivers of enrolled children) to consult with their husbands or partners, or any other influential household members (such as a mother-in-law) before consenting to participate. We will also seek permission from local authorities (e.g., local health administration and local chiefs) before starting the study.

As for any study that requires behaviour change, low adherence (use of the study bouillon) may pose a challenge which threatens the validity of the study. However, we believe this risk will be minimized by using products that have successfully undergone previous acceptability studies and by conducting the study in a population where bouillon is commonly consumed.

It is possible that participants or field staff working on the proposed project may have increased risk of exposure to certain hazards, such as vehicular accidents (e.g., during travel between Accra and the study site, or to participants' homes), or COVID-19 exposure. We will work to minimize the possibility of hazards to all participants and study workers by ensuring sufficient staff training before the study begins, and regular refresher training thereafter. We will follow standard protocols for minimizing COVID-19 exposure to participants and staff, and follow guidance from the Ghana Health Service. We will train field staff to be conscious of the possible hazards they face, and how to protect themselves at all times.

16. Project Management

The Principal Investigators (PIs) are Dr Seth Adu-Afarwuah (University of Ghana) and Dr Reina Engle-Stone (University of California, Davis, USA). Dr Adu-Afarwuah will oversee the execution of the project in Ghana, including being responsible for administrative and fiscal oversight. Dr Adu-Afarwuah will work collaboratively with Dr Engle-Stone and other co-investigators on the administration of data collection, training, analyses and write-up of results.

17. Consenting process:

Informed consent will be sought from all participants before any study procedures are initiated. Oral consent will be obtained to pose the initial eligibility questions to the respondent during participant recruitment. Written informed consent will then be obtained from the index participant (or a parent or guardian, in the case of children 2-5 years of age) for the remaining recruitment procedures as well as participation in the trial. **Written consent will also be obtained from the household member selected for the OAA study. Verbal consent will be obtained from the respondent for administration of the Willingness-To-Pay questionnaire at the recruitment visit.**

The consent form will be read to, or asked to be read by, potential participants. Those who respond "yes" will be asked to sign or thumbprint the informed consent. With regard to potential participants who cannot read, an interpreter will read the purpose and contents of the

Participants' Information Sheet to them and they will be asked to make a thumbprint in the presence of a neutral witness as their written consent.

The procedure for obtaining assent or consent from adolescent girls is based on the consent process that is used for the Ghana Demographic Health Survey, as follows:

"If the girl of reproductive age is 15-17 years and has ever been married, lived together with someone, divorced/separated or widowed, parental consent is not required. Obtain written consent from the 15-17 year old participant."

In this case, the girl would provide informed consent.

"If the woman of reproductive age is 15-17 years and lives with her parents (i.e. never been married and never lived together with someone), the Participants' Information Sheet should be read and consent procedures conducted in the presence of at least one parent."

In this case, at least one parent would provide informed consent for the girl to participate and the girl would provide informed assent.

Because the intervention involves a food that would be consumed by the entire household, participants will be encouraged to consult with other household members to inform their decision about whether to participate in the trial. In addition, the study team will request and document agreement from the head of household that they support the participation of one or more members of their household in the study, including activities such as the use of study-provided micronutrient-fortified bouillon in household cooking and biweekly home visits.

18. Data and/or Specimen Management and Confidentiality

All field staff and researchers will be trained to ensure confidentiality of study participants. Data will only be accessible for authorized members of the research team in Ghana and at UC Davis. Study procedures are designed so that all Personally Identifying Information (PII), such as name or address, is kept separately from participant data throughout data collection, storage, and analysis.

All identifying information (or PII) will be collected on paper forms. This information will be entered into a database and saved as passphrase-protected, encrypted files on a designated encrypted, password-protected laptop. No study files containing personal health data (participant data) will be stored on, or accessed from, this laptop. Designated field staff members will be identified to manage the data entry; they will not be involved in managing the de-identified participant data. A small amount of other data will be collected on paper forms, including quality monitoring forms (e.g., daily measurement of calibration weights on the scales). Consent forms will also be documented on paper. Completed paper forms will be stored in a secure location only accessible to trained personnel (e.g., a locked file cabinet). Informed consent forms will be scanned and stored in encrypted, password-protected files on our computer and external hard drives; consent forms and other participant information stored on the laptop will also be archived on password-protected folders on Box (separate from any folders containing de-identified participant data). The external hard drives will be stored in a locked office (and a safe). Consent forms will be retained for a minimum of 3 years for adults (or until the individual is 25 years of age, i.e. a minimum of 10 years for women who are 15 at enrollment) and 23 years for children (i.e., until the child reaches 25 years of age). After the end of the study and an embargo period,

we will de-identify all data and make the data available for public use, in accordance with the commitment to Global Access as described in section 19).

All other participant data will be collected directly on tablets using the SurveyCTO program, which has been thoroughly vetted through UC Davis IT Security protocols for this purpose. These data will be identified using a unique ID number for each participant and a corresponding bar code. Participants will receive a small paper card with their study ID and bar code at the time that they consent to participate in the study. A bar code scanner linked to the tablet will be used to confirm the correct participant ID at each visit. Tablets will be stored in a secure, locked area when not in use for data collection, and all tablets will be encrypted and password-protected to prevent release of data if tablets are stolen. Data will be uploaded from tablets to the secure SurveyCTO server each day. If internet access is inconsistent, data will be uploaded on the first day that internet connection is available. After data are uploaded, the data will be deleted from the tablet. For archiving and analysis, the de-identified data will be downloaded from SurveyCTO and stored separately from any identifying information in encrypted, password-protected files on our computer and external hard drives as well as password protected folders on Box.

In addition, photographs and brief video clips of study participants will be taken as they participate in study procedures. Photography/videography will adhere to the Ghana Health Service ERC guidelines. A specific box on the consent form will be used to mark participant consent to photos and videos. Photographs and videos will be used for purposes such as academic presentations, teaching, public education, or other not-for-profit activities. These files will be stored separately from study data.

The Ghana Health Services Ethical Review Committee, the Ghana Food and Drugs Authority, and the University of California, Davis Institutional Review Board may access the participants' data to verify the procedures and/or data of the trial without violating the confidentiality of the subject so far as the applicable laws and regulations permit.

Data Transfer Agreements will be developed between study collaborators, sponsor, and any organizations that may request the data; terms of the agreement will be subject to the data handling and storage procedures approved by the UC Davis IRB and GHS-ERC.

19. Data and/or Specimen Banking

Participant data will be identified with unique identification (ID) numbers and names will not be attached to the data, in order to ensure confidentiality. Cleaned databases will be stored in password-protected folders with access limited to the core research team. Additional details are provided above in the section on "Data and/or Specimen Management and Confidentiality".

The study sponsors require a commitment to Global Access for the products of their grant investments. To meet these requirements, we intend to 1) publish journal articles describing the study results with an Open Access license, 2) make available information about study methods, such as protocols or standard operating procedures, on the study website, and 3) make available de-identified datasets upon request by the study sponsor or other investigators. Material/Data Transfer Agreements will be developed between study collaborators, sponsor, and any organizations that may request the data or specimens; terms of the agreement will be subject to the data handling and storage

procedures approved by the UC Davis IRB and GHS-ERC. Researchers at external institutions who will analyze specimens will sign material transfer agreements and agree that (1) specimens are to be analyzed only for the purposes directed by the principal investigator and (2) specimens will either be returned or destroyed upon completion of these analyses.

Long-term specimen storage will occur at the University of Ghana (Dept. of Nutrition) in the laboratory of Dr. Seth Adu-Afarwuah and at UC Davis (Dept. of Nutrition) in the laboratory of Dr. Reina Engle-Stone.

20. Provisions to Monitor the Data to Ensure the Safety of Subjects

As noted above, Standard Operating Procedures based on Ghana Health Service guidelines will be followed to minimize risk of COVID-19 exposure for participants and field staff. At recruitment, individuals presenting with illness (e.g., COVID-19 diagnosis or exposure, fever [temperature ≥ 38°C], chills/shaking, dry cough, shortness of breath or difficulty breathing, loss of smell or taste within the past 72 hours) or danger signs (altered consciousness, seizures, refusal to eat or drink or vomiting of everything consumed) will not be enrolled and will be referred to the local health clinic for medical evaluation. After enrollment, COVID-19 diagnosis, COVID-19 exposure, and related symptoms will be assessed at each participant contact (biospecimen collection site visit or home visit). Individuals who have tested positive for COVID-19 or have known exposure to someone with COVID-19 will have study visits deferred or altered (e.g., "no-contact" bouillon drop-off") to avoid exposure to other participants or field staff.

Although the sample size was not estimated on the basis of detecting differences in morbidity symptoms by treatment group, we will assess reported morbidity symptoms (such as diarrhoea, fever, etc.) and hospital or health clinic visits on a biweekly basis among all participants (with information confirmed by review of health cards where possible), to better interpret micronutrient status indicators and as part of the safety monitoring plan. Body temperature will also be assessed at each visit and participants with fever [temperature ≥ 38°C] will be referred for medical evaluation. In addition, enrolled participants will be asked to report selected morbidity symptoms and adverse events, such as hospitalizations, that occur among any other household members. We will develop a detailed referral protocol for field staff to respond to cases of illness potentially identified during the morbidity recalls.

All suspected adverse events (AEs) will be documented according to standard operating procedures and reported to the GHS-ERC and UC Davis IRB in routine reporting. Suspected serious adverse events (SAE), such as deaths or hospitalization of study participants, will be reported immediately to the principal investigators, who will notify the GHS-ERC, the Ghana FDA, the UC Davis IRB and the DSMB in a timely manner as required by their policies; these agencies and committees will then determine whether modifications are required to ensure participant safety and welfare.

As described above, a Data and Safety Monitoring Board (DSMB) will be established for this project. The board will consist of 3-5 members not otherwise involved in this study, without competing interests, and who are experts in child health and nutrition research. At least one member will be a medical doctor. The DSMB will review study progress as well as safety considerations and will advise investigators on possible actions or modifications. Recommendations by the DSMB may include no action (trial continues as

planned), trial pause, early stopping (clear evidence of harm), extension of follow-up or protocol changes. Blinded interim analysis of SAE and selected AE (such as diarrhea and malaria) will be conducted for safety considerations and reviewed by the DSMB. No interim analyses related to intervention efficacy are pre-planned.

If the pattern of SAE suggests potential for harm in the intervention group, the DSMB may recommend that the study be stopped early. An early stopping recommendation (to discontinue recruitment and/or the provision of the intervention products) will be made only if the result is likely to convince a broad range of clinicians/nutritionists, including those supporting the trial and the general clinical community of clear evidence for harm in those receiving the multiple-micronutrient fortified bouillon cubes. No formal statistical rules will be set to determine the outcome of the DSMB review which should instead be based on the number of expected and unexpected SAEs in each group and their significance as judged by DSMB members. We have proposed the DSMB only decide to stop the trial based on an unexpectedly high frequency of SAEs in the group receiving multiple-micronutrient fortified bouillon compared to the control group (p-value for group difference in occurrence of SAEs has a value less than 0.01 in interim safety analyses).

In the case that the DSMB recommends early stopping of the study (either due to concerns about the bouillon product, the specific formulation of the bouillon in the active arm of the study, or both), this information will be communicated to the GHS-ERC within 3 days verbally and within 7 days in writing, to the Ghana FDA within 10 business days, and to the UC Davis IRB within 5 business days. Enrolment and the delivery of study bouillon to enrolled participants will be immediately paused, and any study bouillon in participant households will be collected, pending further decisions from the GHS-ERC and UC Davis IRB.

21. Withdrawal of Participants

Participants have the right to stop participation in the study at any time without giving a reason and their decision will not be held against them. The trial team may also withdraw a participant from the study if deemed necessary at any time taking into consideration the reasons mentioned below. The reason for a participant's premature termination will be documented according to the following reasons:

- Serious Adverse Event
- Adverse Event
- Participant's consent withdrawal
- Development of withdrawal criterion (e.g. severe illness, pregnancy, cessation of lactation)
- Protocol deviation
- Migrated/moved from the study area
- Lost to follow-up

All reasonable efforts will be made to maintain contact with participants and encourage their continued participation in the study. Loss-to-follow up will be determined at the end of the study and will refer to cases in which the study team is unable to contact a participant by the end of their scheduled participation and the lack of contact cannot be explained by any of the other categories above (e.g., consent withdrawal or migration from the study area).

In case the participant decides to withdraw participation or consent during the study, or is withdrawn from the study by the study team, any information already collected on the participant until the time of withdrawal will be used. Statistical analysis will include examination of the characteristics of participants who are withdrawn, who withdraw consent, or who are lost to follow up, in comparison with participants who complete the study. The study team will inquire about the reason for any withdrawal and follow-up with the participant regarding any unresolved AEs. For withdrawn participants no specific data will be collected. Participants will not be replaced as our sample size calculation takes into account attrition and loss to follow up.

22. Risks to Participants

The following procedures are in place to minimize risks to study participants:

We will exclude any participants who have experienced (or have household members who have experienced) adverse reactions to bouillon or common bouillon ingredients or who report that they or a household member receive frequent blood transfusions (e.g., due to severe thalassemia).

Given the COVID-19 pandemic, participant exposure to the virus from other participants or study workers who unknowingly have the virus may be possible. To minimize participants' exposure to infected persons or chances of infection, we have developed our project's Standard Operating Procedures (SOPs) for the prevention of COVID-19 for our field staff and study participants based on the Government of Ghana's as well as the GHS-ERC's recommendations.

We will refer participants for medical advice and treatment if they are found to have severe malnutrition (children), severe anaemia (children or WRA), or malaria infection (at baseline and endline). Participants who are randomized to complete the full study will have the costs of the national health insurance annual premium covered. Morbidity will be monitored at biweekly home visits and participants will be referred for follow up treatment based on symptoms reported during these visits.

The levels of micronutrients added to the bouillon are within the range of normal daily intakes for micronutrients and similar to micronutrient interventions that have been tested in other settings. Additional information on safety considerations from the micronutrient doses are reviewed above (Section "Fortified bouillon development") and in the technical appendix on fortification levels. Participants who become pregnant will be excluded from the study**.** Exclusion of study participants due to pregnancy is not based on any safety concerns (micronutrient levels in cubes are calculated to remain within the Tolerable Upper Index Level for different physiological groups, including pregnant women; see technical appendix)**; however, we will follow all index participants who become pregnant until the resolution of their pregnancy (miscarriage, still birth, live birth, etc.) via telephone interview to monitor pregnancy outcomes**. Participants who become pregnant will be excluded from the study for scientific reasons. This is because pregnancy affects metabolism of micronutrients and thus micronutrient biomarkers will not be comparable between women who are pregnant and non-pregnant study participants; this additional variation due to pregnancy would increase the "noise" in the primary outcomes, reducing statistical power. Although very early pregnancy may not be self-reported (for example, between baseline and screening, or between the 35wk and 38wk endline visits), fluctuations in micronutrient status due to pregnancy are likely to be limited at this very early stage. Participants are tested for pregnancy at the time of vitamin A-isotope

dosing (WRA only) and are excluded from the study if pregnant because the RID method for estimating total body vitamin A stores has not been validated among pregnant women. We will not provide advice on contraceptives. If women have questions, we will suggest they speak with a medical professional at their primary health care facility.

Some interview questions related to sensitive topics such as household possessions or health information may cause discomfort for the participant. We will minimize this risk by conducting interviews in a private area. A "private area" might mean a separate room, a curtained space, or a space out of hearing range of other study participants. If a private area cannot be secured during a household interview (e.g., the recruitment questionnaire prior to enrolment) due to household and cultural norms, sensitive health related questions will be skipped and asked in a private area during another contact with the participant (mobile biospecimen collection site).

All procedures will be explained to the participants during the informed consent process, and again immediately before they are conducted. Participants will be encouraged to ask questions at any time during the study. Participant privacy and confidentiality practices will be addressed during training of all study personnel. Additional provisions to protect the privacy interest of participants are detailed above in Sections "Data management and confidentiality" and "Data and/or Specimen Banking". Participants will also be reminded of the procedures in place to ensure participant confidentiality. Social or psychological distress could potentially result if information on socio-economic status or health of participants were made public. However, the risk of the release of such information in this study is minimal due to the following precautions: All participant information (except the consent form) will be associated with study ID number and not with a name, address or other identifying information; data collected on tablets will be password-protected; and all other study materials will be kept in a locked room that is accessible only to trained study personnel.

This study does not involve any direct cost to the participant. The study may involve indirect cost to the participant because of the time required to participate. For visits to the biospecimen collection site for biospecimen collection, we will attempt to limit these visits to 4 hours per day. Enumerator visits to the participants' homes will be limited to 2 hours per visit for longer visits involving recruitment or dietary data collection and to 30 minutes for biweekly bouillon ration dropoffs and routine questions (adherence, morbidity). If, any point in time, participants have to use public transportation to come to the central research location, we will ask them how much they paid for the transportation, and we will reimburse the entire amount paid. For households that require transportation but do not have access to public transportation, the study team will arrange to bring them to the biospecimen collection site. To recognize the time spent on study activities, each participant will receive a bar of Key Soap at specified time points: 1) recruitment, 2) screening (WRA only), 3) completion of the baseline visit, 4) completion of the first endline visit, and 5) completion of the second endline visit. In addition, participants who complete the full study will receive an additional bar of soap and a medium-size stainless steel cooking pan. Participants will also receive a supply of bouillon cubes for their household for the entire study duration (9 months for non-pregnant, non-lactating women and children 2-5 years, and 3 months for lactating women).

23. Potential benefits to participants

Participants may or may not benefit from consuming the multiple micronutrient-fortified bouillon cube. Participants will receive information on their anthropometric status, anaemia status, malaria status, and blood pressure (women only) at the time of enrolment and at the endline visit. The study will cover the cost of the national health insurance annual premium for the duration of the study for each participant who is randomized after the baseline visit. Participants will receive enough bouillon for their entire household for the duration of the study. In addition, members of the study team will be trained to recognize danger signs requiring referral of participants to local medical services.

24. Sharing of results with participants

Participants will receive their results for selected measures at the baseline and endline data collection points, including 1) haemoglobin concentration, 2) blood pressure (women only), 3) anthropometry (weight and height), and 4) malaria. These results will be provided on a piece of paper for the participant on the day of measurement, along with information on the normal ranges for these tests. Participants with abnormal values will be referred for medical advice.

25. Prior approvals and funding information

Apart from ethics approval being sought from Ghana Health Service Ethical Review Committee (GHS- ERC), the University of California, Davis, USA (UC Davis) IRB Human Subjects Committee will approve the protocol prior to commencing the research project. The Regional (Northern Region) and District (Tolon and Kumbungu Districts) Directors of Health will be informed about this project before data collection begins. The **clinical trial** will be approved by the Ghana FDA prior to initiation of the study.

A material and data transfer agreement will be established between UC Davis and the University of Ghana prior to completion of the field activities.

The study is supported by a grant to the University of California, Davis, from Helen Keller International (through funding from a grant from the Bill & Melinda Gates Foundation). None of the study investigators has financial or other conflicts of interest to disclose in relation to this research.

26. Compensation for research-related injury

All individuals presenting with illness will be referred to the usual local health services. Any adverse events arising during the study will be treated within the national health system. For participants who are randomized to a study group after the baseline visit, the study will cover the cost of enrollment of study participants in the national health insurance. The project will have insurance which covers necessary medical treatment of serious adverse events only for cases where the serious adverse event is directly caused by the study products or procedures. The project will not provide any other form of compensation for injury.

27. Economic burden to participants

The only cost to participants is time spent on study activities. If participants require transportation to the mobile biospecimen collection site, the study team will either arrange their transport or pay for their transport. We will provide material gifts (Key Soap) rather than monetary incentive to compensate participants for their time.

To recognize the time spent on study activities, each participant will receive a bar of Key Soap at specified time points: 1) recruitment, 2) screening (WRA only), 3) completion of the baseline visit, 4) completion of the first endline visit, and 5) completion of the second endline visit. Participants will also receive a supply of bouillon cubes for their household for the entire study duration (9 months for non-pregnant, non-lactating women and children 2-5 years, and 3 months for lactating women). Finally, participants who complete the study will receive an additional bar of soap and a medium-size stainless steel cooking pan.

28. Work Plan

	2022						2023												2024						
	J	A	S	O	N	D	J	F	M	A	M	J	J	A	S	O	N	D	J	F	M	A	M	J	J
Administrative and ethical approvals (ERC, FDA, IRB)	■	■	■																						
Confirm study bouillon cube formulation	■																								
Bouillon production		■	■																						
Preparation of Standard Operating Procedures	■	■	■																						
Order supplies	■	■	■																						
Revise and pre-test questionnaires		■	■																						
Field team training			■																						
Recruitment				■	■	■	■	■	■																
Baseline data collection					■	■	■	■	■	■															
Bouillon delivery					■	■	■	■	■	■	■	■	■	■	■	■	■	■	■	■					
Endline data collection														■	■	■	■	■	■	■					
Data cleaning and monitoring				■	■	■	■	■	■	■	■	■	■	■	■	■	■	■	■	■	■	■	■		
Biospecimen shipping																					■				
Laboratory analysis																						■	■	■	■
Data analysis																					■	■	■	■	■
Preliminary results																									■

29. Study Budget.

BUDGET SUMMARY (USD)

	Period 1	Period 2	Period 3	Total
	11/1/21-<br>6/30/22	7/1/22-<br>6/30/23	7/1/23 -<br>6/30/24	2/1/20 -<br>6/30/24
Personnel	98,722	192,800	13,000	370,914
Equipment	14,400	0	0	88,363
Travel	21,937	32,489	0	65,709
Materials and Supplies	18,480	8,300	0	31,005
Other Direct Costs	58,780	82,026	19,600	205,092
Total Direct Costs	193,839	307,315	32,600	730,079
Indirect Costs (15%)	29,076	46,097	4,890	120,854
Total Costs (Direct + Indirect)	222,915	353,412	37,490	850,933

The total budget of this randomized trial is estimated at USD 850,933.

30. References

Abizari, A. R., Dold, S., Kupka, R., & Zimmermann, M. B. (2017). More than two-thirds of dietary iodine in children in northern Ghana is obtained from bouillon cubes containing iodized salt. *Public Health Nutr, 20*(6), 1107-1113. doi:10.1017/S1368980016003098

Abizari, A. R., Pilime, N., Armar-Klemesu, M., & Brouwer, I. D. (2013). Cowpeas in Northern Ghana and the factors that predict caregivers' intention to give them to schoolchildren. *PLoS One, 8*(8), e72087. doi:10.1371/journal.pone.0072087

Allen, L. H., Miller, J. W., de Groot, L., Rosenberg, I. H., Smith, A. D., Refsum, H., & Raiten, D. J. (2018). Biomarkers of Nutrition for Development (BOND): Vitamin B-12 Review. *J Nutr, 148*(suppl_4), 1995S-2027S. doi:10.1093/jn/nxy201

Bailey, L. B., Stover, P. J., McNulty, H., Fenech, M. F., Gregory, J. F., 3rd, Mills, J. L., . . . Raiten, D. J. (2015). Biomarkers of Nutrition for Development-Folate Review. *J Nutr, 145*(7), 1636S-1680S. doi:10.3945/jn.114.206599

Bailey, R. L., West, K. P., Jr., & Black, R. E. (2015). The epidemiology of global micronutrient deficiencies. *Ann Nutr Metab, 66 Suppl 2*, 22-33. doi:10.1159/000371618

Ballot, D. E., MacPhail, A. P., Bothwell, T. H., Gillooly, M., & Mayet, F. G. (1989). Fortification of curry powder with NaFe(111)EDTA in an iron-deficient population: report of a controlled iron-fortification trial. *Am J Clin Nutr, 49*(1), 162-169. doi:10.1093/ajcn/49.1.162

Brown, K. H., Rivera, J. A., Bhutta, Z., Gibson, R. S., King, J. C., Lönnerdal, B., . . . Hotz, C. (2004). International Zinc Nutrition Consultative Group (IZiNCG) Technical Document #1. Assessment of the risk of zinc deficiency in populations and options for its control. *Food Nutr Bull, 25*(1 Suppl 2), S99-203. doi:https://doi.org/10.4067/S0717-75182010000200014

Chen, J., Zhao, X., Zhang, X., Yin, S., Piao, J., Huo, J., . . . Chen, C. (2005). Studies on the effectiveness of NaFeEDTA-fortified soy sauce in controlling iron deficiency: a population-based intervention trial. *Food Nutr Bull, 26*(2), 177-186; discussion 187-179. doi:10.1177/156482650502600201

Cook, J. D., Flowers, C. H., & Skikne, B. S. (2003). The quantitative assessment of body iron. *Blood, 101*(9), 3359-3364.

Engle-Stone, R., Nankap, M., Ndjebayi, A. O., & Brown, K. H. (2014). Simulations based on representative 24-h recall data predict region-specific differences in adequacy of vitamin A intake among Cameroonian women and young children following large-scale fortification of vegetable oil and other potential food vehicles. *J Nutr, 144*(11), 1826-1834. doi:10.3945/jn.114.195354

Erhardt, J. G., Estes, J. E., Pfeiffer, C. M., Biesalski, H. K., & Craft, N. E. (2004). Combined measurement of ferritin, soluble transferrin receptor, retinol binding protein, and C-reactive protein by an inexpensive, sensitive, and simple sandwich enzyme-linked immunosorbent assay technique. *J Nutr, 134*(11), 3127-3132. doi:https://doi.org/10.1093/jn/134.11.3127

Garcia-Casal, M. N., Pena-Rosas, J. P., McLean, M., De-Regil, L. M., Zamora, G., & consultation working, g. (2016). Fortification of condiments with micronutrients in public health: from proof of concept to scaling up. *Ann N Y Acad Sci, 1379*(1), 38-47. doi:10.1111/nyas.13185

Gladstone, M., Lancaster, G., Umar, E., Nyirenda, M., Kayira, E., van den Broek, N., & Smyth, R. L. (2010). The Malawi Developmental Assessment Tool (MDAT): The creation, validation, and reliability of a tool to assess child development in rural African settings. *PloS Med, 7*, e1000273.

GoG. (2013). National Nutrition Policy For Ghana 2013–2017 [Internet]. Government of Ghana, Accra, Ghana [cited 2019 Nov 18]. Available from: https://extranet.who.int/nutrition/gina/sites/default/files/GHA%202013%20National%20Nutrition%20Policy.pdf. In.

Hess, S. Y., Brown, K. H., Sablah, M., Engle-Stone, R., Aaron, G. J., & Baker, S. K. (2013). Results of Fortification Rapid Assessment Tool (FRAT) surveys in sub-Saharan Africa and suggestions for future modifications of the survey instrument. *Food Nutr Bull, 34*(1), 21-38. doi:10.1177/156482651303400104

Huo, J., Sun, J., Miao, H., Yu, B., Yang, T., Liu, Z., . . . Li, Y. (2002). Therapeutic effects of NaFeEDTA-fortified soy sauce in anaemic children in China. *Asia Pac J Clin Nutr, 11*(2), 123-127. doi:10.1046/j.1440-6047.2002.00277.x

Klassen-Wigger, P., Geraets, M., Messier, M. C., Detzel, P., Lenoble, H. P., & Barclay, D. V. (2018). Micronutrient fortification of bouillon cubes in Central and West Africa. In: Food fortification in a globalized world, pages 363-372. DOI: org/10.1016/B978-0-12-802861-2.00039-0. In.

Kumordzie, S. K., Adams, K. P., Arnold, C. D., Tan, X., Vosti, S. A., & Engle-Stone, R. (2019). Assessment of the potential Impact of bouillon fortification on micronutrient Intakes in Ghana using a household expenditure survey. Submission to Micronutrient Forum 2020 conference:.

Mannar, V., & Gallego, E. B. (2002). Iron fortification: country level experiences and lessons learned. *J Nutr, 132*(4 Suppl), 856S-858S. doi:10.1093/jn/132.4.856S

Spohrer, R., Knowles, J., Jallier, V., Ndiaye, B., Indorf, C., Guinot, P., & Kupka, R. (2015). Estimation of population iodine intake from iodized salt consumed through bouillon seasoning in Senegal. *Ann N Y Acad Sci, 1357*, 43-52. doi:10.1111/nyas.12963

Theary, C., Panagides, D., Laillou, A., Vonthanak, S., Kanarath, C., Chhorvann, C., . . . Moench-Pfanner, R. (2013). Fish sauce, soy sauce, and vegetable oil fortification in Cambodia: where do we stand to date? *Food Nutr Bull, 34*(2 Suppl), S62-71. doi:10.1177/15648265130342S108

Univ Ghana/GroundWork/Univ Wisconsin-Madison/KEMRI/UNICEF. (2017). Ghana Micronutrient Survey [Internet]. UNICEF-Accra, Accra, Ghana [cited 2019-05-21].Available from: http://groundworkhealth.org/wp-content/uploads/2018/06/UoG-GroundWork_2017-GHANA-MICRONUTRIENT-SURVEY_Final_180607.pdf.

White, H. 1980. A heteroskedasticity-consistent covariance matrix estimator and a direct test for heteroskedasticity. Econometrica 48: 817–830.

World Health Organization. Iron deficiency anaemia: assessment, prevention, and control. A guide for programme managers. Geneva, World Health Organization, 2001.

INFORMATION SHEET FOR NON-PREGNANT NON-LACTATING WOMEN OF REPRODUCTIVE AGE (Version 3: 29 August 2022)

This information sheet is for non-pregnant and non-lactating women between the ages of 15-49 years who we are inviting to participate in a randomized controlled trial

Title of Study: Effect of household use of multiple micronutrient-fortified bouillon on micronutrient status among women and children in two districts in the Northern Region of Ghana.

Introduction: My name is ________________, and I would like to tell you about a study the University of Ghana is going to do in this area, together with the University of California, Davis in the USA. The study is led by Dr. Seth Adu-Afarwuah and Dr. Reina Engle-Stone and their contact information is:

**Address**	Department of Nutrition and Food Science, University of Ghana
	PO Box LG 134, Legon, Accra
**Telephone #**	030 396 4078
**Email**	sadu-afarwuah@ug.edu.gh

Background and purpose of research: In Ghana, anaemia (i.e. not having enough blood) and not having enough of the things called vitamins and minerals (such as vitamin A and iron), which our bodies need to make blood and stay healthy, are common among women of reproductive age and children. Not having enough of vitamins and minerals does not give any feelings as we get when we are hungry, but can make children not grow well or do well in school, or make children and women not have enough energy to do the work in the house or on the farm. Not having enough of vitamins and minerals also increases the chances of children getting sick or even dying. We know that people in Ghana and other countries in West Africa use bouillon in cooking, so adding vitamins and minerals to bouillon cubes might be a good way to give women and children more of the vitamins and minerals they need. We are doing the research in this area to find out if adding vitamins and minerals to bouillon cubes will work the way we think it will work.

Nature of research: We are inviting you to take part in this study, in which we will test different types of bouillon cubes: one has only iodine added (like most of the cubes on the market), while the other has several vitamins and minerals (such as iodine, iron and vitamin A) added. We will give women in the study bouillon cubes to use in their household's cooking for about 10 months. Near the beginning of the study, and again near the end of the study, we will measure how much blood the women have and also how much of certain vitamins and minerals the women have in their blood. This will let us see if adding vitamins and minerals to bouillon cube works well to help people get more of the vitamins and minerals they need. In all, about 1028 women between the ages of 15 and 49 who are not pregnant or breastfeeding, 690 women who are breastfeeding, and 690 children 2-5 years old will take part in this study. The health authorities at Ghana Health Service in Accra have allowed us to do this study, and we have also obtained permission from the health authorities in Tamale and Tolon/Kumbungu, as well as from the Chief.

Participants Involvement:

Duration/what is involved: This research will take about 10 months. If you decide to take part, here are the things we will do:

1. Whilst we are here today, we will ask you questions about yourself and your family, your health, the foods you and your family usually eat, and how you use bouillon cubes in cooking. Then we will make an appointment for you to come to our sample collection site – it is like a lab.
2. When you come, we will ask about your general health and check your urine to see if you are pregnant. We will also take a few drops of blood by finger-prick to see if you have anaemia, malaria, or some sign of illness. If you are pregnant or severely anaemic, you cannot continue in the study. If you have malaria or some sign of illness, we will ask you to come back in a few weeks for us to check you again. If you can continue in the study, we will need to measure how much vitamin A is in your body. To do this, we will give you a capsule containing "heavy vitamin A" to take with a glass of water, and then we will give you crackers with chocolate spread to eat, to help your body absorb the "heavy vitamin A". "Heavy vitamin A" is like the vitamin A that is found in food but it is slightly heavier, that is why we call it "heavy vitamin A". This visit will take about 2 hours of your time.
3. After your visit to the sample collection site, we will visit your household two more times, about two weeks apart. At each visit we will ask a few more questions about your health, the foods you and your family eat, and how you use bouillon cubes in cooking.
4. Next, we will make a second appointment for you to come to our sample collection site or lab. When you come, we will ask questions about your general health as we did before. If you are sick that day, we will ask you to come back in a few weeks for us to check you again. If you are not sick that day, we will ask you some questions about your diet and measure your height, weight and blood pressure. We will take a small amount of your blood (up to 12 ml or 2.5 teaspoons) from your arm to see if you have anaemia, malaria or some sign of illness and we will also measure the amounts of certain vitamin and minerals (such as vitamin A and iron) in your body. Finally, we will collect a small amount of your urine to help us know whether the women in this area eat enough iodine. This visit will take about 2 hours of your time.
5. Next, we will ask you to pick one out of 4 envelopes, and the one you pick will tell the type of bouillon you will receive. Some women will receive the bouillon cubes which have only iodine added. Other women will receive the bouillon cubes which have several vitamins and minerals added. All the bouillon cubes will be shrimp-flavoured. Until we have completed the study, neither you nor the people doing the study will know which bouillon cube you will get.
6. When we know (from the enveloped you picked) the type of bouillon you will receive, our study staff will visit you in your home the next day to deliver the bouillon cubes. If you have any bouillon in your home at that time, which you purchased, we will pay you the cost of that bouillon and collect it from you so that it does not mix up with the bouillon we gave you. At that visit we will also ask you a few questions about the foods you usually eat.
7. Study staff will visit you in your home once every two weeks to find out how you are doing. Each time we visit, we will ask you questions about your general health, your diet, and how many of the study bouillon cubes you used since your last visit. The study staff will also ask you if you know, or a nurse/midwife/doctor has told you, that you are pregnant.

8. If you know, or a nurse/midwife/doctor has told you, that you are pregnant, then we will ask you to finish the main part of the study. In the case, we will take your phone number and make sure we can call you from time to time (every two months) to see how you are you faring until you have given birth.
9. If you know that you are not pregnant, or no nurse/midwife/doctor has told you that you are pregnant, then will give you a new supply of bouillon cubes, as needed. At every other visit (every 4 weeks), we will ask questions about you and your household, including the foods you usually eat, how you and your household use the bouillon cubes in your cooking, and what you think about the bouillon cubes. On average, each of the visits will take up to about 30 minutes of your time.
10. After about eight months, we will make the third and the fourth (i.e., two more) appointments for you to come to our sample collection site. These third and fourth visits will be about 3 weeks apart, and each will take about 2 hours of your time. If you are sick at either visit, we may ask you to come back in a few weeks for us to check you again.
11. At the third visit, we will test your urine to see if you are pregnant, and we will keep a small amount of your urine to measure if women in this area eat enough iodine.
    i. If you are not pregnant, we will measure your height, weight, and blood pressure, and ask some questions about your diet and general health. We will then take a small amount of your blood (up to 8 ml or 1.5 teaspoons) from your arm to test for anaemia, malaria, or some sign of illness and to measure the amounts of certain vitamins and minerals (such as vitamin A and iron) in your body.
    ii. If you are not pregnant and you do not have malaria or some sign of illness and you are not severely anaemic, we will ask you a few more questions about your diet. Then, we will give you a capsule containing "heavy vitamin A" as we did before to help us measure how much vitamin A is in your body. Then we will ask you to come back for the fourth visit.
    iii. If you are not pregnant and the tests show you have malaria or some sign of illness or you do not have enough blood, we will not give you the "heavy vitamin A" that day, but we will still ask you to come back for the fourth visit.
    iv. If we find that you are pregnant, we will not take your blood, or measure your weight and blood pressure, or give you the "heavy vitamin A" because those things are for when women are not pregnant. In that case, you will finish the main part of the study, but we will take your phone number and make sure we can call you from time to time (every two months) to see how you are you faring until you have given birth.
12. At the fourth visit, we will measure your blood pressure and ask some questions about your diet and general health.
    i. We will let you know if we need to test your urine to see if you are pregnant and if we need to take a small amount of your blood (up to 12 ml or 2.5 teaspoons) from your arm to test for anaemia, malaria, or some sign of illness and also to measure the amounts of certain vitamins and minerals (such as vitamin A and iron) in your body. We will also let you know if you have finished the study.
    ii. If we find that you are pregnant, we will not take your blood, or measure your weight and blood pressure, or give you the "heavy vitamin A" because those things are for when women are not pregnant. In that case, you will finish the main part of the study, but we will take your phone number and make sure we can call you from time to time (every two months) to see how you are you faring until you have given birth.

iii. If you did not receive the "heavy vitamin A" at the third visit because of malaria or some sign of illness, and you are not pregnant, then we will let you know if you can take the "heavy vitamin A" at this visit. If you take the "heavy vitamin A," then we will ask you to return to the sample collection site 3 weeks later. At that visit, we will take a small amount of your blood (up to 6 ml or about 1.25 teaspoons) from your arm to measure the amount of vitamin A in your body, and then you will finish the study.
iv. Finally, we may visit your home one last time to take any leftover bouillon cube from the study.

13. During this study, we may take photographs or videos of you, your family and your environment. The photos and videos we take will be used only for the purpose of teaching, research and public service. If we show this picture or video to anyone, we will not tell them you or your family's names or any of your information. At the end of this form, we will ask you whether you agree or do not agree with allowing us to use the photographs and/or videos for these purposes. Your choice will not be held against you. If you agree to let us take a photo or video, you will not come to us now or later to request payment or credit for the photograph or video.

Potential Risks: From what we know, there is not anything really bad that can happen to women who take part in this study. The blood draw can cause some pain, make you feel uncomfortable, and result in bruising, but these will be only for a short time, and it is very rare that they will result in an infection or other complaints. You may also feel uncomfortable answering some of the questions we ask you, but you are free to not answer a question if you wish. You may feel tired whilst answering the interview questions, but you can take a break at any time.

To protect participants in this COVID-19 era, we will follow all Ghana Health Service advice such as the use of masks, social distancing, temperature checks, and COVID-19 exposure screenings, etc., to make sure you do not increase the chance of getting COVID-19 because of this study.

Benefits: You may not benefit directly by participating in this study. However, if we find that you have high blood pressure, malaria, or severe anaemia, we will refer you to the nearest health centre. Moreover, you may benefit from the nutrients in the bouillon cubes. The results of this study may help us find another way to give vitamins and minerals to women in this and other places, so that they can be healthy.

Costs: If you take part in this study, it will not cost you any money. If you need to take a transport to come to the data collection site or lab, we will ask you how much you paid for the transport and reimburse you for the entire amount paid. If you do not have access to transportation, we will arrange transportation for you.

Compensation: To thank you for taking part in this study, we will give you 1 long bar of Key Soap today, and another long bar of Key Soap each time you come to the sample collection site, as well as an additional bar of soap when you complete the study, for a total of 6 bars. If you complete the study, we will also give you one medium-sized stainless steel cooking pan. Each time you come to the sample collection site or lab, we will also give you a light breakfast. After you pick an envelope telling us what study group you are in, we will enrol you in the National Health Insurance Scheme if you are not covered. If you are already covered and your coverage expires while you are still in the study, we will

pay the annual premium to renew it for you. The project has an insurance to cover the treatment of serious adverse events which are judged to be directly caused by the study products or procedures.

Confidentiality: The records of this study will be kept secure at the University of Ghana, and the University of California, Davis and your name together with information about you and your family will not be given to anybody outside of the study team. We will protect information about you to the best of our ability. Your name and your information will not be associated with any photographs or videos of yourself. You will not be named in any reports that will come out of the study. The Ethics Committees of the Ghana Health Service and the University of California, Davis, and the Ghana Food and Drugs Authority, however, will have the authority to review your research records to ensure proper oversight of the study.

Voluntary participation/withdrawal: Participation in this research is entirely voluntary. You may decide not to take part in the research. If you choose to take part, you can change your mind at any time and withdraw from the study. If you decide not to participate or decide to withdraw at any time, it will not be held against you and you do not have to tell us your reasons.

Outcome and Feedback: Findings from the study will be shared with the district health administrations of the Tolon and Kumbungu districts. We will work with the district health administrations to determine when and how to best disseminate the study findings to the community.

Appropriate alternative procedures and treatment: You are free to decide if you want to be in this research or not. You may also change your mind about your participation and quit after the study has started.

Feedback to participant: Anything we measure on you will be for research only, and will not be reviewed by a **doctor**. However, if we find that you have high blood pressure, malaria or severe anaemia, we will let you know, so that you can seek medical attention.

Funding information: This study is being paid for by Helen Keller International, through the help it received from the Bill & Melinda Gates Foundation, in the USA.

Sharing of participants information/data: The information collected in this study will be owned by the University of Ghana and the University of California, Davis. Whenever we are asked to make the information from the study available to anyone outside of the study team or make it publicly available, your name and any other identifying information will be removed so that no one will know anything about you and your family. Once your name and other identifying information have been removed, so that there is no link between you and the data, we may share the data with other investigators or organizations for additional future research. We will seek permission from the Ghana Health Services Ethics Review Committee before doing any such future research.

Storage of samples: In case there are any leftover samples, we would like to keep them for possible future research purposes. Your name and any other identifying information will not be on the specimen. The research that may be done with the samples may not benefit you directly and you will not receive any profit from it. We will seek permission from the Ghana Health Service Ethics Review Committee before doing any such research.

Provision of information and consent for participants: After you have signed or thumb-printed the Consent Form, a copy of the Information sheet and Consent form will be given to you to keep.

Who to Contact for Further Clarification/Questions: If you have any questions about the study, you are welcome to call the University of Ghana Principal-Investigator, Prof. Seth Adu-Afarwuah on Tel. # 030 396 4078, and he will be happy to answer your questions. For issues related to your participation in this study, you may call Nana Abena Kwaa Ansah Apatu, the ERC Administrator, Ghana Health Service Ethics Review Committee on 0503539896, or email ethics.research@ghs.gov.gh

University of Ghana, Legon; University of California, Davis

CONSENT FORM

Title of Study: Effect of household use of multiple micronutrient-fortified bouillon on micronutrient status among women and children in two districts in the Northern Region of Ghana.

PARTICIPANTS' STATEMENT

If the woman of reproductive age is 15-17 years and has ever been married, lived together with someone, divorced/separated or widowed, parental consent is not required. Obtain consent from the 15-17 year old participant.

I acknowledge that I have read or have had the purpose and contents of the Participants' Information Sheet read and satisfactorily explained to me in a language I understand (_______________ *language*) I fully understand the contents and any potential implications as well as my right to change my mind (i.e. withdraw from the research) even after I have signed this form.

I voluntarily agree to be part of this research.

Name of Participant....................................

Participants' Signature ....OR Thumb Print.................................

Date:.......................................

PARENTAL CONSENT FOR WOMEN 15-17 YEARS

If the woman of reproductive age is 15-17 years and lives with her parents (i.e. never been married and never lived together with someone), the Participants' Information Sheet should be read and consent procedures conducted in the presence of at least one parent.

I acknowledge that I have read or have had the purpose and contents of the Participants' Information Sheet read and satisfactorily explained to me in a language I understand (_______________ *language*) I fully understand the contents and any potential implications as well as my right to change my mind (i.e. withdraw my child/spouse from the research) even after I have signed this form.

I voluntarily agree for my child to be part of this research.

Name of Child ....

Name of Child's Parent...

Signature .... OR Thumb Print...............................

Date:.....................................

CHILD ASSENT

I acknowledge that I have read or have had the purpose and contents of the Participants' Information Sheet read and satisfactorily explained to me in a language I understand (_______________ *language*) I fully understand the contents and any potential implications as well as my right to change my mind (i.e. withdraw from the research) even after I have assented.

Do you voluntarily agree to be part of this research? YES ☐ NO ☐

Name of Child ....

Signature .... OR Thumb Print...............................

We may take photographs and videos of you, your family and your environment. However, before we can take photographs and videos, we need your consent. Your personal information will not be associated with any photographs and videos. As mentioned above, the photographs and videos will be used for teaching students, or when presenting research findings. If you agree or not agree that we can take photographs and videos, kindly indicate below:

	May we take photographs and videos? ***(Kindly tick if you want to be photographed and videoed or not)***
Yes	
No	

In case there is any leftover blood or urine sample, we would like to keep it for possible future research purposes. If you agree or not that your leftover sample may be kept for future possible research purposes, please indicate below:

May we keep your leftover urine or blood sample for future research? YES ☐ NO ☐

INTERPRETERS' STATEMENT

I interpreted the purpose and contents of the Participants' Information Sheet to the afore named participant to the best of my ability in the ________________ language to his proper understanding.

All questions, appropriate clarifications sort by the participant and answers were also duly interpreted to his/her satisfaction.

Name of Interpreter.. Signature of Interpreter..

Date:........................ Contact Details...

STATEMENT OF WITNESS

I was present when the purpose and contents of the Participant Information Sheet was read and explained satisfactorily to the participant in the language he/she understood (______________ *language*).

I confirm that he/she was given the opportunity to ask questions/seek clarifications and same were duly answered to his/her satisfaction before voluntarily agreeing to be part of the research.

Name:.. Signature...

OR Thumb Print .... Date:...........................

INVESTIGATOR STATEMENT AND SIGNATURE

I certify that the participant has been given ample time to read and learn about the study. All questions and clarifications raised by the participant have been addressed.

Researcher's name...................................... Signature ....

Date..

University of Ghana, Legon; University of California, Davis

INFORMATION SHEET FOR MOTHERS/CARETAKERS OF YOUNG CHILDREN
(Version 3: 29 August 2022)

This information sheet is for the mothers/caretakers of children between the ages of 2-5 years who we are inviting to participate in a randomized controlled trial

Title of Study: Effect of household use of multiple micronutrient-fortified bouillon on micronutrient status among women and children in two districts in the Northern Region of Ghana.

Introduction: My name is _______________, and I would like to tell you about a study the University of Ghana is going to do in this area, together with the University of California, Davis in the USA. The study is led by Dr. Seth Adu-Afarwuah and Dr. Reina Engle-Stone and their contact information is:

**Address**	Department of Nutrition and Food Science, University of Ghana<br>PO Box LG 134, Legon, Accra
**Telephone #**	030 396 4078
**Email**	sadu-afarwuah@ug.edu.gh

Background and purpose of research: In Ghana, anaemia (i.e. not having enough blood) and not having enough of the things called vitamins and minerals (such as vitamin A and iron), which our bodies need to make blood and stay healthy, are common among women of reproductive age and children. Not having enough of vitamins and minerals does not give any feelings as we get when we are hungry, but can make children not grow well or do well in school, or make children and women not have enough energy to do the work in the house or on the farm. Not having enough of vitamins and minerals also increases the chances of children getting sick or even dying. We know that people in Ghana and other countries in West Africa use bouillon in cooking, so adding vitamins and minerals to bouillon cubes might be a good way to give women and children more of the vitamins and minerals they need. We are doing the research in this area to find out if adding vitamins and minerals to bouillon cubes will work the way we think it will work.

Nature of research: We are inviting you to allow your child to take part in this study, in which we will test different types of bouillon cubes: one has only iodine added (like most of the cubes on the market), while the other has several vitamins and minerals (such as iodine, iron and vitamin A) added. We will give the families of children in the study bouillon cubes to use in their household's cooking for about 10 months. Near the beginning of the study, and again near the end of the study, we will measure how much blood the children have and also how much of certain vitamins and minerals the children have in their blood. This will let us see if adding vitamins and minerals to bouillon cube works well to help people get more of the vitamins and minerals they need. In all, about 690 children 2-5 years old, 1028 women between the ages of 15 and 49 who are not pregnant or breastfeeding, and 690 women who are breastfeeding will take part in this study. The health authorities at Ghana Health Service in Accra have allowed us to do this study, and we have also obtained permission from the health authorities in Tamale and Tolon/Kumbungu, as well as from the Chief.

Participants Involvement:

Duration/what is involved: This research will take about 10 months. If you decide that your child will be a part of this study, here are the things we will do:

1. Whilst we are here today, we will ask you questions about your child and your family, your child's health, the foods your child and your family usually eat, and how you use bouillon cubes in cooking.
2. Next, we will visit your child 2 more times, about 2 weeks apart. At each visit we will
   a. Ask a few more questions about your child's general health, the foods the family eats, and how bouillon cubes are used in the family cooking.
   b. Ask questions about the child's home situation, how the mother/caregiver spends time together with the child, and what toys the child plays with.
   c. Let the child play some games with small toys and ask him/her to name certain pictures, so that we can measure how well the child can speak, move things around, and think about certain things.
   d. Ask the child to play a computer game made for children to measure how well the child can do things children his/her age usually do (development).

   These tests can take place in your home, or we will invite you to come to a central location. We expect each visit will take approximately one hour
3. Next, we will make an appointment for you to bring your child to our sample collection site – it is like a lab. When your child comes, we will ask questions about your child's general health. If your child is sick that day, we will ask you to bring your child back in a few weeks for us to check him/her again. If your child is not sick that day, we will ask you some questions about your child's diet, and measure your child's height, weight and blood pressure. We will take a small amount of your child's blood (up to 7.5 ml or 1.5 teaspoons) from his/her arm to see if s/he has anaemia, malaria or some sign of illness and we will also measure the amounts of certain vitamin and minerals (such as vitamin A and iron) in your child's body. If your child is severely anaemic or severely malnourished, s/he cannot be in the study. If your child is selected to play the same computer game again at this visit, we will let you know. Finally, we will collect a small amount of your child's stool to see if s/he has some sign of illness, and a small amount of your child's urine to help us know whether the children in this area eat enough iodine. This visit will take about 2 hours of your time.
4. Next, we will ask you to pick one out of 4 envelopes, and the one you pick will tell the type of bouillon your child and your household will receive. Some children will receive the bouillon cubes which have only iodine added. Other children will receive the bouillon cubes which have several vitamins and minerals added. All the bouillon cubes will be shrimp-flavoured. Until we have completed the study, neither you, nor the people doing the study, will know which bouillon cube you will get.
5. When we know (from the enveloped you picked) the type of bouillon your child will receive, we will visit you in your home the next day to deliver the bouillon cube. If you have any bouillon in your home at that time, which you purchased, we will pay you the cost of that bouillon and collect it from you so that it does not mix up with the bouillon we gave you. At this visit we will also ask you a few questions about the foods you usually eat.

6. Study staff will visit you and your child in your home once every two weeks to find out how your child is doing. Each time we visit, we will ask you questions about your child's general health, your child's diet, and how many of the study bouillon cubes you used since your last visit. We will also give you a new supply of bouillon cubes, as needed. At every other visit (every 4 weeks), we will ask questions about your child and your household, including how you and your household use the bouillon cubes in your cooking and what you think about the bouillon cubes. On average, each of the visits will take up to about 30 minutes of your time.
7. After about eight months, we will make the second and the third (i.e., two more) appointments for your child to come to our sample collection site. These second and third visits will be about 3 weeks apart, and each will take about 2 hours of your time. If your child is sick at either visit, we may ask you to bring your child back in a few weeks for us to check him/her again.
8. Between the second and third visits, we will ask you some questions about the child's home situation and how you and your child spent time together, and measure how well the child can speak, move things around, and think about certain things as we did before.
9. At the second visit, we will measure your child's height, weight and blood pressure and ask some questions about his/her diet and general health. We will then take a small amount of your child's blood (up to 7.5 ml or 1.5 teaspoons) from your child's arm to test for anaemia, malaria, or some sign of illness and to measure the amounts of certain vitamins and minerals (such as vitamin A and iron) in your child's body. We will also collect a small amount of your child's stool to see if s/he has some sign of illness, and a small amount of your child's urine to help us know whether the children in this area eat enough iodine. If your child is severely anaemic or severely malnourished, your child can continue to be in the study, but we will not take your child's blood again. At the third visit, we will measure your child's blood pressure, ask some questions about his/her diet and general health, and let you know if the child has finished the study. If your child was not severely anaemic at the second visit, we will take a small amount of your child's blood (up to 7.5 ml or 1.5 teaspoons) from your child's arm to test for anaemia, malaria, or some sign of illness and to measure the amounts of certain vitamins and minerals (such as vitamin A and iron) in your child's body, before letting you know that the child has finished the study. We may visit your home one last time to take any leftover bouillon cube from the study.
10. During this study, we may take photographs and/or videos of your child, your family and your environment. The photos and/or videos we take will be used only for the purpose of teaching, research and public service. If we show this picture or video to anyone, we will not tell them you or your family's names or any of your information. At the end of this form, we will ask you whether you agree or do not agree with allowing us to use the photographs and/or videos for these purposes. Your choice will not be held against you. If you agree to let us take a photo or video, you will not come to us now or later to request payment or credit for the photograph or video.

Potential Risks: From what we know, there is not anything really bad that can happen to children who take part in this study. The blood draw can cause some pain, make your child feel uncomfortable, and result in bruising, but these will be only for a short time, and it is very rarely that they will result in an infection or other complaints. You may feel uncomfortable answering some of the questions we ask you, but you are free to not answer a question if you wish. You may feel tired whilst answering the interview questions, but you can take a break at any time.

To protect participants in this COVID-19 era, we will follow all Ghana Health Service advice such as the use of masks, social distancing, temperature checks, and COVID-19 exposure screenings, etc. to make sure you do not increase the chance of getting COVID-19 very much just because of this study.

Benefits: Your child may not benefit directly by participating in this study. However, if we find that your child has severe acute malnutrition, malaria, or severe anaemia, we will refer your child to the nearest health centre. Moreover, your child may benefit from the nutrients in the bouillon cubes. The results of this study may help us find another way to give vitamins and minerals to children in this and other places, so that they can be healthy.

Costs: If your child takes part in this study, it will not cost you or your child any money. If you need to take a transport to come to the data collection site or lab, we will ask you how much you paid for the transport and reimburse you the entire amount paid. If you do not have access to transportation, we will arrange transportation for you.

Compensation: To thank your child for taking part in this study, we will give him/her 1 long bar of Key Soap today, and another long bar of Key Soap each time s/he come to the sample collection site, as well as an additional bar of soap when s/he completes the study; for a total of 5 bars. If your child completes the study, we will also give him/her a medium-sized stainless steel cooking pan. Each time you and your child come to the sample collection site or lab, we will also give you and your child a light breakfast. After you pick an envelope telling us what study group your child is in, we will enrol your child in the National Health Insurance Scheme if your child is not covered. If your child is already covered and his/her coverage expires while s/he is still in the study, we will pay the annual premium to renew it for your child. The project has an insurance to cover the treatment of serious adverse events which are judged to be directly caused by the study products or procedures.

Confidentiality: The records of this study will be kept secure at the University of Ghana, and the University of California, Davis and your child's name together with information about your child and your family will not be given to anybody outside of the study team. We will protect information about your child to the best of our ability. You and your child's name, and your information, will not be associated with any photographs or videos. Your child will not be named in any reports that will come out of the study. The Ethics Committees of the Ghana Health Service and the University of California, Davis, and the Ghana Food and Drugs Authority, however, will have the authority to review your child's research records to ensure proper oversight of the study.

Voluntary participation/withdrawal: Participation in this research is entirely voluntary. You may decide not to have your child take part in the research. If you choose to have your child take part, you can change your mind at any time and withdraw him/her from the study. If you decide not to participate or decide to withdraw your child at any time, it will not be held against you or your child and you do not have to tell us your reasons.

Outcome and Feedback: Findings from the study will be shared with the district health administrations of the Tolon and Kumbungu districts. We will work with the district health administrations to determine when and how to best disseminate the study findings to the community.

Appropriate alternative procedures and treatment: You are free to decide if you want your child to be in this research or not. You may also change your mind about your child's participation and quit after the study has started.

Feedback to participant: Anything we measure on your child will be for research only and will not be reviewed by a doctor. However, if we find that your child has severe acute malnutrition, malaria or severe anaemia, we will let you know, so that you can seek medical attention for your child.

Funding information: This study is being paid for by Helen Keller International, through the help it received from the Bill & Melinda Gates Foundation, in the USA.

Sharing of participants information/data: The information collected in this study will be owned by the University of Ghana and the University of California, Davis. Whenever we are asked to make the information from the study available to anyone outside of the study team or make it publicly available, your child's name and any other identifying information will be removed so that no one will know anything about your child and your family. Once your child's name and other identifying information have been removed, so that there is no link between your child and the data, we may share the data with other investigators or organizations for additional future research. We will seek permission from the Ghana Health Services Ethics Review Committee before doing any such future research.

Storage of samples: In case there are any leftover samples, we would like to keep them for possible future research purposes. Your child's name and any other identifying information will not be on the specimen. The research that may be done with the samples may not benefit your child directly and your child will not receive any profit from it. We will seek permission from the Ghana Health Service Ethics Review Committee before doing any such research.

Provision of information and consent for participants: After you have signed or thumb-printed the Consent Form, a copy of the Information sheet and Consent form will be given to you to keep.

Who to Contact for Further Clarification/Questions: If you have any questions about the study, you are welcome to call the University of Ghana Principal-Investigator, Prof. Seth Adu-Afarwuah on Tel. # 030 396 4078, and he will be happy to answer your questions. For issues related to your participation in this study, you may call Nana Abena Kwaa Ansah Apatu, the ERC Administrator, Ghana Health Service Ethics Review Committee on 0503539896, or email ethics.research@ghs.gov.gh

University of Ghana, Legon; University of California, Davis

CONSENT FORM

Title of Study: Effect of household use of multiple micronutrient-fortified bouillon on micronutrient status among women and children in two districts in the Northern Region of Ghana.

PARTICIPANTS' STATEMENT

I acknowledge that I have read or have had the purpose and contents of the Participants' Information Sheet read and satisfactorily explained to me in a language I understand (_______________ *language*) I fully understand the contents and any potential implications as well as my right to change my mind (i.e. withdraw my child from the research) even after I have signed this form.

I voluntarily agree for my child to be part of this research.

Name of Child.................................

Name Child's Parent...

Signature ....OR Thumb Print................................

Date:......................................

We may take photographs and videos of you, your family and your environment. However, before we can take photographs and videos, we need your consent. Your personal information will not be associated with any photographs and videos. As mentioned above, the photographs and videos will be used for teaching students, or when presenting research findings. If you agree or not agree that we can take photographs and videos, kindly indicate below:

	May we take photographs and videos?<br>***(Kindly tick if you want to be photographed and videoed or not)***
Yes	
No	

In case there is any leftover blood or urine sample, we would like to keep it for possible future research purposes. If you agree or not that your child's leftover sample may be kept for future possible research purposes, please indicate below:

May we keep your child's leftover urine, stool or blood sample for future research? YES ☐

NO ☐

INTERPRETERS' STATEMENT

I interpreted the purpose and contents of the Participants' Information Sheet to the afore named participant to the best of my ability in the ___________________ language to his proper understanding.

All questions, appropriate clarifications sort by the participant and answers were also duly interpreted to his/her satisfaction.

Name of Interpreter.. Signature of Interpreter...

Date:.......................... Contact Details...

STATEMENT OF WITNESS

I was present when the purpose and contents of the Participant Information Sheet was read and explained satisfactorily to the participant in the language he/she understood (_______________ *language*).

I confirm that he/she was given the opportunity to ask questions/seek clarifications and same were duly answered to his/her satisfaction before voluntarily agreeing to be part of the research.

Name:... Signature...

OR Thumb Print .... Date:...............................

INVESTIGATOR STATEMENT AND SIGNATURE

I certify that the participant has been given ample time to read and learn about the study. All questions and clarifications raised by the participant have been addressed.

Researcher's name.. Signature ....

Date...

INFORMATION SHEET FOR LACTATING WOMEN
(Version 3: 29 August 2022)

This information sheet is for non-pregnant women who are lactating and between the ages of 15-49 years who we are inviting to participate in a randomized controlled trial

Title of Study: Effect of household use of multiple micronutrient-fortified bouillon on micronutrient status among women and children in two districts in the Northern Region of Ghana.

Introduction: My name is _________________, and I would like to tell you about a study the University of Ghana is going to do in this area, together with the University of California, Davis in the USA. The study is led by Dr. Seth Adu-Afarwuah and Dr. Reina Engle-Stone and their contact information is:

**Address**	Department of Nutrition and Food Science, University of Ghana
	PO Box LG 134, Legon, Accra
**Telephone #**	030 396 4078
**Email**	sadu-afarwuah@ug.edu.gh

Background and purpose of research: In Ghana, anaemia (i.e. not having enough blood) and not having enough of the things called vitamins and minerals (such as vitamin A and iron), which our bodies need to make blood and stay healthy, are common among women of reproductive age and children. Not having enough of vitamins and minerals does not give any feelings as we get when we are hungry, but can make children not grow well or do well in school, or make children and women not have enough energy to do the work in the house or on the farm. Not having enough of vitamins and minerals also increases the chances of children getting sick or even dying. We know that people in Ghana and other countries in West Africa use bouillon in cooking, so adding vitamins and minerals to bouillon cubes might be a good way to give women and children more of the vitamins and minerals they need. We are doing the research in this area to find out if adding vitamins and minerals to bouillon cubes will work the way we think it will work.

Nature of research: We are inviting you to take part in this study, in which we will test different types of bouillon cubes: one has only iodine added (like most of the cubes on the market), while the other has several vitamins and minerals (such as iodine, iron and vitamin A) added. We will give women in the study bouillon cubes to use in their household's cooking for about 4 months. Near the beginning of the study, and again near the end of the study, we will measure how much of certain vitamins and minerals the women have in their breastmilk, to see how much of these nutrients they feed to their children. This will let us see if adding vitamins and minerals to bouillon cube works well to help people get more of these vitamins and minerals they need. In all, about 690 women who are breastfeeding, 1028 women between the ages of 15 and 49 who are not pregnant or breastfeeding, and 690 children 2-5 years old will take part in this study. The health authorities at Ghana Health Service in Accra have allowed us to do this study, and we have also obtained permission from the health authorities in Tamale and Tolon/Kumbungu, as well as from the Chief.

Participants Involvement:

Duration/what is involved: This research will take about 4 months. If you decide to take part, here are the things we will do:

1. Whilst we are here today, we will ask you questions about yourself and your family, your health, the foods you and your family usually eat, and how you use bouillon cubes in cooking.
2. Next, we will visit you two more times, about two weeks apart. At each visit we will ask a few more questions about your general health, the foods you and your family eat, and how you use bouillon cubes in cooking.
3. Next, we will make an appointment for you to come to our sample collection site – it is like a lab. When you come, we will again ask questions about your general health. If you are sick that day, we will ask you to come back in a few weeks for us to check you again. If you are not sick that day, we will ask you some questions about your diet, and measure your height and weight. We will ask you to give breastmilk from one of your breasts using a breast pump, so that we will measure the amounts of certain vitamins (such as vitamin A) in your breastmilk. We will only need a small amount (up to 10 ml or 2 teaspoons), so if the amount of breastmilk pumped is more than the 2 teaspoons and you want the rest back, you can have it and feed it to your baby with a cup and a spoon. You will give the breastmilk in a private area. This visit will take about 2 hours of your time.
4. Next, we will ask you to pick one out of 4 envelopes, and the one you pick will tell the type of bouillon you will receive. Some women will receive the bouillon cubes which have only iodine added. Other women will receive the bouillon cubes which have several vitamins and minerals added. All bouillon cubes will be shrimp-flavoured. Neither you, nor the people doing the study, will know which bouillon cube you will get.
5. When we know (from the enveloped you picked) the type of bouillon you will receive, we will visit you in your home the next day to deliver the bouillon cube. If you have any bouillon in your home at that time, which you purchased, we will pay you the cost of that bouillon and collect it from you so that it does not mix up with the bouillon we gave you. At this visit we will also ask you a few questions about the foods you usually eat.
6. Study staff will visit you in your home once every two weeks to find out how you are doing. Each time we visit, we will ask you questions about your general health, your diet, and how many of the study bouillon cubes you used since your last visit. The study staff will also ask you if you know, or a nurse/midwife/doctor has told you, that you are pregnant.
7. If you know, or a nurse/midwife/doctor has told you, that you are pregnant, then we will ask you to finish the main part of the study. In the case, we will take your phone number and make sure we can call you from time to time (every two months) to see how you are you faring until you have given birth.
8. If you know that you are not pregnant, or no nurse/midwife/doctor has told you that you are pregnant, then we will give you a new supply of bouillon cubes, as needed. At every other visit (every 4 weeks), we will ask questions about you and your household, including the foods you usually eat, how you and your household use the bouillon cubes in your cooking, and what you think about the bouillon cubes. On average, each of the visits will take up to about 30 minutes of your time.

9. After about three months, we will make the second and third (i.e., two more) appointments for you to come to our sample collection site. The second and third visits will be about 1 week apart, and each will take about 2 hours of your time. If you are sick at either visit, we may ask you to come back in a few weeks for us to check you again.
10. At each of these visits, we will ask if you are pregnant. If you are pregnant, we will ask you to finish the main part of the study, and we will take your phone number and make sure we can call you from time to time (every two months) to see how you are you faring until you have given birth.
11. At each of these visits, if you are not pregnant, then we will collect a small amount of your breastmilk (up to 10 ml or 2 teaspoons) just like before, to measure the amounts of certain vitamins (such as vitamin A) in your breastmilk, and that means you have finished the study.
12. Finally, we may visit your home one last time to take any leftover bouillon cube from the study.
13. During this study, we may take photographs and/or videos of you, your family and your environment. The photos and/or videos we take will be used only for the purpose of teaching, research and public service. If we show this picture and/or video to anyone, we will not tell them you or your family's names or any of your information. At the end of this form, we will ask you whether you agree or do not agree with allowing us to use the photographs and/or videos for these purposes. Your choice will not be held against you. If you agree to let us take a photo or video, you will not come to us now or later to request payment or credit for the photograph or video.

Potential Risks: From what we know, there is not anything really bad that can happen to women who take part in this study. The amount of breastmilk we will collect is small and will not reduce how much breastmilk there is for your child. You may feel uncomfortable answering some of the questions we ask you, but you are free to not answer a question if you wish. You may feel tired whilst answering the interview questions, but you can take a break at any time.

To protect participants in this COVID-19 era, we will follow all Ghana Health Service advice such as the use of masks, social distancing, temperature checks, and COVID-19 exposure screenings, etc. to make sure you do not increase the chance of getting COVID-19 very much just because of this study.

Benefits: You may not benefit directly by participating in this study. You may benefit from the nutrients in the bouillon cubes. The results of this study may help us find another way to give vitamins and minerals to women in this and other places, so that they can be healthy.

Costs: If you take part in this study, it will not cost you any money. If you need to take a transport to come to the data collection site or lab, we will ask you how much you paid for the transport and reimburse you the entire amount paid. If you do not have access to transportation, we will arrange transportation for you.

Compensation: To thank you for taking part in this study, we will give you 1 long bar of Key Soap today, and another long bar of Key Soap each time you come to the sample collection site, as well as an additional bar of soap when you complete the study; for a total of 5 bars. If you complete the study, we will also give you a medium-sized stainless steel cooking pan. Each time you come to the sample collection site or lab, we will also give you a light breakfast. After you pick an envelope telling us what study group you are in, we will enrol you in the National Health Insurance Scheme if you are

not covered. If you are already covered and your coverage expires while you are still in the study, we will pay the annual premium to renew it for you. The project has an insurance to cover the treatment of serious adverse which are judged to be directly caused by the study products or procedures.

Confidentiality: The records of this study will be kept secure at the University of Ghana, and the University of California, Davis and your name together with information about you and your family will not be given to anybody outside of the study team. We will protect information about you to the best of our ability. Your name and your information will not be associated with any photographs or videos of yourself. You will not be named in any reports that will come out of the study. The Ethics Committees of the Ghana Health Service and the University of California, Davis, and the Ghana Food and Drugs Authority, however, will have the authority to review your research records to ensure proper oversight of the study.

Voluntary participation/withdrawal: Participation in this research is entirely voluntary. You may decide not to take part in the research. If you choose to take part, you can change your mind at any time and withdraw from the study. If you decide not to participate or decide to withdraw at any time, it will not be held against you and you do not have to tell us your reasons.

Outcome and Feedback: Findings from the study will be shared with the district health administrations of the Tolon and Kumbungu districts. We will work with the district health administrations to determine when and how to best disseminate the study findings to the community.

Appropriate alternative procedures and treatment: You are free to decide if you want to be in this research or not. You may also change your mind about your participation and quit after the study has started.

Feedback to participant: Anything we measure on you will be for research only, and will not be reviewed by a doctor.

Funding information: This study is being paid for by Helen Keller International, through the help it received from the Bill & Melinda Gates Foundation, in the USA.

Sharing of participants information/data: The information collected in this study will be owned by the University of Ghana and the University of California, Davis. Whenever we are asked to make the information from the study available to anyone outside of the study team or make it publicly available, your name and any other identifying information will be removed so that no one will know anything about you and your family. Once your name and other identifying information have been removed, so that there is no link between you and the data, we may share the data with other investigators or organizations for additional future research. We will seek permission from the Ghana Health Services Ethics Review Committee before doing any such future research.

Storage of samples: In case there are any leftover samples, we would like to keep them for possible future research purposes. Your name and any other identifying information will not be on the specimen. The research that may be done with the samples may not benefit you directly and you will not receive any profit from it. We will seek permission from the Ghana Health Service Ethics Review Committee before doing any such research.

Provision of information and consent for participants: After you have signed or thumb-printed the Consent Form, a copy of the Information sheet and Consent form will be given to you to keep.

Who to Contact for Further Clarification/Questions: If you have any questions about the study, you are welcome to call the University of Ghana Principal-Investigator, Prof. Seth Adu-Afarwuah on Tel. # 030 396 4078, and he will be happy to answer your questions. For issues related to your participation in this study, you may call Nana Abena Kwaa Ansah Apatu, the ERC Administrator, Ghana Health Service Ethics Review Committee on 0503539896, or email ethics.research@ghs.gov.gh

University of Ghana, Legon; University of California, Davis

CONSENT FORM

Title of Study: Effect of household use of multiple micronutrient-fortified bouillon on micronutrient status among women and children in two districts in the Northern Region of Ghana.

PARTICIPANTS' STATEMENT

If the woman of reproductive age is 15-17 years and has ever been married, lived together with someone, divorced/separated or widowed, parental consent is not required. Obtain consent from the 15-17 year old participant.

I acknowledge that I have read or have had the purpose and contents of the Participants' Information Sheet read and satisfactorily explained to me in a language I understand (_______________ *language*) I fully understand the contents and any potential implications as well as my right to change my mind (i.e. withdraw from the research) even after I have signed this form.

I voluntarily agree to be part of this research.

Name of Participant..................................

Participants' Signature ....OR Thumb Print................................

Date:.......................................

PARENTAL CONSENT FOR WOMEN 15-17 YEARS

If the woman of reproductive age is 15-17 years and lives with her parents (i.e. never been married and never lived together with someone), the Participants' Information Sheet should be read and consent procedures conducted in the presence of at least one parent.

I acknowledge that I have read or have had the purpose and contents of the Participants' Information Sheet read and satisfactorily explained to me in a language I understand (_______________ *language*) I fully understand the contents and any potential implications as well as my right to change my mind (i.e. withdraw my child/spouse from the research) even after I have signed this form.

I voluntarily agree for my child to be part of this research.

Name of Child ....

Name of Child's Parent..

Signature .... OR Thumb Print...............................

Date:.....................................

CHILD ASSENT

I acknowledge that I have read or have had the purpose and contents of the Participants' Information Sheet read and satisfactorily explained to me in a language I understand (______________ *language*) I fully understand the contents and any potential implications as well as my right to change my mind (i.e. withdraw from the research) even after I have assented.

Do you voluntarily agree to be part of this research? YES ☐ NO ☐

Name of Child ....

Signature .... OR Thumb Print...............................

We may take photographs and videos of you, your family and your environment. However, before we can take photographs and videos, we need your consent. Your personal information will not be associated with any photographs and videos. As mentioned above, the photographs and videos will be used for teaching students, or when presenting research findings. If you agree or not agree that we can take photographs and videos, kindly indicate below:

	May we take photographs and videos? ***(Kindly tick if you want to be photographed and videoed or not)***
Yes	
No	

In case there is any leftover breastmilk sample, we would like to keep it for possible future research purposes. If you agree or not that your leftover sample may be kept for future possible research purposes, please indicate below:

May we keep your leftover breastmilk sample for future research? YES ☐ NO ☐

INTERPRETERS' STATEMENT

I interpreted the purpose and contents of the Participants' Information Sheet to the afore named participant to the best of my ability in the ________________ language to his proper understanding.

All questions, appropriate clarifications sort by the participant and answers were also duly interpreted to his/her satisfaction.

Name of Interpreter.. Signature of Interpreter...

Date:.......................... Contact Details...

STATEMENT OF WITNESS

I was present when the purpose and contents of the Participant Information Sheet was read and explained satisfactorily to the participant in the language he/she understood (______________ *language*).

I confirm that he/she was given the opportunity to ask questions/seek clarifications and same were duly answered to his/her satisfaction before voluntarily agreeing to be part of the research.

Name:.. Signature...

OR Thumb Print .... Date:................................

INVESTIGATOR STATEMENT AND SIGNATURE

I certify that the participant has been given ample time to read and learn about the study. All questions and clarifications raised by the participant have been addressed.

Researcher's name... Signature ....

Date...

INFORMATION SHEET FOR ADULT HOUSEHOLD MEMBERS (Version 1: 29 August 2022)

This information sheet is for adult household members of women and children enrolled in the randomized trial who we are inviting to participate in a study to determine opinions, attitude, and acceptance towards the bouillon cubes used in the randomized trial

Title of Study: Randomized controlled trial of multiple micronutrient-fortified bouillon among women and children in two districts in the Northern Region of Ghana: opinions, attitudes, and acceptance towards the study bouillon among adult household members.

Introduction: My name is ________________, and I would like to tell you about another part of the study the University of Ghana and the University of California, Davis in the USA are doing in this area. The study is led by Dr. Seth Adu-Afarwuah and Dr. Reina Engle-Stone and their contact information is:

**Address**	Department of Nutrition and Food Science, University of Ghana
	PO Box LG 134, Legon, Accra
**Telephone #**	030 396 4078
**Email**	sadu-afarwuah@ug.edu.gh

Background and purpose of research: In Ghana, anaemia (i.e. not having enough blood) and not having enough of the things called vitamins and minerals (such as vitamin A and iron), which our bodies need to make blood and stay healthy, are common among women of reproductive age and children. Not having enough of vitamins and minerals does not give any feelings as we get when we are hungry, but can make children not grow well or do well in school, or make children and women not have enough energy to do the work in the house or on the farm. Not having enough of vitamins and minerals also increases the chances of children getting sick or even dying. We know that people in Ghana and other countries in West Africa use bouillon in cooking, so adding vitamins and minerals to bouillon cubes might be a good way to give women and children more of the vitamins and minerals they need. We are doing the research in this area to find out if adding vitamins and minerals to bouillon cubes will work the way we think it will work.

Nature of research: We are inviting you to take part in a short study, in which we will ask adult household members of women and children in the main study what they think and feel and do (accordingly) about the bouillon cubes we are testing. Women and children in the main study will receive an amount of bouillon cubes every two weeks, which the household members will use as they typically use bouillon. We are inviting you to take part in this short study because a woman and/or a child from your household is/are taking part in the main study. We expect adult household members from up to 2,408 households in the area to take part in this short study. Only one adult household member per household will be selected for this short study. The health authorities at Ghana Health Service in Accra have allowed us to do this study, and we have also obtained permission from the health authorities in Tamale and Tolon/Kumbungu, as well as from the Chief.

Participants Involvement:

Duration/what is involved: This research will take 9 months, but if there is only one individual from your household taking part in the main study and the individual is a lactating woman, then it will take 3 months. You will be in this short study as long as at least one household member is in the main study. If the household member or members in the main study withdraw(s) from the study, then you will not be part of the short study any longer.

If you decide to take part of the short study, here are the things we will do:

1. Whilst we are here today, we will ask you questions about yourself and your household, and what you think and do about bouillon cube use in household cooking.
2. If the woman and/or child from your household stays in the main study, then we will visit you again some time (1-2 months) after your household has received the study bouillon. At that visit, we will ask you what think and do about the bouillon cubes we have given to your household so far. Those questions will take about 25 minutes of your time.
3. We may select you to take part in a focus group discussion; we will let you know if you are selected.
4. If you are not selected for the focus group discussion and there is only one individual from your household taking part in the main study, and the individual is a lactating woman, it means you have finished taking part in this short study.
5. If you are selected for the focus group discussion, we will schedule a time with you at which you will join 6 to 9 other people in the community who have also agreed to take part in the study to discuss issues about the study bouillon cubes, including what you think and do about them. The focus group discussion will take about 1 hr of your time, and we will let you know later where in the community it will take place. We will audio-record the discussion, so that we will not miss some of the things people will say.
6. After the focus group discussion, you will finish taking part in the short study if there is only one individual from your household taking part in the main study, and the individual is a lactating woman.
7. If there is an individual from your household taking part in the main study, and the individual is not a lactating woman, then we will visit you again after 7-8 months. At that time, we will ask you some of the same questions we asked you before, that is, what you think and do about the bouillon cubes we have given to your household. This will be the end of the short study.
8. During this study, we may take photographs or videos of you, your family and your environment. The photos and videos we take will be used only for the purpose of teaching, research and public service. If we show this picture or video to anyone, we will not tell them you or your family's names or any of your information. At the end of this form, we will ask you whether you agree or do not agree with allowing us to use the photographs and/or videos for these purposes. Your choice will not be held against you. If you agree to let us take a photo or video, you will not come to us now or later to request payment or credit for the photograph or video.

Potential Risks: From what we know, there is not anything really bad that can happen to individuals who take part in this short study. You may feel uncomfortable answering some of

the questions we ask you, but you are free to not answer a question if you wish. You may feel tired whilst answering the interview questions, but you can take a break at any time.

To protect participants in this COVID-19 era, we will follow all Ghana Health Service advice such as the use of masks, social distancing.

Benefits: You may not benefit directly by participating in this study. However, the results of this study may help us find another way to give vitamins and minerals to women in this and other places, so that they can be healthy.

Costs: If you take part in this study, it will not cost you any money. If you need to take a transport to come to the focus group discussion, we will ask you how much you paid for the transport and then pay you back. If you do not have access to transportation to come to the focus group discussion, we will arrange transportation for you.

Compensation: To thank you for taking part in this study, we will give you 1 long bar of Key Soap today, another long bar of Key Soap if get selected and attend the focus group discussion, and yet another long bar of soap when we visit you home the second time.

Confidentiality: The records of this study will be kept secure at the University of Ghana, and the University of California, Davis and your name together with information about you and your family will not be given to anybody outside of the study team. We will protect information about you to the best of our ability. Your name and your information will not be associated with any photographs or videos of yourself. You will not be named in any reports that will come out of the study. The Ethics Committees of the Ghana Health Service and the University of California, Davis, and the Ghana Food and Drugs Authority, however, will have the authority to review your research records to ensure proper oversight of the study.

Voluntary participation/withdrawal: Participation in this research is entirely voluntary. You may decide not to take part in the research. If you choose to take part, you can change your mind at any time and withdraw from the study. If you decide not to participate or decide to withdraw at any time, it will not be held against you and you do not have to tell us your reasons.

Outcome and Feedback: Findings from the study will be shared with the district health administrations of the Tolon and Kumbungu districts. We will work with the district health administrations to determine when and how to best disseminate the study findings to the community.

Appropriate alternative procedures and treatment: You are free to decide if you want to be in this research or not. You may also change your mind about your participation and quit after the study has started.

Feedback to participant: Anything we measure on you will be for research only.

Funding information: This study is being paid for by Helen Keller International, through the help it received from the Bill & Melinda Gates Foundation, in the USA.

Sharing of participants information/data: The information collected in this study will be owned by the University of Ghana and the University of California, Davis. Whenever we are asked to

make the information from the study available to anyone outside of the study team or make it publicly available, your name and any other identifying information will be removed so that no one will know anything about you and your family. Once your name and other identifying information have been removed, so that there is no link between you and the data, we may share the data with other investigators or organizations for additional future research. We will seek permission from the Ghana Health Services Ethics Review Committee before doing any such future research.

Storage of samples: We will not collect any biological samples from you if you take part in this short study.

Provision of information and consent for participants: After you have signed or thumb-printed the Consent Form, a copy of the Information sheet and Consent form will be given to you to keep.

Who to Contact for Further Clarification/Questions: If you have any questions about the study, you are welcome to call the University of Ghana Principal-Investigator, Prof. Seth Adu-Afarwuah on Tel. # 030 396 4078, and he will be happy to answer your questions. For issues related to your participation in this study, you may call Nana Abena Kwaa Ansah Apatu, the ERC Administrator, Ghana Health Service Ethics Review Committee on 0503539896, or email ethics.research@ghs.gov.gh

---

CONSENT FORM

Title of Study: Randomized controlled trial of multiple micronutrient-fortified bouillon among women and children in two districts in the Northern Region of Ghana: opinions, attitudes, and acceptance towards the study bouillon among adult household members

PARTICIPANTS' STATEMENT

If the selected household member is 15-17 years and has ever been married, lived together with someone, divorced/separated or widowed, parental consent is not required. Obtain consent from the 15-17 year old participant.

I acknowledge that I have read or have had the purpose and contents of the Participants' Information Sheet read and satisfactorily explained to me in a language I understand (_______________ *language*) I fully understand the contents and any potential implications as well as my right to change my mind (i.e. withdraw from the research) even after I have signed this form.

I voluntarily agree to be part of this research.

Name of Participant...................................

Participants' Signature ....OR Thumb Print...............................

Date:.......................................

PARENTAL CONSENT FOR WOMEN 15-17 YEARS

If the selected household member is 15-17 years and lives with her parents (i.e. never been married and never lived together with someone), the Participants' Information Sheet should be read and consent procedures conducted in the presence of at least one parent.

I acknowledge that I have read or have had the purpose and contents of the Participants' Information Sheet read and satisfactorily explained to me in a language I understand (_______________ *language*) I fully understand the contents and any potential implications as well as my right to change my mind (i.e. withdraw my child/spouse from the research) even after I have signed this form.

I voluntarily agree for my child to be part of this research.

Name of Child ....

Name of Child's Parent..

Signature .... OR Thumb Print.................................

Date:.......................................

CHILD ASSENT

I acknowledge that I have read or have had the purpose and contents of the Participants' Information Sheet read and satisfactorily explained to me in a language I understand (_______________ *language*) I fully understand the contents and any potential implications as well as my right to change my mind (i.e. withdraw from the research) even after I have assented.

Do you voluntarily agree to be part of this research? YES ☐ NO ☐

Name of Child ....

Signature .... OR Thumb Print.................................

We may take photographs and videos of you, your family and your environment. However, before we can take photographs and videos, we need your consent. Your personal information will not be associated with any photographs and videos. As mentioned above, the photographs and videos will be used for teaching students, or when presenting research findings. If you agree or not agree that we can take photographs and videos, kindly indicate below:

	May we take photographs and videos? ***(Kindly tick if you want to be photographed and videoed or not)***
Yes	
No	

In case there is any leftover blood or urine sample, we would like to keep it for possible future research purposes. If you agree or not that your leftover sample may be kept for future possible research purposes, please indicate below:

May we keep your leftover urine or blood sample for future research? YES ☐ NO ☐

INTERPRETERS' STATEMENT

I interpreted the purpose and contents of the Participants' Information Sheet to the afore named participant to the best of my ability in the ___________________ language to his proper understanding.

All questions, appropriate clarifications sort by the participant and answers were also duly interpreted to his/her satisfaction.

Name of Interpreter... Signature of Interpreter...

Date:.......................... Contact Details...

STATEMENT OF WITNESS

I was present when the purpose and contents of the Participant Information Sheet was read and explained satisfactorily to the participant in the language he/she understood (_______________ *language*).

I confirm that he/she was given the opportunity to ask questions/seek clarifications and same were duly answered to his/her satisfaction before voluntarily agreeing to be part of the research.

Name:... Signature..

OR Thumb Print .... Date:...............................

INVESTIGATOR STATEMENT AND SIGNATURE

I certify that the participant has been given ample time to read and learn about the study. All questions and clarifications raised by the participant have been addressed.

Researcher's name... Signature ....

Date...
