# Supplementary material for: Perception and acceptance of micronutrient-Fortified Bouillon among Non-Index Household Members: A longitudinal sub-study nested within a randomized trial in Northern Ghana
Source: PLoS One. 2026 Apr 3;21(4):e0345106. doi: 10.1371/journal.pone.0345106 (PMC13048496; doi:10.1371/journal.pone.0345106)
Supplement: S1 File — S1 Table. Standardised factor loadings for the final two-factor confirmatory factor analysis model of perception and acceptance of study-supplied bouillon cubes among non-index household members. Note: This table shows the standardised factor loadings from the final two-factor confirmatory factor analysis model used to derive the perception and acceptance composite scores. The final two-factor model comprises 8 items for perception and 10 items for acceptance. Items with factor loadings ≥ 0.40 were retained in the final model. Negatively worded items (Q6, Q26, and Q27) were reverse coded before analysis. S2 Table. Baseline comparison of completers and non-completers at follow-up. This table summarizes demographic and household characteristics at baseline for participants who completed both time points and those who did not. S3 Table. Sensitivity analyses of individual- and household-level factors associated with perception (panel a) and acceptance (panel b) of study-supplied bouillon cubes among non-index household members. These analyses assess the robustness of the Bayesian mixed-effects model findings to alternative prior specifications. S4 Table. Intercoder reliability scores (ICR) calculated as Cohen’s Kappa and percentage agreement across six double-coded transcripts. This table summarizes coding consistency metrics for qualitative analysis, based on independent coding of six focus group discussion transcripts by two researchers. S5 File. Trial protocol (version 4, August 29, 2022). This protocol describes trial design, randomisation, intervention procedures, and data collection methods. S6 Table. Background characteristics of focus group discussion participants (n = 157). This table summarizes demographic and socioeconomic characteristics of qualitative participants. S7 File. Thematic analysis of 24 focus group discussions examining perceptions and acceptance of study-supplied bouillon cubes. This file presents the full qualitative analytic outputs, including [file pone.0345106.s001.zip › supplementary material_Plos one/S6_Table.docx.docx]

| **S6 Table. Background characteristics of focus group discussion participants (n = 157)** | | |
| --- | --- | --- |
| **Characteristics** | **Frequency** | **Percentage (%)** |
| **Age**, y | 157 | 43.5 ± 12.5 years |
| **Sex** |  |  |
| Male | 74 | 47.1 |
| Female | 83 | 52.9 |
| **Educational level** |  |  |
| No formal education | 106 | 67.5 |
| Primary | 15 | 9.6 |
| JHS | 14 | 8.9 |
| SHS & above | 22 | 14.0 |
| **Occupation** |  |  |
| Farming/Agricultural | 85 | 54.1 |
| Petty traders | 50 | 31.8 |
| Skilled labor | 20 | 12.7 |
| Government/private worker | 2 | 1.3 |
| **Household Position** |  |  |
| Household Head | 62 | 39.5 |
| Wife of household head | 70 | 44.6 |
| Daughter in-law of household head | 8 | 5.1 |
| Son of household head | 12 | 7.6 |
| Parent of household | 5 | 3.2 |
| **Note:** Values are reported as mean ± standard deviation (SD) for continuous variables and as counts and proportions for categorical variables. | | |
