# Supplementary material for: Perception and acceptance of micronutrient-Fortified Bouillon among Non-Index Household Members: A longitudinal sub-study nested within a randomized trial in Northern Ghana
Source: PLoS One. 2026 Apr 3;21(4):e0345106. doi: 10.1371/journal.pone.0345106 (PMC13048496; doi:10.1371/journal.pone.0345106)
Supplement: S1 File — S1 Table. Standardised factor loadings for the final two-factor confirmatory factor analysis model of perception and acceptance of study-supplied bouillon cubes among non-index household members. Note: This table shows the standardised factor loadings from the final two-factor confirmatory factor analysis model used to derive the perception and acceptance composite scores. The final two-factor model comprises 8 items for perception and 10 items for acceptance. Items with factor loadings ≥ 0.40 were retained in the final model. Negatively worded items (Q6, Q26, and Q27) were reverse coded before analysis. S2 Table. Baseline comparison of completers and non-completers at follow-up. This table summarizes demographic and household characteristics at baseline for participants who completed both time points and those who did not. S3 Table. Sensitivity analyses of individual- and household-level factors associated with perception (panel a) and acceptance (panel b) of study-supplied bouillon cubes among non-index household members. These analyses assess the robustness of the Bayesian mixed-effects model findings to alternative prior specifications. S4 Table. Intercoder reliability scores (ICR) calculated as Cohen’s Kappa and percentage agreement across six double-coded transcripts. This table summarizes coding consistency metrics for qualitative analysis, based on independent coding of six focus group discussion transcripts by two researchers. S5 File. Trial protocol (version 4, August 29, 2022). This protocol describes trial design, randomisation, intervention procedures, and data collection methods. S6 Table. Background characteristics of focus group discussion participants (n = 157). This table summarizes demographic and socioeconomic characteristics of qualitative participants. S7 File. Thematic analysis of 24 focus group discussions examining perceptions and acceptance of study-supplied bouillon cubes. This file presents the full qualitative analytic outputs, including [file pone.0345106.s001.zip › supplementary material_Plos one/S7_File.docx.docx]

| **S7 File. Thematic analysis of 24 focus group discussions examining perceptions and acceptance of study-supplied bouillon cubes** | |
| --- | --- |
| **Themes** | **Quotes** |
| Theme1: Improving health and well-being as drivers of acceptance | 1. “Since we started using the study-supplied bouillon cubes, I've noticed my children fall sick less often. Their energy levels have improved significantly." (Female, Landlord’s Wife, No Formal Education). 2. “The study cubes have been a blessing for my family. My elderly parents feel more energetic and less fatigued." (Male, Landlord, No Formal Education). 3. "We haven't had any health issues since switching to the study cubes. It feels good to know we're getting some nutrients, which are important to our health." (Female, Landlord’s Wife, JHS). |
| Theme 2: Sensory and Practical Experiences | 1. "The taste of the study cubes is perfect for our traditional soups and stews. It's not too salty or overpowering." (Female, Landlord’s Wife, SHS). 2. "We love the flavour of the study cubes. They make our meals delicious as the way we want the food to taste." (Male, Landlord, No Formal Education). 3. "The study cubes have a great taste that complements our cooking style. Everyone in the family enjoys the meals more now." (Female, Landlord’s Wife, JHS). |
| Theme 3: Cultural Compatibility and Cooking Habits | 1. "The study cubes fit perfectly with our traditional recipes. They don't change the original taste of our dishes." (Female, Landlord’s Wife, No Formal Education). 2. "We can use the study cubes in all our traditional meals without any issues. They respect our culinary heritage as when used to prepare any food, it tastes nicer." (Male, Landlord, No Formal Education). 3. "It's wonderful that the study cubes complement our traditional cooking methods. They enhance our dishes while preserving their authenticity." (Female, Landlord’s Wife, JHS). |
| Theme 4: Economic benefits of the cube influence their acceptance | 1. "The study-supplied cubes save us a lot of money. We don't need to buy additional cubes or even if we will by, we buy in small quantity." (Female, Landlord’s Wife, No Formal Education). 2. "Switching to the study cubes has been great for our budget. They're free and give as blood as we do not fall sick frequently any longer." (Male, Landlord, No Formal Education). 3. "We have cut down on healthcare costs since using the study-supplied cubes. They're an economical choice for our family because I do not get blood shortage anymore." (Female, Landlord’s Wife, SHS). |
| Theme 5: Challenges (Perceived Barriers to Acceptance) | 1. "My parents were hesitant to switch to the food cook with the study cubes. They prefer sticking to what they know." (Male, Landlord, No Formal Education). 2. "I'm still a bit wary about the long-term effects of consuming fortified products. I want to make sure they're safe for my family." (Female, Landlord’s Wife, SHS). 3. “Initially, I thought the study cube will serve as family planning and would reduce our sexual performing ability but as time but as time goes on, I realized I could perform better” (Male, Landlord, No Formal Education). 4. "Sometimes the intervention cubes change the colour of our soups to dark, especially the green leafy ones. It's not a major issue, but it's noticeable." (Female, Landlord’s Wife, JHS). |
| Sentimental analysis | Participants expressed both positive and negative sentiments toward the study-supplied bouillon cubes.   1. Positive sentiment: "We trust the fortified cubes completely. They have improved our health and well-being." (Female, Landlord’s Wife, No Formal Education). 2. Negative sentiment: "I'm just cautious about how the fortified cubes might affect us in the long run." (Male, Landlord, No Formal Education). |
| Sub-group analysis | 1. As a mother, I'm happy that the fortified cubes are keeping my children healthy." (Female, Landlord’s Wife, JHS). 2. The fortified cubes have made it easier for me to provide nutritious meals for my family without worrying about the cost." *(*Female, Landlord’s Wife, No Formal Education). 3. We might not understand all the science behind it, but we can see the positive changes in our health." (Female, Landlord’s Wife, No Formal Education). |
